# Immune and gene-expression profiling in estrogen receptor low and negative early breast cancer

**DOI:** 10.1093/jnci/djae178

**Published:** 2024-07-31

**Authors:** Davide Massa, Claudio Vernieri, Lorenzo Nicolè, Carmen Criscitiello, Florence Boissière-Michot, Séverine Guiu, Angélique Bobrie, Gaia Griguolo, Federica Miglietta, Andrea Vingiani, Riccardo Lobefaro, Beatrice Taurelli Salimbeni, Claudia Pinato, Francesca Schiavi, Silvia Brich, Carlo Pescia, Nicola Fusco, Giancarlo Pruneri, Matteo Fassan, Giuseppe Curigliano, Valentina Guarneri, William Jacot, Maria Vittoria Dieci

**Affiliations:** Oncology 2, Veneto Institute of Oncology IOV-IRCCS, Padova, Italy; Department of Surgery, Oncology and Gastroenterology (DiSCOG), University of Padova, Padova, Italy; Medical Oncology Department, Fondazione IRCCS Istituto Nazionale dei Tumori, Milan, Italy; IFOM ETS, The AIRC Institute of Molecular Oncology; Department of Pathology, Angelo Hospital, Mestre, Italy; Department of Oncology and Hemato-Oncology, University of Milan, Milan, Italy; Division of Early Drug Development for Innovative Therapy, European Institute of Oncology IRCCS, Milan, Italy; Translational Research Unit, Institut du Cancer de Montpellier, Montpellier, France; Department of Medical Oncology, Institut Régional Du Cancer de Montpellier (ICM), Montpellier, France; Institut de Recherche en Cancérologie de Montpellier, INSERM U1194, Montpellier University, Montpellier, France; Department of Medical Oncology, Institut Régional Du Cancer de Montpellier (ICM), Montpellier, France; Institut de Recherche en Cancérologie de Montpellier, INSERM U1194, Montpellier University, Montpellier, France; Oncology 2, Veneto Institute of Oncology IOV-IRCCS, Padova, Italy; Department of Surgery, Oncology and Gastroenterology (DiSCOG), University of Padova, Padova, Italy; Oncology 2, Veneto Institute of Oncology IOV-IRCCS, Padova, Italy; Department of Surgery, Oncology and Gastroenterology (DiSCOG), University of Padova, Padova, Italy; Department of Oncology and Hemato-Oncology, University of Milan, Milan, Italy; Department of Advanced Diagnostics, Fondazione IRCCS Istituto Nazionale dei Tumori, Milan, Italy; Medical Oncology Department, Fondazione IRCCS Istituto Nazionale dei Tumori, Milan, Italy; Division of Early Drug Development for Innovative Therapy, European Institute of Oncology IRCCS, Milan, Italy; UOSD Hereditary Tumors, Veneto Institute of Oncology IOV-IRCCS, Padova, Italy; UOSD Hereditary Tumors, Veneto Institute of Oncology IOV-IRCCS, Padova, Italy; Department of Advanced Diagnostics, Fondazione IRCCS Istituto Nazionale dei Tumori, Milan, Italy; Division of Pathology, European Institute of Oncology IRCCS, Milan, Italy; Department of Oncology and Hemato-Oncology, University of Milan, Milan, Italy; Division of Pathology, European Institute of Oncology IRCCS, Milan, Italy; Department of Oncology and Hemato-Oncology, University of Milan, Milan, Italy; Department of Advanced Diagnostics, Fondazione IRCCS Istituto Nazionale dei Tumori, Milan, Italy; Department of Medicine (DIMED), University of Padua, Padova, Italy; Veneto Institute of Oncology IOV—IRCCS, Padova, Italy; Department of Oncology and Hemato-Oncology, University of Milan, Milan, Italy; Division of Early Drug Development for Innovative Therapy, European Institute of Oncology IRCCS, Milan, Italy; Oncology 2, Veneto Institute of Oncology IOV-IRCCS, Padova, Italy; Department of Surgery, Oncology and Gastroenterology (DiSCOG), University of Padova, Padova, Italy; Translational Research Unit, Institut du Cancer de Montpellier, Montpellier, France; Department of Medical Oncology, Institut Régional Du Cancer de Montpellier (ICM), Montpellier, France; Institut de Recherche en Cancérologie de Montpellier, INSERM U1194, Montpellier University, Montpellier, France; Oncology 2, Veneto Institute of Oncology IOV-IRCCS, Padova, Italy; Department of Surgery, Oncology and Gastroenterology (DiSCOG), University of Padova, Padova, Italy

## Abstract

**Background:**

The cutoff of <1% positive cells to define estrogen receptor (ER) negativity by immunohistochemistry (IHC) in breast cancer (BC) is debated. We explored the tumor immune microenvironment and gene-expression profile of patients with early-stage HER2-negative ER-low (ER 1%-9%) BC, comparing them to ER-negative (ER <1%) and ER-intermediate (ER 10%-50%) tumors.

**Methods:**

Among 921 patients with early-stage I-III, ER ≤50%, HER2-negative BCs, tumors were classified as ER-negative (*n *=* *712), ER-low (*n *=* *128), or ER-intermediate (*n *=* *81). Tumor-infiltrating lymphocytes (TILs) were evaluated. CD8+, FOXP3+ cells, and PD-L1 status were assessed by IHC and quantified by digital pathology. We analyzed 776 BC-related genes in 116 samples. All tests were 2-sided at a <.05 significance level.

**Results:**

ER-low and ER-negative tumors exhibited similar median TILs, statistically significantly higher than ER-intermediate tumors. CD8/FOXP3 ratio and PD-L1 positivity rates were comparable between ER-low and ER-negative groups. These groups showed similar enrichment in basal-like intrinsic subtypes and comparable expression of immune-related genes. ER-low and ER-intermediate tumors showed significant transcriptomic differences. High TILs (≥30%) were associated with improved relapse-free survival (RFS) in ER-low (5-year RFS 78.6% vs 66.2%, log-rank *P *=* *.033, hazard ratio [HR] 0.37 [95% CI = 0.15 to 0.96]) and ER-negative patients (5-year RFS 85.2% vs 69.8%, log-rank *P *<* *.001, HR 0.41 [95% CI = 0.27 to 0.60]).

**Conclusions:**

ER-low and ER-negative tumors are similar biological and molecular entities, supporting their comparable clinical outcomes and treatment responses, including to immunotherapy. Our findings contribute to the growing evidence calling for a reevaluation of ER-positive BC classification and management, aligning ER-low and ER-negative tumors more closely.

Estrogen receptor (ER) expression serves as the main predictive biomarker for endocrine therapy (ET) responsiveness in breast cancer (BC). The current threshold for ER positivity, defined by immunohistochemistry (IHC) as ≥1% of positively stained cancer cells (1), is debated. Patients with low ER levels (1% to 9%, ER-low) derive limited benefit from adjuvant ET (2-9), and yet share similar clinicopathological characteristics (3,4,10), prognosis (11,12), response rates to neoadjuvant chemotherapy (NACT) (11,13), and prognostic effect of pathological complete response (pCR) (14) as ER <1%/HER2-negative (ER-neg) BC. Biological data substantiate these clinical similarities, because ER-low and ER-neg BC show similar gene-expression profiles (GEP) such as intrinsic molecular subtyping (15-18) and prognostic genomic assays (18), and comparable germline BRCA mutation (19,20).

The immunological features of ER-low BC remain largely underexplored. ER-neg BC typically exhibits a “hot” tumor microenvironment (TME), which contrasts the immune-suppressive features of ER-positive tumors (21). Although tumor-infiltrating lymphocytes (TILs) have a positive prognostic significance in ER-neg BC (22,23), their impact in ER-positive BC patients remains ambiguous (24), with some studies suggesting a detrimental effect (23,25). Preliminary data indicate no significant differences in TME between ER-neg and ER-low tumors (4,16), but the prognostic value of TILs in ER-low BC has yet to be defined.

Immunotherapy has become a standard treatment for ER-neg BC (26,27), but its efficacy in ER-positive BC is less pronounced (28-35), benefiting only a few patients (32-35). Regarding the subset of ER-low BCs, studies suggest a similar antitumor activity of immune checkpoint inhibitors (ICIs) to that observed in ER-neg BC (36), and higher than seen in ER-positive patients (ER ≥1%) (34). However, ER-low patients were excluded from pivotal trials leading to the approval of ICIs for ER-neg BC (26,27), leading to a lack of access to promising immunotherapy-based treatments.

Given the uncertainties surrounding the impact of varying ER-expression levels on immune dynamics, paralleled by the potential to modulate them with immune-modulatory strategies such ICIs (34-36), there is an urgent, unmet need for the poor-prognosis subset of ER-low patients.

This multicentric study aims to address these gaps by comparing the TME and GEP in early-stage (I-III) HER2-negative BC by ER status and investigate TILs’ prognostic significance in ER-low tumors.

## Methods

### Population

This study includes 921 patients with early-stage (I-III), HER2-negative BC from 4 institutions: Istituto Oncologico Veneto (IOV) Padova, Italy (*n *=* *451); Montpellier Cancer Institute (MCI), Montpellier, France (*n *=* *223); Istituto Nazionale Tumori (INT), Milano, Italy (*n *=* *178); and Istituto Europeo di Oncologia (IEO), Milano, Italy (*n *=* *69). Patients were selected based on an expression of ER between 0% and 50% of cancer cells by IHC, according to local review. Tumors were classified as ER-neg (ER 0%, n = 712), ER-low (ER 1%-9%, *n *=* *128), or ER-intermediate (ER-int) (ER 10%-50%, *n *=* *81, included as a control cohort). Allowed progesterone (PgR) levels were up to 10% for ER-neg and ER-low cases. ER-neg and ER-low cases from IOV, MCI, and INT were consecutively treated (March 2000 to December 2021, June 2002 to November 2012, and December 2005 to May 2022, respectively). Supplementary Figure 1 (available online) shows patient disposition.

Patients with ER-int and all patients from IEO were derived from nonconsecutive cohorts enriched in patients who experienced disease relapse; these patients were excluded from survival analyses.

Clinicopathological, treatment, and follow-up data were collected.

### Pathology

Treatment-naïve formalin-fixed paraffin-embedded (FFPE) tumor samples were collected: surgery specimens for patients treated with primary surgery and pretreatment core-biopsies for patients treated with neoadjuvant treatment.

All IHC protocols relevant to this study are reported as Supplementary Material (available online).

ER status was locally reviewed on previously stained IHC slides by dedicated breast pathologists.

HER2 status was scored according to ASCO/CAP recommendations in place at the time of diagnosis.

Blinded histopathological assessment of stromal TILs density on hematoxylin-eosin stained whole-slides (WS) was conducted locally by dedicated pathologists, following standardized guidelines (37). TILs were evaluated both as continuous and as categorical variables at the ≥30% cutoff validated in triple-negative BC (22,38).

To investigate the existence of more granular differences in TILs’ composition across two cohorts of ER-low and ER-neg tumors, we evaluated the density of CD8+ cells, the primary mediators of tumor killing, FOXP3+ T regulatory cells, which tamper antitumor immune responses by exerting strong immunosuppressive functions, and the immune-checkpoint PD-L1. Since an enhanced FOXP3+ cell infiltrate may contrast the antitumor activity of CD8+ cells (39), we used the ratio of CD8/FOXP3 positive cells to infer the polarization of the TME toward an immune-active or an immune-suppressive state (40). CD8/FOXP3 and PD-L1 IHC staining was evaluated only in ER-neg and ER-low samples (n = 477), sourced from IOV and MCI. At IOV, samples were handled as WS, whereas MCI employed tissue-microarray (TMA). For each case, consecutive slides were locally stained for CD8, FOXP3, and PD-L1 and then scanned using a NanoZoomer C12740 digital scanner. All digital slides were centrally evaluated at IOV for CD8, FOXP3, and PD-L1 metrics using Visiopharm software applications, following a previously described digital pathology workflow (41). Scanned slides from IOV were aligned with a MNF116 stained slide from the same sample to define the stromal compartment of the tumor. The densities of CD8+ and FOXP3+ cells were measured as number of positive cells/mm^2^. At IOV, this measurement was performed in the stromal area of the tumor. For MCI cases, the intratumoral area of TMA foci was considered. To account for outliers, the CD8/FOXP3 density ratio was log-transformed. PD-L1 expression was evaluated on tumor-infiltrating immune cells (IC score) with the SP142 clone (Ventana), and cases with immunoreactive immune cells covering ≥1% of the tumor area were considered positive.

### Gene expression

Gene-expression analyses were performed locally at IOV and INT. Pathologists reviewed FFPE samples for tumor tissue quality and quantity. From samples with adequate material (>40% of tumor cells), a cohort of ER-low and ER-neg cases matched for age (<50, 50-65, or >65 years old), histotype (ductal, lobular, or other), and stage (I, II, or III) were identified. A control cohort of unmatched ER-int cases was included.

RNA extracted from FFPE was used to measure gene expression using the Breast Cancer 360 Panel on the nCounter platform (NanoString Technologies, Inc, Seattle, WA, USA) covering 776 genes from different independent signatures, including the PAM50 signature (Supplementary Material, available online). Gene-expression data were normalized using a ratio of the expression value to the geometric mean of the housekeeper genes of the PAM50 signature. Data were then log2 transformed. Intrinsic molecular subtyping was determined using the previously reported PAM50 subtype predictor (42). An unpaired 2-class SAM analysis with a 5% false discovery rate (FDR) was used to identify genes differentially expressed in different subgroups.

### Statistical analysis

Statistical analyses were performed using IBM software SPSS v.29.0 and R (version 4.2.1); all tests were 2-sided, and an alpha < 0.05 significance level was used.

The association between variables was evaluated using the Mann-Whitney or Kruskal-Wallis nonparametric tests for continuous variables, and the χ^2^ test or Fisher exact test for categorical variables, as appropriate.

Relapse-free survival (RFS) was defined as the time from diagnosis to relapse or death from any cause, and overall survival (OS) as the time from diagnosis to death from any cause. Patients without events were censored at the time of the last follow-up.

The Kaplan-Meier method was used to estimate survival curves, the log-rank test to compare survival curves, and the Cox regression model to calculate hazard ratios (HR) and 95% confidence intervals (95% CI).

### Ethical considerations

Tumor samples were collected after approval from the Institutional Review Board of each participating center and in accordance with the Declaration of Helsinki. Written consent was obtained from each participant who was alive at the time of study entry.

## Results

### Patients’ characteristics

We included a total of 921 patients: 712 patients with ER-neg, 128 with ER-low, and 81 with ER-int BC (Supplementary Figure 1, available online). Table 1 presents the clinicopathological data of the two primary patient groups: ER-low and ER-neg.

**Table 1.** Clinicopathological data of patients with estrogen receptor (ER)-low (ER 1%-9%) and ER-negative (ER-neg, <1%) tumors

Clinicopathological characteristics		ER-neg (*n *=* *712)	ER-low (*n *=* *128)	*P*
		*N* (%)	*N* (%)	
Age, years	Median (IQR)	54 (45-64)	53 (44-67)	.713
	Range	22-98	29-90	
Histology	Ductal/NOS	614 (88.1%)	113 (89.0%)	.022
	Lobular	22 (3.2%)	11 (8.7%)	
	Apocrine	17 (2.4%)	0	
	Metaplastic	9 (1.3%)	0	
	Medullary	4 (0.6%)	0	
	Other	31 (4.4%)	3 (2.3%)	
Grade	1	4 (0.6%)	0	.243
	2	78 (11.3%)	20 (16.4%)	
	3	607 (88.1%)	102 (83.6%)	
PgR, %	Median (IQR)	0 (0-0)	0 (0-1)	<.001
	Range	0-5	0-9	
HER2 status	0	459 (64.5%)	57 (44.9%)	<.001
	1+	182 (25.5%)	55 (43.3%)	
	2+/ISH unamplified	71 (10.0%)	15 (11.8%)	
Ki67, %	Median (IQR)	60 (35-70)	60 (35-75)	.659
	Range	1-95	5-95	
Stage	I	212 (29.9%)	43 (33.9%)	.230
	II	402 (56.7%)	62 (48.8%)	
	III	95 (13.4%)	22 (17.3%)	
Nodal status	Negative	386 (60.8%)	68 (54.8%)	.217
	Positive	249 (39.2%)	56 (45.2%)	
Neoadjuvant CT	No	411 (57.7%)	94 (73.4%)	<.001
	Yes	301 (42.3%)	34 (26.6%)	
Neoadjuvant carboplatin	No	117 (48.8%)	18 (62.1%)	.151
	Yes	127 (52.0%)	11 (37.9%)	
Neoadjuvant anthracyclines	No	4 (1.6%)	2 (6.9%)	.125
	Yes	240 (98.4%)	27 (93.1%)	
Neoadjuvant taxanes	No	1 (0.4%)	0	>.999
	Yes	244 (99.6%)	29 (100%)	
Response to neoadjuvant treatment	Residual disease	177 (58.8%)	20 (58.8%)	.998
	pCR	124 (41.2%)	14 (41.2%)	
Adjuvant CT	No	280 (39.3%)	44 (34.4%)	.289
	Yes	432 (60.7%)	84 (65.6%)	
Adjuvant CT after NACT (residual disease)	No	115 (65.0%)	13 (65.0%)	.998
	Yes	62 (35.0%)	7 (35.0%)	
CT exposure	No	43 (6.0%)	17 (13.3%)	.003
	Yes	669 (94.0%)	111 (86.7%)	
Endocrine therapy	No	476 (94.6%)	71 (67.6%)	<.001
	Yes	27 (5.4%)^a^	34 (32.4%)	
Adjuvant radiotherapy	No	117(31.5%)	16 (38.1%)	.389
	Yes	254 (68.5%)	26 (61.9%)	

a A limited number of ER-neg patients received endocrine therapy, probably due to some degree of ER positivity on residual disease after NACT. ER-neg = ER-negative; IQR = interquartile range; NOS = not otherwise specified; ER = estrogen receptor; PgR = progesterone receptor; ISH = in situ hybridization; pCR = pathological complete response (ypT0/is ypN0); CT = chemotherapy.

Statistics: χ^2^, or Fisher exact test when appropriate, was employed to test the distribution of categorical variables; Mann-Whitney nonparametric test was used to compare the distribution of continuous variables.

Compared to patients with ER-neg BC, those with ER-low tumors more commonly had lobular histology and were less likely to have HER2-0 status, possibly due to a positive association between HER2-signaling and ER-expression. No differences in key clinic-pathological features such as stage, nodal status, grade, or proliferation rate were noted. ER-low patients were less frequently treated with chemotherapy, including NACT, but received ET more frequently.

The non-consecutively treated cohort of patients with ER-int tumors, compared with ER-neg and ER-low, showed differences in several clinic-pathological characteristics (Supplementary Table 1, available online), which may be related partly to different inherent biology of ER-int tumors and partly to the selection procedure (cohort enriched in patients with disease relapse).

Survival analyses revealed no significant differences between ER-low and ER-neg patients both in terms of RFS (5 years RFS 70.9% vs 74.9%, log-rank *P = *.181; HR 1.26 [95% CI = 0.90 to 1.78]) and OS (79.3% vs 82.2%, log-rank *P *=* *.223; HR 1.27 [95% CI = 0.86 to 1.87]) (Supplementary Figure 2, A and B, available online). This observation was consistent at a 60-months landmark analysis, where no difference was noted for both RFS (log-rank *P *=* *.105; HR 1.84 [95% CI = 0.87 to 3.90]) and OS (log-rank *P *=* *.202; HR 1.57 [95% CI = 0.78 to 3.15]) (Supplementary Figure 2, C and D, available online), despite numerically higher rates of late distant relapses in the ER-low subgroup (Supplementary Table 2, available online). Similar results were obtained when directly comparing the outcome of ER-low and ER-neg among the selected group of patients exposed to systemic chemotherapy (5 years RFS 72.0% vs 76.7%, log-rank *P *=* *.182; HR 1.29 [95% CI = 0.89 to 1.87]); 5 years OS 80.2% vs 83.9%, log-rank *P *=* *.308; HR 1.25 [95% CI = 0.81 to 1.92]) (Supplementary Figure 3, available online).

### TILs density according to ER status

We assessed TILs in 846 samples, 647 ER-neg, 119 ER-low, and 80 ER-int (Supplementary Figure 1, available online).

**Figure 1.**
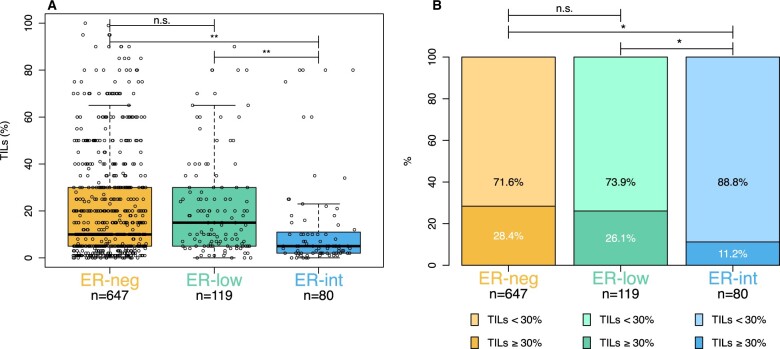
Distribution of tumor-infiltrating lymphocytes (TILs) as a continuous (1% increase) (**A**) and categorical variable (≥30% cutoff) (**B**), stratified by estrogen receptor (ER) status: ER-negative (ER-neg, ER <1%), ER-low (ER 1%-9%), and ER-intermediate (ER-int, ER 10%-50%). TILs = tumor-infiltrating lymphocytes; *P < .05, **P < .001; n.s. = nonsignificant.

TILs were similar in ER-neg and ER-low BC (median 10%, interquartile range [IQR] [5-30] vs 15%, [5-30]; *P *> .999) (Figure 1, A). In contrast, TILs were statistically significantly lower in ER-int (median 5%, IQR [2-11]) compared with both ER-low (*P *<* *.001) and ER-neg (*P *<* *.001) BC specimens (Figure 1, A). To address the potential influence of tumor-intrinsic features on our analysis, we evaluated the distribution of TILs within ER status according to stage, grade, and Ki67, showing similar influence of grade and Ki67 on TIL density in both ER-neg and ER-low tumors (Supplementary Table 3, available online).

Similar proportions of patients with high TILs (≥30%) were observed in ER-neg and ER-low groups (28.4% vs 26.1%, *P *=* *.594). In contrast, ER-int samples showed a lower proportion of patients with high TILs (11.2%) compared with both ER-neg (*P *=* *.001) and ER-low groups (*P *=* *.011) (Figure 1, B). These findings remained consistent when we separately analyzed samples from each participating institution (Supplementary Figures 4 and 5, available online).

To further explore TILs density within ER-int tumors, we divided them into two subcategories: ER 10%-30% and ER 31%-50%. Our analysis indicated that tumors with ER 10%-30% showed no significant difference in TILs density (median 9%, IQR [3-23]), compared with ER-neg (*P *> .999) and ER-low tumors (*P *= .678). Instead, tumors with the highest spectrum of ER-expression (31%-50%) had lower TILs (median 4% [IQR 2-8]) compared with both ER-neg (*P *< .001) and ER-low tumors (*P *< .001), but not statistically different from tumors with ER 10%-30% (*P *= .116) (Supplementary Figure 6, available online).

### Immune cell densities and PD-L1 expression

**Figure 2.**
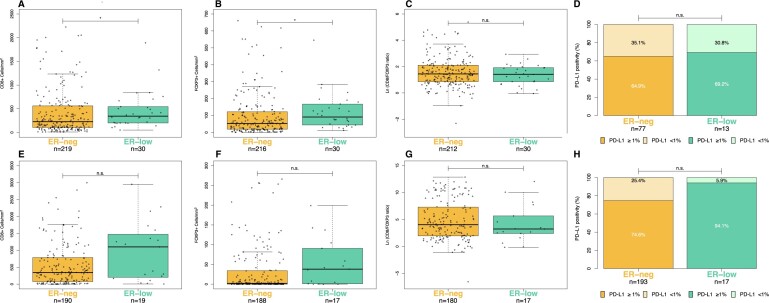
Distribution of CD8+ cells (cells/mm^2^), FOXP3+ cells (cells/mm^2^), the log-transformed CD8/FOXP3 ratio, and the rate of positive PD-L1 expression (≥1% cutoff) across different estrogen-receptor (ER) statuses: ER-neg (ER neg, ER <1%) and ER-low (ER 1%-9%). The top half (**2A-D**) presents biomarker data from the Istituto Oncologico Veneto cohort (full-face slides, stromal compartment), whereas the bottom half (**2E-H**) features data from the Montpellier Cancer Institute cohort (tissue micro-array, intratumoral foci). Note: To improve readability, the y-axis in Figure 2, A, B, E, and F is truncated. ER-neg = ER-negative (ER <1%), ER-low (ER 1%-9%), *P < .05; n.s. = nonsignificant.

ER-low tumors showed higher densities of both CD8+ and FOXP3+ cells/mm^2^ compared with ER-neg BCs, and this difference reached statistical significance in the IOV cohort (*P = *.040 and *P = *.011, respectively) (Figure 2, A and B) but not in the smaller MCI cohort (*P *=* *.081 and *P *=* *.057, respectively) (Figure 2, E and F). On the other hand, the log-transformed CD8/FOXP3 ratio was similar in ER-low vs ER-neg tumors (IOV: median 1.45, IQR [0.86-2.11] vs 1.42 [0.86-1.92], *P *=* *.504; MCI: 4.04 IQR [1.97-7.30] vs 3.24 IQR [2.42-5.67] *P *=* *.400, Figure 2, C and G), and the two cohorts were also characterized by a similar rate of PD-L1 positive expression (IOV: 69.2% vs 64.9% *P* > .999; MCI: 94.1% vs 74.6%, *P *=* *.080, Figure 2, D and H).

### Prognostic impact of TILs in ER-low and ER-neg BC

We examined the prognostic relevance of TILs according to ER status in 647 ER-neg and 105 ER-low cases. The median follow-up time was 8.2 years (95% CI = 7.8 to 8.7 years).

At univariate analysis, each 1% increase in TILs corresponded to a 2% reduction in the risk of RFS-event in both ER-neg (HR 0.98 [95% CI = 0.98 to 0.99], *P *<* *.001) and ER-low (HR 0.98 [95% CI = 0.96 to 1.00], *P = *.033) cohorts (Table 2). We also found a 2% reduction in the risk of death for each 1% TILs increase in both patient cohorts (ER-neg: HR 0.98, 95% CI [0.97 to 0.99], *P *<* *.001; ER-low: HR 0.98, 95% CI [0.96 to 1.00], *P = *.062).

**Table 2.** Univariate and multivariate Cox analyses for relapse-free survival and overall survival in patients with estrogen receptor (ER)-negative (ER-neg, ER <1%) and ER-low (ER 1%-9%) breast cancer

		Relapse-free survival								Overall survival							
		ER-neg				ER-low				ER-neg				ER-low			
		Univariate		Multivariate		Univariate		Multivariate		Univariate		Multivariate		Univariate		Multivariate	
		HR (95% CI)	*P*	HR (95% CI)	*P*	HR (95% CI)	*P*	HR (95% CI)	*P*	HR (95% CI)	*P*	HR (95% CI)	*P*	HR (95% CI)	*P*	HR (95% CI)	*P*
Age (Cont.)		1.02 (1.01 to 1.04)	*<.001*	1.02 (1.01 to 1.03)	*.003*	1.00 (0.98 to 1.03)	*.705*	1.01 (0.98 to 1.03)	*.706*	1.04 (1.02 to 1.05)	*<.001*	1.03 (1.01 to 1.04)	*<.001*	1.02 (0.98 to 1.05)	*.087*	1.02 (0.99 to 1.05)	*.181*
Grade	1-2	Ref		–	–	Ref		–	–	Ref		–	–	Ref		–	–
	3	0.78 (0.53 to 1.16)	*.219*	–	–	0.52 (0.24 to 1.11)	*.089*	–	–	0.68 (0.45 to 1.04)	*.075*	–	–	0.77 (0.29 to 2.03)	*0.594*	–	–
Ki67 (Cont.)		1.00 (0.99 to 1.00)	*.198*	–	–	0.99 (0.97 to 1.01)	*.218*	–	–	1.00 (0.99 to 1.01)	*.369*	–	–	0.99 (0.97 to 1.01)	*0.195*	–	–
Stage	I	Ref		Ref		Ref		Ref		Ref		Ref		Ref		Ref	
	II	2.05 (1.40 to 3.02)	*<.001*	2.45 (1.67 to 3.72)	*<.001*	1.08 (0.52 to 2.22)	*.844*	1.11 (0.51 to 2.40)	*.801*	1.87 (1.22 to 2.89)	*.004*	2.12 (1.37 to 3.29)	*<.001*	1.13 (0.51 to 2.48)	*.770*	1.43 (0.62 to 3.29)	*.408*
	III	4.57 (2.93 to 7.12)	*<.001*	5.21 (3.27 to 8.32)	*<.001*	1.49 (0.61 to 3.68)	*.383*	1.43 (0.52 to 3.94)	*.494*	4.32 (2.64 to 7.06)	*<.001*	4.82 (2.91 to 8.01)	*<.001*	1.06 (0.36 to 3.16)	*.919*	1.39 (0.42 to 4.60)	*.594*
CT exposure	No	Ref		Ref		Ref		Ref		Ref		Ref		Ref		Ref	
	Yes	0.39 (0.25 to 0.60)	*<.001*	0.44 (0.27 to 0.71)	*<.001*	0.56 (0.23 to 1.35)	*.196*	0.59 (0.19 to 1.86)	*.369*	0.31 (0.20 to 0.48)	*<.001*	0.41 (0.24 to 0.69)	*<.001*	0.40 (0.16 to 0.99)	*.047*	0.64 (0.20 to 2.08)	*.455*
TILs (1% incr.)		0.98 (0.98 to 0.99	*<.001*	0.99 (0.98 to 0.99)	*<.001*	0.98 (0.96 to 1.00)	*.020*	0.97 (0.96 to 1.00)	*.037*	0.98 (0.97 to 0.99)	*<.001*	0.99 (0.98 to 1.00)	*.002*	0.98 (0.96 to 1.00)	*.062*	0.98 (0.95 to 1.00)	*.066*

All the variables that resulted significantly associated with outcome in univariate analysis at least in one group (ER-neg or ER-low) were included in multivariate analyses for both groups. CI= confidence interval; Cont. = continuous; HR = hazard ratio; Incr. = increase; CT = chemotherapy; TILs = tumor-infiltrating lymphocytes.

**Figure 3.**
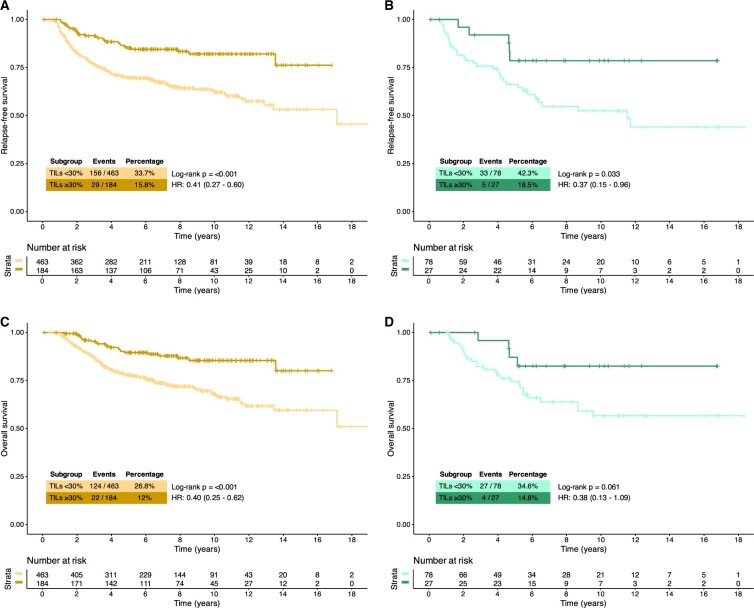
Kaplan-Meier survival curves in patients with estrogen receptor (ER)-negative (ER-neg) and ER-low breast cancer (A, relapse-free survival in ER-neg; B, relapse-free survival in ER-low; C, overall survival in ER-neg; D, overall survival in ER-low) according to tumor-infiltrating lymphocytes (TILs) at ≥30% cutoff. HR = hazard ratio

When TILs were dichotomized based on a ≥30% cutoff (Figure 3, A and B), we found that high TILs were associated with statistically significantly improved RFS in both ER-neg (5 year RFS 85.2% vs 69.8%, log-rank *P *<* *.001, HR 0.41 [95% CI = 0.27 to 0.60]) and ER-low (5-year RFS 78.6% vs 66.2%, log-rank *P = *.033, HR 0.37 [95% CI = 0.15 to 0.96]) cohorts. We found similar findings when OS was used as a clinical outcome, with results reaching statistical significance for ER-neg (5-year OS 89.6% vs 78.0%, log-rank *P *<* *.001; HR 0.40 [95% CI = 0.25 to 0.62]) and pointing to the same direction for ER-low (5 year 87.1% vs 74.5%, log-rank *P *=* *.061; HR 0.38 [95% CI = 0.13 to 1.09]) (Figure 3, C and D).

Results of univariate analyses were confirmed by multivariate analyses adjusting for age, stage, chemotherapy exposure (Table 2), and when factoring ER expression (ER-neg vs ER-low) as a covariate (Supplementary Table 4, available online).

### Gene-expression analysis

Gene-expression analyses were performed on 65 ER-low cases, matched to 39 ER-neg tumors. Twelve ER-int samples served as unmatched controls.

**Figure 4.**
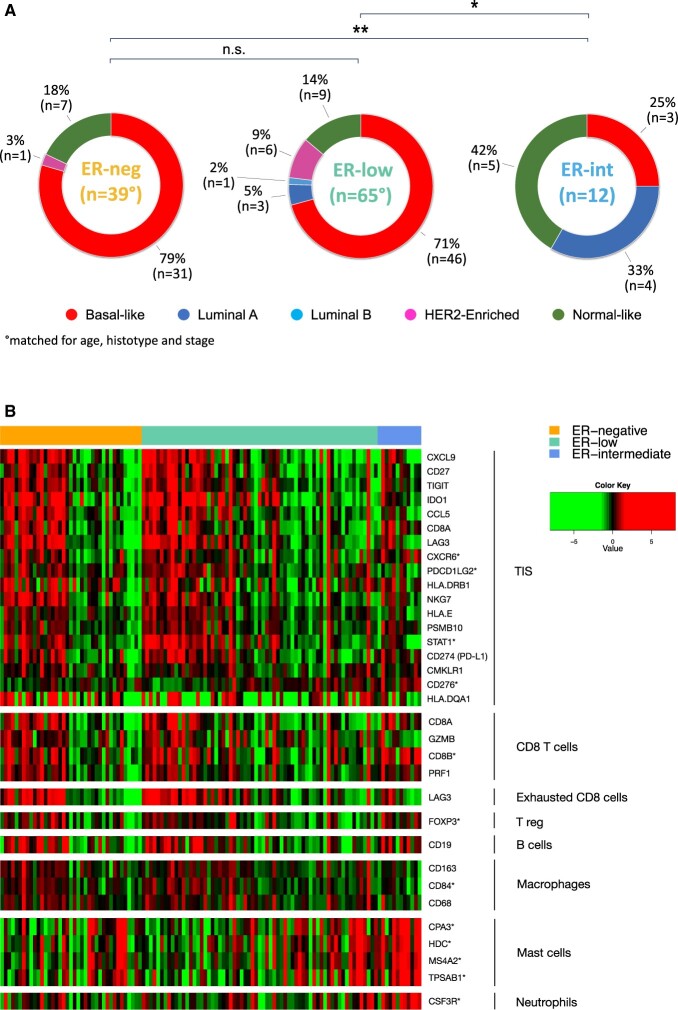
A) Distribution of PAM50 intrinsic subtypes in patients with estrogen receptor (ER)-negative (ER-neg, ER <1%), ER-low (ER 1%-9%), and ER-intermediate (ER-int, ER 10%-50%) breast cancer; asterisks (*P < .05, **P < .001) mark a statistically significant difference in the distribution of subtypes. **B)** Heatmap illustrating differential expression of immune genes, clustered by ER status (ER-neg, ER-low, and ER-int) and ordered according to decreasing density of TILs: colors indicate mRNA expression levels, higher in the case of red, and lower for green. The graph is further segmented according to genes associated with tumor inflammation signature (TIS) and various immune cells, such as CD8 T cells, exhausted T cells, Tregs, B cells, macrophages, mast cells, and neutrophils. An asterisk (*) marks gene differentially expressed in ER-low vs ER-int samples as identified by SAM analysis (FDR <5%). ER = estrogen receptor; ER-neg = ER-negative (ER neg, ER <1%); ER-low (ER 1%-9%); ER-int = ER-intermediate (ER 10%-50%); FDR = false-discovery rate; n.s. = non significant; SAM = significance analysis of microarrays; TIS = tumor inflammation signature.

Both ER-neg and ER-low tumors exhibited a similar distribution in PAM50-intrinsic subtypes (*P *=* *.396), primarily featuring basal-like tumors (79%, *n *=* *31, and 71%, *n *=* *46, respectively) (Figure 4, A). Conversely, the ER-int group differed statistically significantly from both ER-low (*P = *.002) and ER-neg patients (*P *<* *.001), with basal-like tumors making up only 25% of the cases.

Basal-like subtype showed statistically significantly higher TILs compared with other subtypes in both ER-low (median 20%, range [0-80%] vs 6% [1-40%], *P *<* *.001) and ER-int samples (53% [25-80%] vs 5% [0-10%], *P *=* *.036), whereas no significant difference in TILs was observed in ER-neg tumors (*P *=* *.503).

SAM analysis of 776 genes revealed that only three were differentially expressed in ER-low compared with ER-neg tumors (*GATA3*, upregulated; *EDN1* and *PROM1*, downregulated) (Supplementary Table 5, available online). When focusing on basal-like tumors (*n *=* *77), only *EDN1* and *PROM1* genes remained differentially downregulated in ER-low (Supplementary Table 6, available online). In contrast, ER-low samples showed a distinct expression pattern compared with ER-int, with a statistically significantly higher expression of 53 genes and a lower expression of 398 genes (Supplementary Table 7, available online).

Comparing the expression of 164 immune-related genes in ER-low and ER-neg tumors, we found no significant differences in the expression of genes related to antigen presentation, cytokine and chemokine signaling, immune infiltration, TGF-beta signaling (Figure 4, B), or the characterization of immune cells (functionally annotated in Supplementary Table 8, available online). However, 86 genes, including 4 mast-cell-related genes, showed statistically significantly different expression levels between ER-low to ER-int tumors (Supplementary Table 9, available online).

## Discussion

Our multicentric study reveals that ER-low and ER-neg BCs share similar immune and gene expression characteristics, differing significantly from ER-int tumors. We uniquely demonstrated that high TILs in ER-low BC independently indicate a positive prognosis.

Our clinical outcome analyses showed no significant differences in RFS and OS between the ER-low and ER-negative cohorts, with even a numerically higher rate of relapses in ER-low tumors. Importantly, both groups exhibited comparable pCR rates when treated with NACT, aligning with previous studies (11,13,15,43-45) and contrasting sharply with the limited response rates generally seen in hormone-receptor-positive/HER2- BC (46, 47).

Our observation that ER-low and ER-neg BCs have similar TILs density, which is instead lower in ER-int BC specimens, is remarkable. Indeed, ER-neg BC specimens typically exhibit higher levels of TILs when compared to hormone-receptor-positive/HER2-negative BCs (23,48), owing to the generally higher immunogenic background of ER-neg tumors, which contrasts the “cold” immune-suppressive TME often observed in hormone-receptor-positive/HER2-negative BC (21,49,50). Notably, in this study, we found that high levels of TILs were comparably associated with a more favorable prognosis in both ER-neg and ER-low BC patients.

Consistently, we observed a similar ratio of CD8/FOXP3 positive cells in ER-low and ER-neg tumor specimens, suggesting a similar polarization of the TME (40). Again in contrast with the acknowledged low expression of PD-L1 in hormone-receptor-positive BCs (51), we also identified a high positivity rate in ER-low tumors, akin to ER-neg. Together, these data support the existence of similar immune dynamics across ER-expression levels up to 9%.

In our gene-expression analysis, ER-low and ER-neg BC samples showed no major transcriptional differences, including an enrichment in basal-like subtypes, consistent with findings in previous studies (15-18). Notably, no immune-related gene was differentially expressed between these groups. In contrast, ER-int tumors displayed a distinct immune profile, characterized by increased expression of several mast cell-related genes. This aligns with previous findings that higher ER levels correlated with mast cell presence (16,52), a trait potentially contributing to the promotion of a luminal phenotype (53,54).

Our data provide strong evidence that ER-low and ER-neg are immunologically and biologically similar entities. Although ER IHC-staining was conceived as a predictive biomarker for ET benefit, the relationship between ER nuclear expression and specific immune-suppressive features typical of ER-positive tumors (55), which may dampen responses to ICIs (21), appears to be nonlinear. Our study shows that tumors with ER levels up to 9% exhibit similar CD8/FOXP3 ratio, PD-L1 expression, and GEP, indicating a marked immune and molecular divergence beginning at ER-int expression levels. This partially aligns with a recent report confirming similar immune features in ER-neg and ER-low BC (16). However, that study, despite reporting a higher prevalence of basal-like subtypes in ER-neg and ER-low compared with ER-int tumors, did not observe significant differences in TME across a broader range of ER expression levels (0% to 50%). This observation aligns with our exploratory observation of similar TIL density in patients with ER up to 30%, corroborating the potential of identifying a group of immune-active tumors within the broader ER-positive spectrum.

The biologic heterogeneity within ER-positive/HER2-negative BCs plays a critical role in determining the efficacy of CT, ET (56,57), and ICIs (34-36,58).

Luminal tumors are sensitive to ET (59,60), whereas basal-like tumors resist ET and cyclin-dependent kinase 4/6 inhibitors (61) but are more responsive to chemotherapy (62-65). Molecular subtyping combined with immune features may help identify ER-expressing tumors sensitive to immunotherapy across ER levels (32,66,67). For instance, in the I-SPY2 trial, among ER-positive/HER2-negative BC classified as high-risk on MammaPrint, a basal-like intrinsic subtype was associated with a 67% pCR with pembrolizumab added to NACT (66). Furthermore, the GIADA trial (32) reported that the co-occurrence of a basal-like intrinsic subtype and high TILs in premenopausal patients with ER ≥10%/HER2-negative BC and a luminal B-like IHC profile could accurately predict pCR after ICI-based neoadjuvant treatment and ET. Exploring the presence of this immune-responsive basal-like/high-TILs phenotype in our cohort, we observed higher TILs in ER-low and ER-int BC with basal-like tumors compared with non-basal-like tumors.

Recent trials have underscored a distinct activity of ICIs in the ER-low subgroups (34-36), mirroring those of ER-neg patients (36,68) and supported by the similar immune dynamics seen in our study. The NeoPACT phase II trial demonstrated comparable pCR rates in ER-low (56%) and ER-neg patients (58%) with pembrolizumab-NACT (36). In the Keynote-756 trial, ER-low patients experienced a 25.6% increase in pCR rates from the addition of pembrolizumab to NACT, much higher than the mere 8% seen in patients with ER 10%-100% (34). Strikingly, this delta is even larger than the 13.6% increase shown in the Keynote-522 trial, which led to pembrolizumab’s approval for ER-neg breast cancer (26). Similarly, the addition of nivolumab to NACT in the Checkmate 7FL trial resulted in a 27.0% increase in pCR rate in ER-low patients and 29.3% in those with ER ≤50%, compared to just 7.4% increase in patients with ER >50% (35). A correlation between pCR rates and the expression of PD-L1 (34, 35) and TILs (35) was seen in those trials across the spectrum of ER-positive tumors, which suggests the potential of a biologically informed, response-oriented subtyping of BC (67).

Our study has several strengths. It represents the largest study to provide immune-transcriptomic profiling of patients with ER-low BC, offering significant insights into this understudied population. The multicenter design of our study and the available long-term follow-up data enhance the generalizability and robustness of our findings. Conscious of unique approaches to tissue-handling protocols in place at the two institutions involved in our digital-pathology workflow, results regarding those analyses have been presented separately, a distinction that provides a robust and nuanced overview of immunological features.

This study also has some limitations, including its retrospective nature and the relatively small sample size of ER-low tumors. Treatment imbalances between the ER-low and ER-neg cohorts might have influenced our clinical outcome analyses and should be considered when interpreting our findings. First, patients with ER-low BC tumors were less frequently exposed to chemotherapy and more frequently managed with surgery upfront compared with ER-neg patients, although post-neoadjuvant tailoring of adjuvant treatment based on the response rate to NACT was not broadly employed in our cohort. Moreover, ET was not frequently administered, reflecting current clinical practice, as oncologists are generally less prone to prescribe ET in ER-low tumors (12,69,70) due to the limited survival benefit reported in earlier studies (2-6) and the notable side effects associated with ET (71). Our study’s limited sample size precludes a definitive evaluation of the impact of these therapeutic decisions on the prognosis of patients with ER-low tumors. In this regard, the numerically worse prognosis we observed in ER-low compared with ER-neg tumors, with an even higher incidence of distant relapses, may support further discussion on the role of ET for selected patients with ER-low tumors (72). Nonetheless, the comparable survival between ER-low and ER-neg tumors seen in our study, consistent with larger cohorts (11,73), underscores the urgent need to generate robust evidence to guide the clinical trajectory of patients with ER-low tumors.

The comparison of TILs in the non-consecutively treated ER-int cohorts warrants caution, due to limited sample size and the potential selection bias.

Potential analytical challenges stemming from the absence of a centralized review of both ER status (74,75) and TIL density cannot be excluded; however, we believe that these issues were mitigated. Tumor samples were evaluated by experienced and dedicated BC pathologists at single pathology units within high-volume comprehensive cancer centers. ER status was locally reviewed, and TILs were quantified on whole-slides following standardized recommended guidelines (37) and using reference images (76). The consistency in TILs distribution of ER-low and ER-neg tumors across our participating institutions further supports our findings and TILs’ established reproducibility (76,77).

The use of SP142 antibody to define PD-L1 positivity in our cohort warrants caution, because this assay has only partial overlap with PD-L1 expression levels defined using 22C3 antibody (78), the antibody used to define pembrolizumab eligibility in the metastatic setting. Still, a cutoff of ≥1% using SP142 has been shown to be predictive of nivolumab benefit in ER-positive patients treated in the Checkmate 7FL trial (35), reinforcing the biological role of evaluating PD-L1 status using SP142 in our cohort.

Moving forward, efforts to personalize cancer treatment in ER-low tumors should focus on examining TME’s functional status and spatial distribution. The use of IHC staining for CD8, FOXP3, and PD-L1 in our cohort allowed us to evaluate key components of the immune compartment using established IHC markers. However, this TME profiling is only partial and may overlook varying immune-states (21), which could affect the efficacy of distinct immunomodulatory combinations across ER statuses. Techniques such as multiplexed single-cell spatially resolved tissue analyses could be instrumental (79) in exploring subtle variations in the immune contexture (80) related to various ER levels, potentially overlooked in our quantitative analysis. Such an approach could pave the way for truly tailored immunotherapy strategies beyond traditional IHC-based classifications, across varying ER levels (32,33).

In conclusion, our results demonstrate that ER-low and ER-neg BC are immunologically and molecularly akin, clarifying their similar clinical outcomes and responses to therapeutics, particularly to ICIs. In this regard, we believe our data contribute notably to the growing body of clinical and translational evidence calling for a reevaluation of ER-based BC classification and management. As such, we advocate for a treatment approach that aligns ER-low tumors with ER-neg, as few guidelines are starting to acknowledge (81), to avoid perpetuating the current disparities in regulatory access to effective treatments for this subgroup of patients. Crucially, this endeavor should encompass at least the inclusion of patients with ER-low and triple-negative tumors in the same clinical trials, a practice already adopted in academic trials (82,83), ensuring that the high-risk ER-low patient population is not deprived from accessing potentially transformative therapies, such as immunotherapy. The evidence in terms of benefit from ICIs, which is stemming from the small subgroups of ER-low patients enrolled in trials dedicated to ER-positive BC, could at the best result in remarkable delay in the access to this treatment option, should long-term survival endpoints support the approval of ICIs in this population.

## Supplementary Material

djae178_Supplementary_Data

## Data Availability

Due to the nature of this research, participants of this study did not give consent for their data to be shared publicly and for secondary use of data derived from the study without Ethics Committee re-evaluation. However, data can be made available upon request through a Data Transfer Agreement and after Ethics Committee approval. We encourage investigators interested in data access to request them by contacting the Department of Surgery, Oncology and Gastroenterology of the University of Padua (ricerca.discog@unipd.it).
