# Supplementary material for: Immune and gene-expression profiling in estrogen receptor low and negative early breast cancer
Source: J Natl Cancer Inst. 2024 Jul 31;116(12):1914–27. doi: 10.1093/jnci/djae178 (PMC11630536; doi:10.1093/jnci/djae178)
Supplement: djae178_Supplementary_Data [file djae178_supplementary_data.zip › djae178_Supplementary_Data/Supplementary Figure 1.pdf]

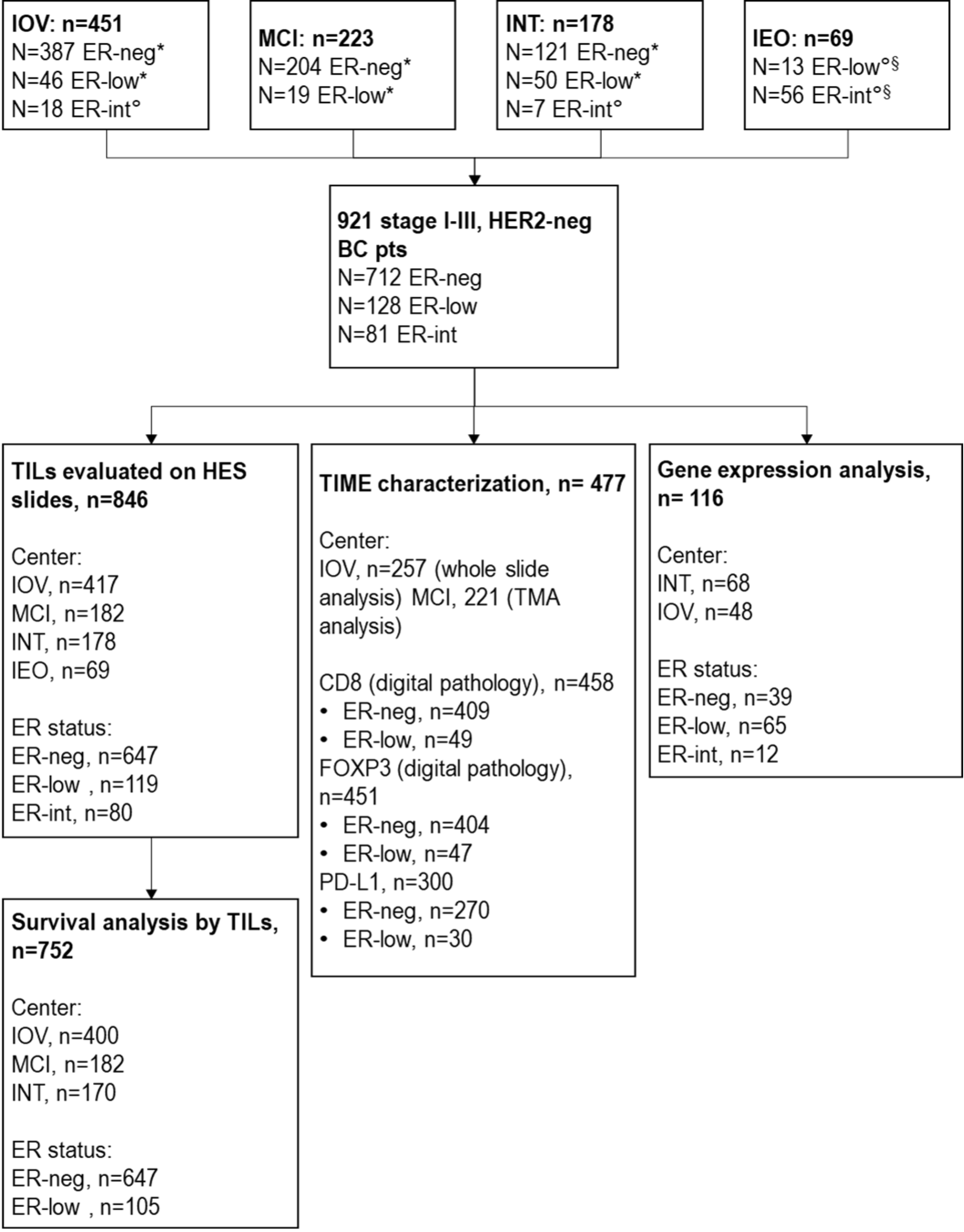

*consecutively-treated patients

°non-consecutively treated patients: excluded from survival analyses

§non-consecutively treated patients identified from an Institutional database of patients who experienced disease relapse: excluded from survival analyses
