# Supplementary material for: Immune and gene-expression profiling in estrogen receptor low and negative early breast cancer
Source: J Natl Cancer Inst. 2024 Jul 31;116(12):1914–27. doi: 10.1093/jnci/djae178 (PMC11630536; doi:10.1093/jnci/djae178)
Supplement: djae178_Supplementary_Data [file djae178_supplementary_data.zip › djae178_Supplementary_Data/Supplementary Material - ERlow - Clean.docx]

**­Supplementary Methods**

**Immunohistochemical protocols for ER, PgR, HER2, CD8, MNF116, FOX3P and PD-L1 staining in place at each Institution Involved in this study: *Istituto Oncologico Veneto (IOV) – IRCCS – Padova; Montpellier Cancer Institute (MCI) – Montpellier; Istituto Europeo di Oncologia (IEO) – IRCCS – Milano; Istituto Nazionale Tumori (INT) – IRCSS – Milano.***

| Institute | Ab | Supplier | Type | Species | Antigen retrieval | Dilution | Incubation time | Rivelation | Platform |
| --- | --- | --- | --- | --- | --- | --- | --- | --- | --- |
| IOV* | ER | Leica Biosystem Novocastra,  Clone 6F11 | monoclonal | mouse | heat-induced | 1:50 | 30 min RT | Novolink™ Polymer Detection Systems. | BOND III (Leica Biosystems) |
|  | PR | Leica Biosystem Novocastra,  Clone 16 | monoclonal | mouse | heat-induced | 1:100 | 30 min RT | Novolink™ Polymer Detection Systems. | BOND III (Leica Biosystems) |
|  | HER2 | Bond Oracle HER2IHC System, Clone CB11 | monoclonal | Mouse | BOND™ Epitope Retrieval ER2 Solution | pre-diluted | 20 min, 100˚C | Refine Detection Kit | BOND III (Leica Biosystems) |
|  | CD8 | Dako Cytomation, C8/144B  Code M7103 | monoclonal | mouse | heat-induced | pre-diluted | 20 min RT | Novolink™ Polymer Detection Systems. | BOND III (Leica Biosystems) |
|  | FOXP3 | Abcam,  236A/E7 | monoclonal | mouse | heat-induced | 1\200 | 20 min RT | Novolink™ Polymer Detection Systems. | BOND III (Leica Biosystems) |
|  | MNF-116 | Dako, clone MNF116  Code M0821 | monoclonal | mouse | heat-induced | 1/50 - 1/100 | 30 min RT | Novolink™ Polymer Detection Systems. | BOND III (Leica Biosystems) |
|  | PD-L1 | Dako Clone 22C3 | monoclonal | rabbit | Low pH | 1/200 | 30 min RT | Flex | Dako Autostainer Link 48 |
| MCI | ER | Leica Clone 6F11  Ref NCL-L-ER-6F11 | monoclonal | mouse | High pH | 1/100 | 20 min RT | Flex | Dako autostainer AS48 |
|  | PR | Dako Clone PgR 636  Ref M3256901-2 | monoclonal | mouse | High pH | 1/400 | 20 min RT | Flex | Dako autostainer AS48 |
|  | HER2 | Dako Herceptest® kit | polyclonal | rabbit | Herceptest® | RTU | 30 min RT | Herceptest® | Dako autostainer AS48 |
|  | CD8 RTU | Dako clone C8/144B Ref IR623 | monoclonal | mouse | High pH | RTU | 20 min RT | Flex | Dako autostainer AS48 |
|  | FoxP3 | AbCam Clone 236A/E7 ab20034 | monoclonal | mouse | Low pH | 1/300 | 20 min RT | Flex + | Dako autostainer AS48 |
|  | PD-L1 | Roche/Spring Clone SP142 | monoclonal | rabbit | High pH | 1/200 | 30 min RT | Flex+ | Dako autostainer AS48 |
| IEO | ER | Dako Clone EP1 | monoclonal | rabbit | High pH | pre-diluted | 10 min RT | Flex HRP | Dako OMNIS |
|  | PR | Dako Clone 1294 | monoclonal | mouse | High pH | pre-diluted | 10 min RT | Flex HRP | Dako OMNIS |
|  | HER2 | Dako Herceptest® kit | polyclonal | rabbit | Herceptest® | RTU | 30 min RT | Herceptest® | Dako Autostainer Link 48 |
| INT | ER | Dako Clone EP1 | monoclonal | rabbit | 15 min High pH | 1:100 | 30 min RT | Flex HRP | Dako Autostainer Link 48 |
|  | HER2 | CEERB-2 | polyclonal | rabbit | 15 min High pH | 1:1500 | 30 min RT | Flex HRP | Dako Autostainer Link 48 |
|  | PR | Dako Clone PgR 636 | monoclonal | mouse | 15 min High pH | 1:200 | 30 min RT | Flex HRP | Dako Autostainer Link 48 |

***** Between 2000 and 2012 IHC staining of formalin-fixed paraffin-embedded (FFPE) tissue was performed using SP1 (790-4325, Ventana Medical System, Tucson AZ, pre-diluted) for ER, 1E2 (790-4296, Ventana Medical System, Tucson AZ, pre-diluted) for PR and 4B5 (790-2991, Ventana Medical System, Tucson AZ, pre-diluted) for HER2. For ER, heat-induced antigen retrieval was done using Cell Conditioning 1 for 36 min and slides were then incubated at 37 °C for 16 min.

**Gene expression analysis**

RNA was extracted from five 5-μm thick sections using the Reliaprep^TM^ RNA cell miniprep system (*Promega*) according to the manufacturer's protocol. Concentration and quality of samples were assessed using the Qubit RNA HS Assay Kit on Qubit 4 Fluorometer (*Thermo Fisher Scientific*) and the High Sensitivity RNA kit on TapeStation 4200 (*Agilent Technologies*), to ensure they had concentration ≥ 20 ng/μL and to evaluate RIN and DV200.

The starting material was 100 to 500 ng of RNA depending on the percentage of fragments with dimensions greater than 200 bp (DV200 value). Samples were hybridized with the Breast Cancer 360^TM^ Panel probes for 19 hours at 65°C at both institutions and then complexes were processed on the nCounter Analysis System (*NanoString Technologies, Inc.*). Cartridges were scanned at 555 FOVs. The Breast Cancer 360 BC360 panel includes comprises 758 target probe pairs genes with 18 additional housekeeping genes used for normalization, 6 exogenous positive control RNA targets that range linearly from 128 fM to 0.125 fM, and 8 exogenous negative control sequences.

**Supplementary Table 1 – Clinicopathological features of all patients included, according to estrogen-receptor (ER)-status: ER-negative (ER-neg, ER <1%), ER-low (ER 1-9%) and ER-intermediate (ER-int, ER 10-50%).**

|  | | ER-neg  (*n*=712) | ER-low  (*n*=128) | ER-int  (*n*=81) | *P* |
| --- | --- | --- | --- | --- | --- |
|  |  | *N(%)* | *N(%)* | *N(%)* |  |
| Institution | IOV | 387 (54.4%) | 46 (35.9%) | 18 (22.2%) | ER-neg vs ER-low:<0.001;  ER-neg vs ER-int:<0.001;  ER-low vs ER-int: 0.001 |
|  | MCI | 204 (28.7%) | 19 (14.8%) | 0 |  |
|  | INT | 121 (17.0%) | 50 (39.1%) | 7 (8.6%) |  |
|  | IEO | 0 | 13 (10.2%) | 56 (69.1%) |  |
| Age, years | Median (IQR) | 54 (45-64) | 53 (44-67) | 48 (40-55) | ER-neg vs ER-low: 1.000;  ER-neg vs ER-int:<0.001;  ER-low vs ER-int:0.001 |
|  | Range | 22-98 | 29-90 | 30-80 |  |
| Histology | Ductal/NOS | 614 (88.1%) | 113 (89.0%) | 63 (77.8%) | ER-neg vs ER-low:0.022;  ER-neg vs ER-int:<0.001;  ER-low vs ER-int: 0.033 |
|  | Lobular | 22 (3.2%) | 11 (8.7%) | 17 (21.0%) |  |
|  | Apocrine | 17 (2.4%) | 0 | 0 |  |
|  | Metaplastic | 9 (1.3%) | 0 | 0 |  |
|  | Medullary | 4 (0.6%) | 0 | 0 |  |
|  | Other | 31 (4.4%) | 3 (2.3%) | 1 (1.2%) |  |
| Grade | 1 | 4 (0.6%) | 0 | 6 (7.9%) | ER-neg vs ER-low:0.243;  ER-neg vs ER-int:<0.001;  ER-low vs ER-int:<0.001 |
|  | 2 | 78 (11.3%) | 20 (16.4%) | 31 (40.8%) |  |
|  | 3 | 607 (88.1%) | 102 (83.6%) | 39 (51.3%) |  |
| PgR | Median (IQR) | 0 (0-0) | 0 (0-1) | 20 (0-40) | ER-neg vs ER-low:<0.001;  ER-neg vs ER-int:<0.001;  ER-low vs ER-int: <0.001 |
|  | Range | 0-5 | 0-9 | 0-95 |  |
| HER2 status | 0 | 459 (64.4%) | 57 (44.9%) | 48 (59.3%) | ER-neg vs ER-low:<0.001;  ER-neg vs ER-int: 0.131;  ER-low vs ER-int: 0.014 |
|  | 1+ | 182 (25.5%) | 55 (43.3%) | 19 (23.5%) |  |
|  | 2+/ISH unamplified | 71 (10.0%) | 15 (11.8%) | 14 (17.3%) |  |
| Ki67 | Median (IQR) | 60 (35-70) | 60 (35-75) | 25(15-35) | ER-neg vs ER-low: 1.000;  ER-neg vs ER-int <0.001;  ER-low vs ER-int <0.001 |
|  | Range | 1-95 | 5-95 | 2-75 |  |
| Stage | I | 212 (29.9%) | 43 (33.9%) | 16 (20.0%) | ER-neg vs ER-low: 0.230;  ER-neg vs ER-int: 0.021;  ER-low vs ER-int: 0.088 |
|  | II | 402 (56.7%) | 62 (48.8%) | 45 (56.3%) |  |
|  | III | 95 (13.4%) | 22 (17.3%) | 19 (23.8%) |  |
| Nodal status | Negative | 386 (60.8%) | 68 (54.8%) | 29 (36.3%) | ER-neg vs ER-low:0.217;  ER-neg vs ER-int:<0.001;  ER-low vs ER-int: 0.009 |
|  | Positive | 249 (39.2%) | 56 (45.2%) | 51 (63.8%) |  |
| Neoadjuvant chemotherapy | No | 411 (57.7%) | 94 (73.4%) | 62 (76.5%) | ER-neg vs ER-low:<0.001;  ER-neg vs ER-int:<0.001;  ER-low vs ER-int: 0.615 |
|  | Yes | 301 (42.3%) | 34 (26.6%) | 19 (23.5%) |  |
| Response to neoadjuvant chemotherapy | Residual disease | 177 (58.8%) | 20 (58.8%) | 17 (94.4%) | ER-neg vs ER-low: 0.998;  ER-neg vs ER-int: 0.003;  ER-low vs ER-int: 0.007 |
|  | pCR | 124 (41.2%) | 14 (41.2%) | 1 (5.6%) |  |
| Adjuvant chemotherapy | No | 280 (39.3%) | 44 (34.4%) | 32 (39.5%) | ER-neg vs ER-low:0.289;  ER-neg vs ER-int: 0.975;  ER-low vs ER-int: 0.452 |
|  | Yes | 432 (60.7%) | 84 (65.6%) | 49 (60.5%) |  |
| Chemotherapy exposure | No | 43 (6.0%) | 17 (13.3%) | 17 (21.0%) | ER-neg vs ER-low:0.003;  ER-neg vs ER-int:<0.001;  ER-low vs ER-int: 0.141 |
|  | Yes | 669 (94.0%) | 111 (86.7%) | 64 (79.0%) |  |
| Endocrine therapy | No | 476 (94.6%) | 71 (67.6%) | 9 (11.5%) | ER-neg vs ER-low:<0.001;  ER-neg vs ER-int:<0.001;  ER-low vs ER-int: <0.001 |
|  | Yes | 27 (5.4%) | 33 (32.4%) | 69 (88.5%) |  |

*Abbreviations: ER-neg, ER-negative; ER-int, ER-intermediate IQR, interquartile-range; NOS, not otherwise specified; ER, estrogen receptor; PgR, progesterone receptor; ISH, In situ hybridization; pCR, pathologic complete response (ypT0/is ypN0);*

**Supplementary Table 2 – Distribution of Relapse-free survival (RFS) events in estrogen-receptor (ER)-negative (ER <1%) and ER-low (ER 1-9%) In the whole observation period and according to a ≥60 months landmark.**

| RFS event Subtype | RFS events | | | | | RFS events  ≥60 months landmark | | |
| --- | --- | --- | --- | --- | --- | --- | --- | --- |
|  | ER-neg (ER <1%)  (n=712) | | ER-low (ER 1-9%)  (n=114) | | ER-neg (ER <1%)  (n=413) | | ER-low (ER 1-9%)  (n=63) | |
|  | *N (%)* | | *N (%)* | | *N (%)* | | *N (%)* | |
| Total Events | | 196 (27.5%) | | 39 (34.2%) | | 29 (7.0%) | | 9 (14.3%) |
| Relapse Events | | 161 (22.6%) | | 29 (25.4%) | | 14 (3.4%) | | 6 (9.5%) |
| Distant Relapses | | 119 (16.7%) | | 23 (20.2%) | | 9 (2.2%) | | 5 (7.9%) |
| Local Relapses | | 42 (5.9%) | | 6 (5.3%) | | 5 (1.2%) | | 1 (1.6%) |
| Deaths Without Relapse | | 35 (4.9%) | | 10 (8.7%) | | 15 (3.6%) | | 3 (5.9%) |

*Abbreviations: ER, estrogen receptor; ER-neg, ER-negative; RFS, relapse-free survival.*

**Supplementary Table 3 – Distribution of TILs in estrogen-receptor (ER)-negative (ER <1%), ER-low (ER 1-9%) and ER-int (ER 10-50%) according to Stage (I, II, III), Grade (G1-G2, G3) and Ki67 (≥20% cut-off)**

|  | | ER-neg  (ER <1%) | | ER-low  (ER 1-9%) | | ER-int  (ER 10-50%) | |
| --- | --- | --- | --- | --- | --- | --- | --- |
|  |  | *Median TILs (IQR)* | *P* | *Median TILs (IQR)* | *P* | *Median TILs (IQR)* | *P* |
| Grade | G1 – G2 | 5 (2-15) | <0.001 | 5 (3-8) | <0.001 | 5 (2-10) | 0.261 |
|  | G3 | 15 (5-30) |  | 19 (7-35) |  | 5 (2-22) |  |
| Ki67 | <20% | 4 (1-9) | 0.002 | 5 (3-8) | 0.022 | 3 (2-5) | 0.009 |
|  | ≥20% | 10 (5-30) |  | 14 (5-30) |  | 7 (3-18) |  |
| Stage | I | 10 (5-30) | 0.193 | 14 (5-35) | 0.516 | 5 (3-14) | 0.704 |
|  | II | 12 (5-30) |  | 18 (7-30) |  | 4 (2-10) |  |
|  | III | 10 (3-24) |  | 10 (5-24) |  | 5 (2-10) |  |

*Abbreviations: ER-neg, ER-negative; ER-int, ER-intermediate IQR, interquartile-range; G, grade; TILs, tumor infiltrating lymphocytes.*

**Supplementary Table 4 –Multivariate cox analyses for relapse-free survival and overall survival in patients with estrogen receptor (ER)-negative (ER-neg, ER <1%) and ER-low (ER 1-9%) breast cancer, factoring ER-status (ER-neg vs ER-low) as a covariate.**

|  | | Relapse-free Survival | | Overall Survival | |
| --- | --- | --- | --- | --- | --- |
|  |  | HR (95%CI) | *P* | HR (95%CI) | *P* |
| Age | (Cont.) | 1.02 (1.01-1.03) | *0.002* | 1.03 (1.02-1.04) | *<0.001* |
| Stage | I | Ref |  | Ref |  |
|  | II | 2.14 (1.51-3.03) | *<0.001* | 1.95 (1.33-2.85) | *<0.001* |
|  | III | 4.31 (2.85 – 6.53) | *<0.001* | 4.00 (2.53-6.32) | *<0.001* |
| Chemotherapy exposure | No | Ref |  | Ref |  |
|  | Yes | 0.47 (0.30-0.73) | *<0.001* | 0.44(0.27-0.70) | *<0.001* |
| TILs | (1% incr) | 0.98 (0.98-0.99) | *<0.001* | 0.99 (0.98-0.99) | *<0.001* |
| Estrogen-receptor (ER)  status | ER-neg  (ER <1%) | Ref | *0.271* | Ref | *0.223* |
|  | ER-low  (ER 1-9%) | 1.22 (0.86-1.74) |  | 1.28 (0.86-1.89) |  |

*Abbreviations: Cont, continuous; Incr, increase; TILs, tumor infiltrating lymphocytes, HR, hazard ratio.*

**Supplementary Table 5 - Genes up-regulated or down-regulated in estrogen-receptor(ER)-low (ER 1-9%) vs ER-negative (ER <1%) by quantitative Significance Analysis of Microarrays (SAM) analysis**

| Gene ID | Score(d) | q-value(%) |
| --- | --- | --- |
| GATA3 | 2.70763171 | 0.00000000 |
| TFF3 | 2.35076058 | 18.59965636 |
| CBLC | 2.25890758 | 18.59965636 |
| TSPAN1 | 2.24340816 | 18.59965636 |
| CXXC5 | 2.08062855 | 22.31958763 |
| CEACAM6 | 2.04355470 | 22.31958763 |
| ESR1 | 2.01270198 | 22.31958763 |
| AGR2 | 2.01080537 | 22.31958763 |
| TBC1D9 | 1.86885733 | 22.31958763 |
| GPR160 | 1.80374868 | 29.36787846 |
| BRCA1 | 1.79055406 | 29.36787846 |
| S100A14 | 1.75764572 | 29.36787846 |
| ALDOA | 1.74073268 | 29.36787846 |
| LFNG | 1.73670925 | 29.36787846 |
| NRCAM | 1.73202219 | 29.36787846 |
| TFF1 | 1.72973483 | 29.36787846 |
| UBB | 1.72237755 | 29.36787846 |
| PPP2R1A | 1.63755719 | 39.05927835 |
| ARNT2 | 1.57256458 | 45.65370197 |
| HLA.B | 1.56044786 | 45.65370197 |
| HOXB3 | 1.52155029 | 62.49484536 |
| BCAS1 | 1.45855809 | 63.77025037 |
| TAP1 | 1.45044687 | 63.77025037 |
| AREG | 1.44738053 | 63.77025037 |
| FOXA1 | 1.41787284 | 66.95876289 |
| BAIAP3 | 1.40605339 | 66.95876289 |
| CAMK2B | 1.34596226 | 76.72358247 |
| SLC39A6 | 1.33883729 | 76.72358247 |
| CCND1 | 1.28612755 | 76.72358247 |
| PKMYT1 | 1.28220854 | 76.72358247 |
| CEACAM5 | 1.26862666 | 76.72358247 |
| FAM214A | 1.26809829 | 76.72358247 |
| CREBBP | 1.26714747 | 76.72358247 |
| ISG15 | 1.25821549 | 76.72358247 |
| ANXA9 | 1.24987509 | 76.72358247 |
| CA12 | 1.24245126 | 76.72358247 |
| PPP2R2C | 1.24013702 | 76.72358247 |
| BMPR1B | 1.21564964 | 76.72358247 |
| OAS3 | 1.19121769 | 76.72358247 |
| PSMC4 | 1.18945565 | 76.72358247 |
| ARID1A | 1.18940041 | 76.72358247 |
| MAPK3 | 1.17330575 | 76.72358247 |
| DTX1 | 1.17067542 | 76.72358247 |
| RB1 | 1.15913622 | 76.72358247 |
| CDKN3 | 1.15160979 | 76.72358247 |
| STAT1 | 1.14587198 | 76.72358247 |
| MUC1 | 1.14252068 | 76.72358247 |
| IFT140 | 1.14240028 | 76.72358247 |
| XRCC3 | 1.13873103 | 76.72358247 |
| TYMP | 1.12906261 | 76.72358247 |
| MLPH | 1.12429496 | 76.72358247 |
| GTF2H2 | 1.11416004 | 76.72358247 |
| MAPK8IP2 | 1.11021742 | 76.72358247 |
| PRKACA | 1.10359000 | 76.72358247 |
| GRB2 | 1.09857712 | 76.72358247 |
| SIDT1 | 1.09411335 | 76.72358247 |
| TLE3 | 1.08669468 | 76.72358247 |
| TBX1 | 1.05483084 | 76.72358247 |
| PRKCA | 1.02490929 | 76.72358247 |
| HLA.A | 1.01553164 | 76.72358247 |
| PYCARD | 1.00890150 | 76.72358247 |
| RARRES3 | 0.97895620 | 76.72358247 |
| IL4R | 0.97584799 | 76.72358247 |
| LAD1 | 0.97472691 | 76.72358247 |
| LIFR | 0.97446292 | 76.72358247 |
| PARP4 | 0.96797804 | 76.72358247 |
| SKP1 | 0.96736319 | 76.72358247 |
| TNFSF10 | 0.96301229 | 76.72358247 |
| AXIN1 | 0.95685973 | 76.72358247 |
| PLA2G4F | 0.94977918 | 76.72358247 |
| HLA.C | 0.94915738 | 76.72358247 |
| TOP2A | 0.94648674 | 76.72358247 |
| POLD1 | 0.94528432 | 76.72358247 |
| DNAJC12 | 0.94482437 | 76.72358247 |
| EGLN2 | 0.94426900 | 76.72358247 |
| DDX39A | 0.94403471 | 76.72358247 |
| HES1 | 0.92594552 | 76.72358247 |
| KIAA0040 | 0.92195993 | 76.72358247 |
| CACNA2D1 | 0.92112379 | 76.72358247 |
| CCNB1 | 0.90683626 | 76.72358247 |
| DTX3 | 0.90442408 | 76.72358247 |
| PSMB7 | 0.89775473 | 76.72358247 |
| CHRNA5 | 0.89653705 | 76.72358247 |
| LAG3 | 0.89525864 | 76.72358247 |
| BAG1 | 0.88821309 | 76.72358247 |
| HDAC6 | 0.87363571 | 76.72358247 |
| GLI3 | 0.86963259 | 76.72358247 |
| EIF3B | 0.86477882 | 76.72358247 |
| PSMB9 | 0.86410751 | 76.72358247 |
| BLVRA | 0.86318339 | 76.72358247 |
| STC1 | 0.86227350 | 76.72358247 |
| TRIP13 | 0.86147564 | 76.72358247 |
| DUSP4 | 0.85458736 | 76.72358247 |
| ACTR3B | 0.84062247 | 76.72358247 |
| CCNA1 | 0.83698098 | 76.72358247 |
| BCL2 | 0.83616985 | 76.72358247 |
| SHC2 | 0.83089767 | 76.72358247 |
| OAZ1 | 0.82666932 | 76.72358247 |
| ERCC1 | 0.82520644 | 76.72358247 |
| BBC3 | 0.82478736 | 76.72358247 |
| MED1 | 0.81569346 | 76.72358247 |
| GDF15 | 0.81485501 | 76.72358247 |
| B3GNT3 | 0.80317524 | 76.72358247 |
| NTRK2 | 0.80155771 | 76.72358247 |
| SOCS1 | 0.79786097 | 76.72358247 |
| TYK2 | 0.79124380 | 76.72358247 |
| PLA2G2A | 0.78619352 | 99.45985072 |
| JAG1 | 0.78247848 | 99.45985072 |
| NFATC1 | 0.77790856 | 99.45985072 |
| ERBB4 | 0.75808320 | 99.45985072 |
| SOX9 | 0.75399351 | 99.45985072 |
| MLLT3 | 0.75031022 | 99.45985072 |
| CLDN7 | 0.74343522 | 99.45985072 |
| SLC44A4 | 0.74220171 | 99.45985072 |
| TAPBP | 0.73726640 | 99.45985072 |
| GPX3 | 0.73715178 | 99.45985072 |
| PGR | 0.73640464 | 99.45985072 |
| FGFR4 | 0.73600580 | 99.45985072 |
| CD276 | 0.73539619 | 99.45985072 |
| CDC6 | 0.73308201 | 99.45985072 |
| HIST1H3H | 0.71907850 | 99.45985072 |
| TGFBR2 | 0.71602349 | 99.45985072 |
| SIX1 | 0.71069247 | 99.45985072 |
| PTTG1 | 0.71068823 | 99.45985072 |
| TAP2 | 0.71033567 | 99.45985072 |
| SIGIRR | 0.70977132 | 99.45985072 |
| FGFR2 | 0.70161441 | 99.45985072 |
| BAX | 0.69859730 | 99.45985072 |
| EYA2 | 0.69763411 | 99.45985072 |
| RAD52 | 0.69283904 | 99.45985072 |
| ITPR1 | 0.69125755 | 99.45985072 |
| NEO1 | 0.68604276 | 99.45985072 |
| BRCA2 | 0.68521436 | 99.45985072 |
| EMCN | 0.68492108 | 99.45985072 |
| BMP4 | 0.68322875 | 99.45985072 |
| GRB7 | 0.67921782 | 99.45985072 |
| LRP2 | 0.67575361 | 99.45985072 |
| E2F5 | 0.67552085 | 99.45985072 |
| MAP3K12 | 0.67016056 | 99.45985072 |
| FHL1 | 0.67013368 | 99.45985072 |
| HK2 | 0.66725738 | 99.45985072 |
| IL6 | 0.66444713 | 99.45985072 |
| PSMB10 | 0.65812820 | 99.45985072 |
| ITGB6 | 0.65801623 | 99.45985072 |
| PARP2 | 0.65560056 | 99.45985072 |
| ZNF205 | 0.64121534 | 99.45985072 |
| HLA.DMA | 0.63974388 | 99.45985072 |
| SHMT2 | 0.63833031 | 99.45985072 |
| MLH1 | 0.63619107 | 99.45985072 |
| HDAC1 | 0.63392009 | 99.45985072 |
| NAT1 | 0.62673185 | 99.45985072 |
| CDC25C | 0.62281834 | 99.45985072 |
| BMPR2 | 0.62184285 | 99.45985072 |
| PDCD1 | 0.61681025 | 99.45985072 |
| E2F1 | 0.61646534 | 99.45985072 |
| MIS18A | 0.60804823 | 99.45985072 |
| TMPRSS4 | 0.60382509 | 99.45985072 |
| SMAD3 | 0.60177765 | 99.45985072 |
| PDCD1LG2 | 0.59344507 | 99.45985072 |
| GUSB | 0.59199307 | 99.45985072 |
| HGF | 0.58677798 | 99.45985072 |
| CDK4 | 0.58275744 | 99.45985072 |
| SMAD5 | 0.58220641 | 99.45985072 |
| PCNA | 0.57976855 | 99.45985072 |
| HIF1A | 0.57834864 | 99.45985072 |
| MYCT1 | 0.57542692 | 99.45985072 |
| PBX3 | 0.57392813 | 99.45985072 |
| FOXM1 | 0.56487750 | 99.45985072 |
| SFN | 0.56400044 | 99.45985072 |
| HSPA2 | 0.56302463 | 99.45985072 |
| NSD3 | 0.55805413 | 99.45985072 |
| CDC25B | 0.55652237 | 99.45985072 |
| CKB | 0.55119803 | 99.45985072 |
| BTG2 | 0.54926030 | 99.45985072 |
| ELOVL2 | 0.54173374 | 99.45985072 |
| BCL2L1 | 0.53799422 | 99.45985072 |
| FGL2 | 0.53711156 | 99.45985072 |
| CDKN1A | 0.53140822 | 99.45985072 |
| COL4A6 | 0.52682565 | 99.45985072 |
| CETN2 | 0.52255826 | 99.45985072 |
| APH1B | 0.52068775 | 99.45985072 |
| CDCA1 | 0.51913746 | 99.45985072 |
| TPSAB1 | 0.51524703 | 99.45985072 |
| HLA.DRA | 0.51503000 | 99.45985072 |
| RAC2 | 0.51482750 | 99.45985072 |
| FNBP1 | 0.50902924 | 99.45985072 |
| COL2A1 | 0.50097904 | 99.45985072 |
| ACTB | 0.49398067 | 99.45985072 |
| EIF4E2 | 0.49167172 | 99.45985072 |
| SMAD1 | 0.48741976 | 99.45985072 |
| PUM1 | 0.48675874 | 99.45985072 |
| CD44 | 0.48356854 | 99.45985072 |
| CD274 | 0.47666430 | 99.45985072 |
| KIF11 | 0.47596406 | 99.45985072 |
| WNT5A | 0.47520174 | 99.45985072 |
| HLA.E | 0.46974756 | 99.45985072 |
| GREM1 | 0.46374586 | 99.45985072 |
| C5orf38 | 0.46148096 | 99.45985072 |
| PIK3R2 | 0.46023947 | 99.45985072 |
| ORC6L | 0.45613287 | 99.45985072 |
| ADCY9 | 0.45597416 | 99.45985072 |
| ALDH1A1 | 0.45142208 | 99.45985072 |
| ESPL1 | 0.44875829 | 99.45985072 |
| NOD2 | 0.43582640 | 99.45985072 |
| FSTL3 | 0.43577281 | 99.45985072 |
| CDH1 | 0.43467895 | 99.45985072 |
| SMURF2 | 0.43016777 | 99.45985072 |
| MTOR | 0.42977656 | 99.45985072 |
| IL1RN | 0.42829550 | 99.45985072 |
| PARP1 | 0.42665414 | 99.45985072 |
| SPDEF | 0.42457518 | 99.45985072 |
| FAM198B | 0.42360256 | 99.45985072 |
| COL6A3 | 0.42089055 | 99.45985072 |
| IDO1 | 0.42012034 | 99.45985072 |
| TGFB1 | 0.41937325 | 99.45985072 |
| CFD | 0.41628038 | 99.45985072 |
| SCUBE2 | 0.41590458 | 99.45985072 |
| GADD45G | 0.41182784 | 99.45985072 |
| TCF4 | 0.41078154 | 99.45985072 |
| BORCS7 | 0.40904669 | 99.45985072 |
| STK11IP | 0.39848914 | 99.45985072 |
| CACNA1D | 0.38592148 | 99.45985072 |
| HEMK1 | 0.37714453 | 99.45985072 |
| PTCH1 | 0.37359876 | 99.45985072 |
| ATAD2 | 0.37304771 | 99.45985072 |
| ZBTB16 | 0.36868620 | 99.45985072 |
| SFRP2 | 0.36336876 | 99.45985072 |
| GRIA3 | 0.36143412 | 99.45985072 |
| MELK | 0.36105261 | 99.45985072 |
| PECAM1 | 0.36016061 | 99.45985072 |
| AXIN2 | 0.35882631 | 99.45985072 |
| EIF4EBP1 | 0.35881464 | 99.45985072 |
| FLI1 | 0.35855885 | 99.45985072 |
| MAD2L1 | 0.35786423 | 99.45985072 |
| MSR1 | 0.35714273 | 99.45985072 |
| PTGDS | 0.35557755 | 99.45985072 |
| LAMB3 | 0.35537702 | 99.45985072 |
| MDM2 | 0.35490125 | 99.45985072 |
| NCAPH2 | 0.35461871 | 99.45985072 |
| DLL1 | 0.35100015 | 99.45985072 |
| PDGFB | 0.35067215 | 99.45985072 |
| ASPN | 0.35062527 | 99.45985072 |
| POLR2A | 0.34883271 | 99.45985072 |
| NUMBL | 0.34834756 | 99.45985072 |
| JAK2 | 0.34823230 | 99.45985072 |
| EP300 | 0.34727934 | 99.45985072 |
| BIRC5 | 0.34577160 | 99.45985072 |
| CDKN1B | 0.34514427 | 99.45985072 |
| AURKA | 0.34400669 | 99.45985072 |
| HIST3H2BB | 0.33907160 | 99.45985072 |
| FGF13 | 0.33854694 | 99.45985072 |
| TP53 | 0.33700131 | 99.45985072 |
| FGF2 | 0.33387946 | 99.45985072 |
| ROCK1 | 0.33339141 | 99.45985072 |
| CDC20 | 0.33263346 | 99.45985072 |
| RORB | 0.33182673 | 99.45985072 |
| IL2RB | 0.33125232 | 99.45985072 |
| CCL8 | 0.32902362 | 99.45985072 |
| MMP11 | 0.32855235 | 99.45985072 |
| CCNA2 | 0.32724000 | 99.45985072 |
| IGF1 | 0.32692150 | 99.45985072 |
| SNAI2 | 0.32632469 | 99.45985072 |
| IL13RA1 | 0.32596981 | 99.45985072 |
| SMAD4 | 0.32451422 | 99.45985072 |
| G6PD | 0.32357089 | 99.45985072 |
| AR | 0.32269210 | 99.45985072 |
| RASAL1 | 0.32131607 | 99.45985072 |
| TBC1D10B | 0.31985677 | 99.45985072 |
| GZMB | 0.31958499 | 99.45985072 |
| SERPINB5 | 0.31873002 | 99.45985072 |
| COL27A1 | 0.31481500 | 99.45985072 |
| DUSP6 | 0.31309459 | 99.45985072 |
| HLA.DPA1 | 0.30201001 | 99.45985072 |
| NR4A3 | 0.29804493 | 99.45985072 |
| NSD1 | 0.29746281 | 99.45985072 |
| PRKAA2 | 0.29654961 | 99.45985072 |
| HELLS | 0.29555999 | 99.45985072 |
| ERBB2 | 0.29218719 | 99.45985072 |
| PLAT | 0.29027370 | 99.45985072 |
| RNF103 | 0.28998728 | 99.45985072 |
| PRKACB | 0.28729248 | 99.45985072 |
| CPA3 | 0.28646625 | 99.45985072 |
| TMPRSS2 | 0.28568163 | 99.45985072 |
| CDKN2C | 0.28151921 | 99.45985072 |
| WEE1 | 0.27688468 | 99.45985072 |
| HLA.DMB | 0.27586864 | 99.45985072 |
| HBB | 0.27490101 | 99.45985072 |
| EIF2AK3 | 0.27395248 | 99.45985072 |
| NRXN3 | 0.26615795 | 99.45985072 |
| CCND2 | 0.26615666 | 99.45985072 |
| SPC25 | 0.26219528 | 99.45985072 |
| CHAD | 0.26164440 | 99.45985072 |
| NETO2 | 0.25981732 | 99.45985072 |
| PTGER3 | 0.25943824 | 99.45985072 |
| ZEB1 | 0.25609442 | 99.45985072 |
| MKI67 | 0.25453175 | 99.45985072 |
| LRRC32 | 0.24853707 | 99.45985072 |
| TCEAL1 | 0.24598227 | 99.45985072 |
| TTK | 0.24487695 | 99.45985072 |
| LEMD1 | 0.24208386 | 99.45985072 |
| PLCB1 | 0.24134966 | 99.45985072 |
| FLRT3 | 0.24104083 | 99.45985072 |
| ACVR1B | 0.23989605 | 99.45985072 |
| TEK | 0.23642231 | 99.45985072 |
| TGFB3 | 0.21790040 | 99.45985072 |
| APOE | 0.21743079 | 99.45985072 |
| ROBO4 | 0.21661263 | 99.45985072 |
| HDAC10 | 0.21036976 | 99.45985072 |
| DDB2 | 0.20675534 | 99.45985072 |
| CACNA2D3 | 0.20125273 | 99.45985072 |
| NPEPPS | 0.20019037 | 99.45985072 |
| PIK3R3 | 0.19940670 | 102.54500806 |
| COMP | 0.19904437 | 102.54500806 |
| IL6R | 0.19627353 | 102.54500806 |
| BMP7 | 0.19430499 | 102.54500806 |
| MRE11 | 0.19269731 | 102.54500806 |
| NR4A1 | 0.18984607 | 102.54500806 |
| FXYD3 | 0.18970298 | 102.54500806 |
| PALMD | 0.18949886 | 102.54500806 |
| HIST1H2BH | 0.18910264 | 102.54500806 |
| ID2 | 0.18839828 | 102.54500806 |
| NOTCH3 | 0.18778411 | 102.54500806 |
| LINC02381 | 0.17841586 | 102.54500806 |
| UBE2C | 0.17453542 | 102.54500806 |
| PRKCB | 0.17449237 | 102.54500806 |
| MS4A2 | 0.17179444 | 102.54500806 |
| HLA.DPB1 | 0.16949651 | 102.54500806 |
| SNAI1 | 0.16834677 | 102.54500806 |
| SKA3 | 0.16833023 | 102.54500806 |
| CD84 | 0.16694672 | 102.54500806 |
| TWIST2 | 0.16524603 | 102.54500806 |
| JAK1 | 0.16377971 | 102.54500806 |
| PIK3CD | 0.15688806 | 102.54500806 |
| MAPK10 | 0.15365308 | 103.32541657 |
| MFNG | 0.15096437 | 103.32541657 |
| HDC | 0.14818398 | 103.32541657 |
| CACNA1H | 0.14653355 | 103.32541657 |
| EPAS1 | 0.13397707 | 103.32541657 |
| CDKN2A | 0.13359673 | 103.32541657 |
| OGN | 0.13265289 | 103.32541657 |
| CACNG6 | 0.12003130 | 103.32541657 |
| PIK3CG | 0.11926505 | 103.32541657 |
| EREG | 0.11900919 | 103.32541657 |
| CSF3R | 0.11520265 | 103.32541657 |
| ST6GALNAC2 | 0.11504260 | 103.32541657 |
| TWIST1 | 0.11385616 | 103.32541657 |
| CCR5 | 0.10779942 | 103.32541657 |
| FANCF | 0.10325178 | 103.32541657 |
| CD36 | 0.10095672 | 103.32541657 |
| CEP55 | 0.09845111 | 103.32541657 |
| IL3RA | 0.09741895 | 105.90118476 |
| CD24 | 0.09118432 | 105.90118476 |
| ADD1 | 0.08993569 | 105.90118476 |
| WNT7B | 0.08531678 | 105.90118476 |
| HOXB13 | 0.08505364 | 105.90118476 |
| IL10RA | 0.08381129 | 105.90118476 |
| PFDN2 | 0.08084960 | 105.90118476 |
| HDAC5 | 0.07853288 | 105.90118476 |
| THBS4 | 0.07836347 | 105.90118476 |
| CLEC14A | 0.07739131 | 105.90118476 |
| EGFR | 0.07722804 | 105.90118476 |
| CENPF | 0.07636417 | 105.90118476 |
| BLM | 0.07448662 | 105.90118476 |
| BCL6B | 0.07253223 | 105.90118476 |
| ETV7 | 0.07089968 | 105.90118476 |
| PALB2 | 0.06646674 | 105.90118476 |
| ABCF1 | 0.06373481 | 105.90118476 |
| PIK3R5 | 0.06273286 | 105.90118476 |
| NFKBIZ | 0.06106847 | 105.90118476 |
| INHBB | 0.05311341 | 105.90118476 |
| RNASE2 | 0.05277068 | 105.90118476 |
| CXCR6 | 0.05216410 | 105.90118476 |
| ISM1 | 0.05195269 | 105.90118476 |
| MEOX2 | 0.05164262 | 105.90118476 |
| MMP14 | 0.04974779 | 105.90118476 |
| ID4 | -1.78231744 | 102.54500806 |
| MARCO | -1.81449307 | 102.54500806 |
| SOX10 | -1.84002966 | 102.54500806 |
| FOXC1 | -2.06378420 | 55.79896907 |
| PLCE1 | -2.11877492 | 55.79896907 |
| PROM1 | -2.52891297 | 0.00000000 |
| EDN1 | -2.58995541 | 0.00000000 |

**Supplementary Table 6 - Genes up-regulated or down-regulated in estrogen-receptor (ER)-low vs ER-neg (analyzing only samples classified as Basal-like according to PAM50) by quantitative Significance Analysis of Microarrays (SAM) analysis.**

| Gene ID | Score(d) | q-value(%) |
| --- | --- | --- |
| HLA.B | 2.26656704 | 48.04860088 |
| TAP1 | 2.20415541 | 48.04860088 |
| GATA3 | 1.85610022 | 48.04860088 |
| STAT1 | 1.82690588 | 48.04860088 |
| UBB | 1.80417022 | 48.04860088 |
| GTF2H2 | 1.75099360 | 48.04860088 |
| LIFR | 1.66425611 | 93.16465805 |
| TAP2 | 1.64166895 | 93.16465805 |
| CBLC | 1.64162745 | 93.16465805 |
| TYMP | 1.59249864 | 93.16465805 |
| PSMB9 | 1.54119350 | 93.16465805 |
| DTX1 | 1.53312344 | 93.16465805 |
| CXXC5 | 1.48736028 | 93.16465805 |
| BRCA1 | 1.47869923 | 93.16465805 |
| TGFBR2 | 1.46841663 | 93.16465805 |
| PARP4 | 1.42612295 | 93.16465805 |
| HLA.A | 1.41353620 | 93.16465805 |
| NTRK2 | 1.41120178 | 93.16465805 |
| PRKCA | 1.39978994 | 93.16465805 |
| HLA.C | 1.39391468 | 93.16465805 |
| FGL2 | 1.38966248 | 93.16465805 |
| FAM214A | 1.38961006 | 93.16465805 |
| LAG3 | 1.38849632 | 93.16465805 |
| PDCD1LG2 | 1.36412392 | 93.16465805 |
| OAS3 | 1.36376260 | 93.16465805 |
| HLA.DRA | 1.35964685 | 93.16465805 |
| GRB2 | 1.34241238 | 93.16465805 |
| CDKN3 | 1.33348305 | 93.16465805 |
| ESR1 | 1.32679983 | 93.16465805 |
| EMCN | 1.28931899 | 93.16465805 |
| PPP2R1A | 1.28447130 | 93.16465805 |
| PRKCB | 1.27805375 | 93.16465805 |
| CHRNA5 | 1.26556200 | 93.16465805 |
| SOCS1 | 1.25598246 | 93.16465805 |
| HLA.DMA | 1.24804636 | 93.16465805 |
| HLA.DPA1 | 1.23748574 | 93.16465805 |
| HLA.DOB | 1.23124284 | 93.16465805 |
| PRKACA | 1.23067357 | 93.16465805 |
| PTGDS | 1.22068821 | 93.16465805 |
| NRCAM | 1.22039256 | 93.16465805 |
| HLA.DPB1 | 1.21449196 | 93.16465805 |
| HLA.E | 1.21215319 | 93.16465805 |
| FNBP1 | 1.20855307 | 93.16465805 |
| GPR160 | 1.20663791 | 93.16465805 |
| RB1 | 1.20517933 | 93.16465805 |
| ARID1A | 1.18587939 | 95.21960175 |
| RAC2 | 1.16607469 | 98.47798830 |
| GPX3 | 1.14180461 | 105.88487973 |
| IDO1 | 1.14034946 | 105.88487973 |
| TAPBP | 1.12968161 | 105.88487973 |
| PSMB10 | 1.10103525 | 106.84632948 |
| JAG1 | 1.06726545 | 106.84632948 |
| TFF3 | 1.06538095 | 106.84632948 |
| PTTG1 | 1.06411539 | 106.84632948 |
| TNFSF10 | 1.06258465 | 106.84632948 |
| ITPR1 | 1.04803927 | 106.84632948 |
| TBC1D9 | 1.03687642 | 106.84632948 |
| HLA.DMB | 1.02676921 | 106.84632948 |
| CD274 | 1.02177874 | 106.84632948 |
| FGF2 | 1.01283207 | 106.84632948 |
| GUSB | 1.01161472 | 106.84632948 |
| FLI1 | 1.00812890 | 106.84632948 |
| DDX39A | 1.00372237 | 106.84632948 |
| ZBTB16 | 1.00057389 | 106.84632948 |
| PSMB7 | 0.99639370 | 106.84632948 |
| AREG | 0.99134145 | 106.84632948 |
| OAZ1 | 0.98985725 | 106.84632948 |
| ISG15 | 0.98428922 | 106.84632948 |
| RARRES3 | 0.96333352 | 106.84632948 |
| HOXB3 | 0.95470381 | 106.84632948 |
| TOP2A | 0.94576067 | 106.84632948 |
| PDCD1 | 0.94538542 | 106.84632948 |
| PSMC4 | 0.92968365 | 106.84632948 |
| BCL2 | 0.92149120 | 106.84632948 |
| CCND2 | 0.91927543 | 106.84632948 |
| CD19 | 0.91186682 | 106.84632948 |
| PTCH1 | 0.90961764 | 106.84632948 |
| TRIP13 | 0.89925265 | 106.84632948 |
| ACTR3B | 0.89593613 | 106.84632948 |
| MSR1 | 0.89016283 | 109.70112599 |
| HGF | 0.88974322 | 109.70112599 |
| PBX3 | 0.87911189 | 109.70112599 |
| IL3RA | 0.87265785 | 109.70112599 |
| RUNX3 | 0.86446482 | 109.70112599 |
| ARNT2 | 0.85967661 | 109.70112599 |
| PYCARD | 0.85899621 | 109.70112599 |
| CD27 | 0.85051179 | 109.70112599 |
| CCNA1 | 0.84420012 | 109.70112599 |
| SIDT1 | 0.84268575 | 109.70112599 |
| NFATC1 | 0.84234940 | 109.70112599 |
| ENPP2 | 0.84167215 | 109.70112599 |
| FHL1 | 0.84071146 | 109.70112599 |
| E2F5 | 0.83744410 | 109.70112599 |
| PIK3CD | 0.83446551 | 109.70112599 |
| ALDH1A1 | 0.82971859 | 109.70112599 |
| TSPAN1 | 0.82514521 | 109.70112599 |
| EIF3B | 0.81896218 | 109.70112599 |
| ATAD2 | 0.81826045 | 109.70112599 |
| JAK3 | 0.81648210 | 109.70112599 |
| PRKACB | 0.81342485 | 109.70112599 |
| JAK2 | 0.80599798 | 109.70112599 |
| PECAM1 | 0.80280559 | 109.70112599 |
| IL2RB | 0.78916368 | 109.70112599 |
| ERCC1 | 0.78582297 | 109.70112599 |
| BRCA2 | 0.77853900 | 110.52796001 |
| MYCT1 | 0.77501987 | 110.52796001 |
| CREBBP | 0.77189344 | 110.52796001 |
| PAX5 | 0.76781577 | 110.52796001 |
| CCL8 | 0.76542300 | 110.52796001 |
| NEO1 | 0.75870062 | 110.52796001 |
| SKP1 | 0.74900926 | 110.52796001 |
| HIST1H3H | 0.74781051 | 110.52796001 |
| CCND1 | 0.74174183 | 110.52796001 |
| TCF4 | 0.73776319 | 110.56853235 |
| ALDOA | 0.73559189 | 110.56853235 |
| IL10RA | 0.72896302 | 110.56853235 |
| HIF1A | 0.72450813 | 110.56853235 |
| FOXM1 | 0.72268102 | 110.56853235 |
| TLE3 | 0.72210943 | 110.56853235 |
| PIK3CG | 0.71503164 | 110.56853235 |
| SNAI2 | 0.71483197 | 110.56853235 |
| POLD1 | 0.71368994 | 110.56853235 |
| NOD2 | 0.70591296 | 111.01209163 |
| TBX1 | 0.69685892 | 111.01209163 |
| KLRK1 | 0.69110873 | 111.01209163 |
| APH1B | 0.69099454 | 111.01209163 |
| IL6 | 0.67712415 | 111.01209163 |
| FOXA1 | 0.67697680 | 111.01209163 |
| TYK2 | 0.67418510 | 111.01209163 |
| CD84 | 0.66747076 | 111.01209163 |
| IL6R | 0.66072547 | 111.01209163 |
| EPAS1 | 0.65526235 | 111.01209163 |
| CDK4 | 0.65520843 | 111.01209163 |
| CD44 | 0.65376345 | 111.01209163 |
| CDKN1A | 0.65295854 | 111.01209163 |
| DTX3 | 0.64688836 | 111.01209163 |
| IL4R | 0.63888926 | 111.01209163 |
| CDC20 | 0.63832495 | 111.01209163 |
| TEK | 0.63602265 | 111.01209163 |
| COL2A1 | 0.62534995 | 111.01209163 |
| S100A14 | 0.61866344 | 111.01209163 |
| IKZF3 | 0.61727917 | 111.01209163 |
| PRF1 | 0.60800504 | 111.01209163 |
| MLH1 | 0.60652046 | 111.01209163 |
| CMKLR1 | 0.60499306 | 111.01209163 |
| FGFR2 | 0.60406852 | 111.01209163 |
| KIAA0040 | 0.60149390 | 111.01209163 |
| CD8A | 0.60024857 | 111.01209163 |
| TP53 | 0.59286912 | 111.01209163 |
| LFNG | 0.59221010 | 111.01209163 |
| CCR1 | 0.58863405 | 111.01209163 |
| BAIAP3 | 0.57997796 | 111.01209163 |
| CDC25C | 0.57745920 | 111.01209163 |
| GZMA | 0.57514128 | 111.01209163 |
| MAPK3 | 0.56611469 | 111.45391146 |
| PIK3R5 | 0.56467021 | 111.45391146 |
| ANXA9 | 0.56454012 | 111.45391146 |
| MAPK8IP2 | 0.55891446 | 111.45391146 |
| MFNG | 0.55775137 | 111.45391146 |
| BTG2 | 0.55433939 | 111.45391146 |
| HDAC1 | 0.54413647 | 111.45391146 |
| MDM2 | 0.54402993 | 111.45391146 |
| DUSP4 | 0.54270767 | 111.45391146 |
| NUMBL | 0.53580171 | 111.45391146 |
| CD163 | 0.53201206 | 111.45391146 |
| CXCL10 | 0.53122560 | 111.45391146 |
| HDAC6 | 0.51747140 | 111.45391146 |
| MAD2L1 | 0.51566608 | 111.45391146 |
| MKI67 | 0.51315208 | 111.45391146 |
| MTOR | 0.51119271 | 111.45391146 |
| CXCR6 | 0.50949749 | 111.45391146 |
| BMP4 | 0.50746185 | 111.45391146 |
| CCR5 | 0.50718011 | 111.45391146 |
| BIRC5 | 0.50683527 | 111.45391146 |
| SPN | 0.50601036 | 111.45391146 |
| GZMB | 0.50136510 | 111.45391146 |
| NR4A3 | 0.49798998 | 111.45391146 |
| CAMK2B | 0.49785715 | 111.45391146 |
| CA12 | 0.49440064 | 111.45391146 |
| EGLN2 | 0.49120540 | 111.45391146 |
| MLLT3 | 0.49057364 | 111.45391146 |
| PLA2G4F | 0.48919400 | 111.45391146 |
| JAK1 | 0.48902677 | 111.45391146 |
| XRCC3 | 0.48537962 | 111.45391146 |
| CDKN2C | 0.48439776 | 111.45391146 |
| ETV7 | 0.48423643 | 111.45391146 |
| CCNA2 | 0.48346322 | 111.45391146 |
| SIGIRR | 0.48288630 | 111.45391146 |
| FGFR4 | 0.48008205 | 111.45391146 |
| ATM | 0.47646610 | 111.45391146 |
| BAX | 0.46577341 | 111.45391146 |
| CCNB1 | 0.46089004 | 111.45391146 |
| CLEC14A | 0.45956000 | 111.45391146 |
| CACNA2D3 | 0.45667030 | 111.45391146 |
| NKG7 | 0.45463923 | 111.45391146 |
| COL4A6 | 0.45197996 | 111.45391146 |
| FOXP3 | 0.45006010 | 111.45391146 |
| CEACAM6 | 0.44953617 | 111.45391146 |
| CACNA2D1 | 0.44731378 | 111.45391146 |
| VIM | 0.44575600 | 111.45391146 |
| KIF11 | 0.44513542 | 111.45391146 |
| ZEB2 | 0.44224008 | 111.45391146 |
| RORB | 0.44015755 | 111.45391146 |
| CPA3 | 0.43992180 | 111.45391146 |
| CXCL9 | 0.43785116 | 111.45391146 |
| CCL2 | 0.43684728 | 111.45391146 |
| PARP2 | 0.43637748 | 111.45391146 |
| CD68 | 0.43458488 | 111.45391146 |
| NFKBIZ | 0.43394629 | 111.45391146 |
| SERPINB5 | 0.43017448 | 111.54907286 |
| SKA3 | -0.02778412 | 109.70112599 |
| TMPRSS2 | -0.02837395 | 109.70112599 |
| FLRT3 | -0.02903366 | 109.70112599 |
| TBC1D10B | -0.03287973 | 109.70112599 |
| MAPK10 | -0.03433736 | 109.70112599 |
| ISM1 | -0.03803655 | 109.70112599 |
| CDCA7L | -0.04155979 | 106.84632948 |
| NPEPPS | -0.04983799 | 106.84632948 |
| TTK | -0.05165476 | 106.84632948 |
| ASPM | -0.05217212 | 106.84632948 |
| MYC | -0.05225327 | 106.84632948 |
| RFC4 | -0.05428301 | 106.84632948 |
| IL1RN | -0.05470396 | 106.84632948 |
| PRKDC | -0.05536868 | 106.84632948 |
| PGR | -0.06265915 | 106.84632948 |
| RBL1 | -0.06324022 | 106.84632948 |
| WNT7B | -0.06881517 | 106.84632948 |
| NEIL3 | -0.07430166 | 106.71034654 |
| TSPAN7 | -0.07772179 | 106.71034654 |
| ERBB4 | -0.07839228 | 106.71034654 |
| HES1 | -0.07871288 | 106.71034654 |
| ITGB6 | -0.08119354 | 106.71034654 |
| NEIL1 | -0.08951800 | 106.71034654 |
| PIK3CA | -0.09116993 | 106.71034654 |
| EIF4EBP1 | -0.09346557 | 106.71034654 |
| PGK1 | -0.09389503 | 106.71034654 |
| OGN | -0.09585974 | 106.71034654 |
| DKK1 | -0.09654659 | 106.71034654 |
| WEE1 | -0.09717590 | 106.71034654 |
| THBS4 | -0.09746236 | 106.71034654 |
| IL2RA | -0.09753842 | 106.71034654 |
| CAV1 | -0.09864832 | 106.71034654 |
| KIF2C | -0.09948681 | 106.71034654 |
| ELK3 | -0.10214362 | 106.71034654 |
| LEMD1 | -0.10546185 | 106.71034654 |
| SOCS3 | -0.10553758 | 106.71034654 |
| DCN | -0.10633328 | 106.71034654 |
| PSAT1 | -0.10747824 | 106.71034654 |
| CCL5 | -0.10767637 | 106.71034654 |
| SHE | -0.11434415 | 106.71034654 |
| RPS6KB1 | -0.11656009 | 106.71034654 |
| INHBB | -0.11707275 | 106.71034654 |
| HEG1 | -0.12037118 | 106.71034654 |
| NDP | -0.12259457 | 106.71034654 |
| SERPINH1 | -0.12699614 | 106.71034654 |
| CCNE2 | -0.12796120 | 106.71034654 |
| PRKAA2 | -0.12851185 | 106.71034654 |
| DPT | -0.12907314 | 106.71034654 |
| POLQ | -0.13099896 | 106.71034654 |
| PDK4 | -0.13479733 | 106.71034654 |
| KDR | -0.13940414 | 106.71034654 |
| WNT5A | -0.14116834 | 106.71034654 |
| BAMBI | -0.14181804 | 106.71034654 |
| SPRY4 | -0.14182638 | 106.71034654 |
| JAM2 | -0.14433755 | 106.71034654 |
| B3GNT3 | -0.14562252 | 106.71034654 |
| SCARA5 | -0.14716379 | 106.71034654 |
| CD8B | -0.15212520 | 106.71034654 |
| SHC2 | -0.15463584 | 106.71034654 |
| TWIST1 | -0.15522629 | 106.71034654 |
| GAS1 | -0.15874462 | 106.71034654 |
| GHR | -0.16057971 | 106.71034654 |
| KIFC1 | -0.16242812 | 106.71034654 |
| PIK3R2 | -0.16310567 | 106.71034654 |
| PAX8 | -0.16337172 | 106.71034654 |
| KIF23 | -0.16632678 | 106.71034654 |
| CXCL12 | -0.16640738 | 106.71034654 |
| WT1 | -0.16866708 | 106.71034654 |
| CHEK2 | -0.17076818 | 106.71034654 |
| MCM3 | -0.17463720 | 106.71034654 |
| ID2 | -0.17616466 | 106.71034654 |
| CEP55 | -0.18413735 | 106.71034654 |
| EGF | -0.18435027 | 106.71034654 |
| PDGFRB | -0.18546268 | 106.71034654 |
| MAPK1 | -0.18646547 | 106.71034654 |
| PPARG | -0.18925171 | 106.71034654 |
| STC1 | -0.19059953 | 106.71034654 |
| DNAJC12 | -0.19237981 | 106.71034654 |
| LAD1 | -0.19339356 | 106.71034654 |
| TMEM45B | -0.19519518 | 106.71034654 |
| IL24 | -0.20052793 | 106.71034654 |
| SPC25 | -0.20077449 | 106.71034654 |
| ITGAV | -0.20543515 | 106.71034654 |
| MIS18A | -0.20739401 | 106.71034654 |
| TIE1 | -0.21001684 | 106.71034654 |
| LEPR | -0.21200473 | 106.71034654 |
| MIA | -0.21364654 | 106.71034654 |
| UBE2C | -0.21396205 | 106.71034654 |
| ASPN | -0.21836833 | 106.71034654 |
| RRM2 | -0.21934937 | 106.71034654 |
| TCEAL1 | -0.22101678 | 106.71034654 |
| PIK3R1 | -0.22601273 | 106.71034654 |
| NOTCH1 | -0.22720530 | 106.71034654 |
| IL12RB2 | -0.22923082 | 106.71034654 |
| THBS1 | -0.23371937 | 106.71034654 |
| MMP11 | -0.24022309 | 106.71034654 |
| PLA2G2A | -0.24123238 | 106.71034654 |
| PDGFRA | -0.24454411 | 106.71034654 |
| NAT1 | -0.24891146 | 106.71034654 |
| NOTCH2 | -0.25069033 | 106.71034654 |
| PDE9A | -0.25074210 | 106.71034654 |
| LINC02381 | -0.25102933 | 106.71034654 |
| PFDN2 | -0.25106404 | 106.71034654 |
| CDKN1C | -0.25202269 | 106.71034654 |
| SLC44A4 | -0.25269915 | 106.71034654 |
| PMS2 | -0.25471999 | 106.71034654 |
| DDR2 | -0.25627806 | 106.71034654 |
| NRDE2 | -0.25727304 | 106.71034654 |
| PTEN | -0.26005015 | 106.71034654 |
| ELOVL2 | -0.26163140 | 106.71034654 |
| CETN2 | -0.26269621 | 106.71034654 |
| ABCA8 | -0.26420501 | 106.71034654 |
| SPDEF | -0.26508732 | 106.71034654 |
| PALB2 | -0.26588872 | 106.71034654 |
| RAD54L | -0.26622307 | 106.71034654 |
| FANCF | -0.26630611 | 106.71034654 |
| HLA.DQB1 | -0.27191776 | 106.71034654 |
| SKP2 | -0.27517256 | 106.71034654 |
| HIST1H2BH | -0.27667897 | 106.71034654 |
| KRT5 | -0.27695653 | 106.71034654 |
| CDH2 | -0.27831346 | 106.71034654 |
| CSF3R | -0.28051697 | 106.71034654 |
| MMP3 | -0.28267068 | 106.71034654 |
| TBP | -0.28357275 | 106.71034654 |
| COL7A1 | -0.28367554 | 106.71034654 |
| IL1B | -0.28401656 | 106.71034654 |
| CDKN2B | -0.28619144 | 106.71034654 |
| TGFB3 | -0.28664033 | 106.71034654 |
| MMRN2 | -0.28718671 | 106.71034654 |
| HLA.DQA1 | -0.29001990 | 106.71034654 |
| SCUBE2 | -0.29054570 | 106.71034654 |
| FLNC | -0.29347612 | 106.71034654 |
| IL13RA1 | -0.29468439 | 106.71034654 |
| SHC4 | -0.29506890 | 106.71034654 |
| CCNE1 | -0.29613319 | 106.71034654 |
| GABRP | -0.30159389 | 106.71034654 |
| HAS1 | -0.30213145 | 106.71034654 |
| FXYD3 | -0.30474613 | 106.71034654 |
| FAM198B | -0.30531656 | 106.71034654 |
| PLCB1 | -0.30764991 | 106.71034654 |
| FLT3 | -0.30847251 | 106.71034654 |
| GRIN1 | -0.31739683 | 106.71034654 |
| BMP6 | -0.31792967 | 106.71034654 |
| CDH3 | -0.31827773 | 106.71034654 |
| F3 | -0.31971849 | 106.71034654 |
| AKT3 | -0.32483291 | 106.71034654 |
| KRT6B | -0.32754558 | 106.71034654 |
| BMPR1A | -0.32805174 | 106.71034654 |
| PRLR | -0.32814551 | 106.71034654 |
| GRB7 | -0.33257096 | 106.71034654 |
| VEGFA | -0.33437478 | 106.71034654 |
| SP1 | -0.33502106 | 106.71034654 |
| SDHA | -0.33554365 | 106.71034654 |
| SOX17 | -0.33604671 | 106.71034654 |
| MUS81 | -0.33672882 | 106.71034654 |
| MMP14 | -0.33744258 | 106.71034654 |
| FOSL1 | -0.33932672 | 106.71034654 |
| IL20RB | -0.33990355 | 106.71034654 |
| DLL4 | -0.34216618 | 106.71034654 |
| KIF14 | -0.34545499 | 106.71034654 |
| JUN | -0.34637319 | 106.71034654 |
| GADD45A | -0.34912671 | 106.71034654 |
| BMP2 | -0.35344427 | 106.71034654 |
| AGTR1 | -0.35486506 | 106.71034654 |
| RPS6KB2 | -0.35492693 | 106.71034654 |
| WNT5B | -0.35655536 | 106.71034654 |
| CXCL5 | -0.36220404 | 106.71034654 |
| TNKS | -0.36359027 | 106.71034654 |
| RPLP0 | -0.36377117 | 106.71034654 |
| CNTFR | -0.36438350 | 106.71034654 |
| ACVR1B | -0.36444066 | 106.71034654 |
| PPP2CB | -0.37038242 | 106.71034654 |
| CACNG6 | -0.37660347 | 106.71034654 |
| CDC14B | -0.38114862 | 106.71034654 |
| ZFPM2 | -0.38306869 | 106.71034654 |
| IL22RA2 | -0.38345658 | 106.71034654 |
| FAP | -0.38498524 | 106.71034654 |
| ADAM12 | -0.38503687 | 106.71034654 |
| CYP4F3 | -0.39812205 | 106.71034654 |
| CACNA1D | -0.40073001 | 106.71034654 |
| PREP | -0.40523794 | 106.71034654 |
| ENO1 | -0.40561975 | 106.71034654 |
| CHIT1 | -0.40971419 | 106.71034654 |
| CCL4 | -0.41125976 | 106.71034654 |
| CLDN3 | -0.41701174 | 106.71034654 |
| SF3A1 | -0.41912507 | 106.71034654 |
| HMGA1 | -0.42048632 | 106.71034654 |
| IRF6 | -0.42131575 | 106.71034654 |
| ECM2 | -0.42178983 | 106.71034654 |
| WNT6 | -0.42308868 | 106.71034654 |
| MYBL2 | -0.44032671 | 106.71034654 |
| TTYH1 | -0.44130933 | 106.71034654 |
| THY1 | -0.44287179 | 106.71034654 |
| TFRC | -0.44291643 | 106.71034654 |
| CCL7 | -0.44613905 | 106.71034654 |
| FAM83D | -0.44793444 | 106.71034654 |
| IRX1 | -0.44805584 | 106.71034654 |
| CD24 | -0.45624445 | 106.71034654 |
| CDK1 | -0.46209662 | 106.71034654 |
| WNT11 | -0.46428258 | 106.71034654 |
| CDKN2D | -0.46450360 | 106.71034654 |
| LEFTY2 | -0.46589091 | 106.71034654 |
| CLEC5A | -0.46601832 | 106.71034654 |
| LEP | -0.46863508 | 106.71034654 |
| ERBB2 | -0.47159391 | 106.71034654 |
| CACNA1H | -0.47495326 | 106.71034654 |
| BMP5 | -0.48330282 | 106.71034654 |
| CCL3L1 | -0.48635283 | 106.71034654 |
| TNF | -0.48857320 | 106.71034654 |
| COMP | -0.48915208 | 106.71034654 |
| HIST1H1C | -0.49329668 | 106.71034654 |
| CKMT1A | -0.49867034 | 106.71034654 |
| ANLN | -0.49908479 | 106.71034654 |
| MMP7 | -0.50040986 | 106.71034654 |
| BBOX1 | -0.50582713 | 106.71034654 |
| HK2 | -0.51020277 | 106.71034654 |
| FUT3 | -0.51218272 | 106.71034654 |
| EIF2AK3 | -0.52236139 | 106.71034654 |
| RNF103 | -0.53139609 | 106.71034654 |
| ATP10B | -0.53634168 | 106.71034654 |
| NUDT1 | -0.54044772 | 106.71034654 |
| VCAN | -0.54183296 | 106.71034654 |
| SFRP4 | -0.54414593 | 106.71034654 |
| ITGA6 | -0.54647416 | 106.71034654 |
| EYA4 | -0.55439791 | 106.71034654 |
| SELE | -0.55717461 | 106.71034654 |
| PIK3R3 | -0.55796483 | 106.71034654 |
| PCK1 | -0.56104922 | 106.71034654 |
| EREG | -0.56292369 | 106.71034654 |
| RAD51C | -0.57435221 | 106.71034654 |
| PRC1 | -0.57475284 | 106.71034654 |
| MCM2 | -0.57576279 | 106.71034654 |
| RASGRF2 | -0.57579203 | 106.71034654 |
| ROCK2 | -0.57845376 | 106.71034654 |
| EGLN3 | -0.58874326 | 106.71034654 |
| CACNG4 | -0.58913392 | 106.71034654 |
| SRPX | -0.58947279 | 106.71034654 |
| BMP7 | -0.59318165 | 106.71034654 |
| NCAM1 | -0.60039100 | 106.71034654 |
| CKS1B | -0.60053769 | 106.71034654 |
| ANGPT1 | -0.60466815 | 106.71034654 |
| FAM124B | -0.60740979 | 106.71034654 |
| SFRP1 | -0.60957645 | 106.71034654 |
| XRCC2 | -0.61470105 | 106.71034654 |
| WIF1 | -0.61577835 | 106.71034654 |
| FZD7 | -0.61812074 | 106.71034654 |
| SLC2A1 | -0.61888305 | 106.71034654 |
| PRKX | -0.62188654 | 106.71034654 |
| MRPL19 | -0.63798846 | 106.71034654 |
| FBN1 | -0.63832090 | 106.71034654 |
| EYA1 | -0.64699390 | 106.71034654 |
| SPP1 | -0.65228266 | 106.71034654 |
| ACVR1C | -0.65492573 | 106.71034654 |
| UBE2T | -0.66456404 | 106.71034654 |
| CHAD | -0.67140560 | 106.71034654 |
| OCLN | -0.67401549 | 106.71034654 |
| MT1G | -0.68029864 | 106.71034654 |
| FZD10 | -0.68401782 | 106.71034654 |
| TGFB2 | -0.68596014 | 106.71034654 |
| NGFR | -0.69038412 | 106.71034654 |
| ETV4 | -0.69283474 | 106.71034654 |
| OLFML2B | -0.69376205 | 106.71034654 |
| IL20RA | -0.69415595 | 106.71034654 |
| S100A7 | -0.69812440 | 106.71034654 |
| HNF1A | -0.70259173 | 106.71034654 |
| GJB2 | -0.70321713 | 106.71034654 |
| TNN | -0.70437378 | 106.71034654 |
| KRT7 | -0.71380402 | 106.71034654 |
| NRXN1 | -0.71396779 | 106.71034654 |
| DEPDC1 | -0.71473413 | 106.71034654 |
| CDCA5 | -0.71559431 | 106.71034654 |
| FGF7 | -0.71776751 | 106.71034654 |
| FGF18 | -0.72244356 | 106.71034654 |
| CACNG1 | -0.73815089 | 106.71034654 |
| PTGS2 | -0.74042935 | 106.71034654 |
| PLAT | -0.74179764 | 106.71034654 |
| FGF10 | -0.74412987 | 106.71034654 |
| JAG2 | -0.74570327 | 106.71034654 |
| CEACAM5 | -0.74772718 | 106.71034654 |
| HDAC2 | -0.75460421 | 106.71034654 |
| MAP2K4 | -0.75467748 | 106.71034654 |
| DSC2 | -0.76592848 | 106.71034654 |
| FOS | -0.76709725 | 106.71034654 |
| MAML2 | -0.77049198 | 106.71034654 |
| GRIN2A | -0.77338448 | 106.71034654 |
| DKK2 | -0.78464261 | 106.71034654 |
| FSTL1 | -0.79030553 | 106.71034654 |
| EXO1 | -0.80239368 | 106.71034654 |
| AGT | -0.80943416 | 106.71034654 |
| INHBA | -0.80993459 | 106.71034654 |
| NUPR1 | -0.81029878 | 106.71034654 |
| PIM1 | -0.81140318 | 106.71034654 |
| TMPRSS4 | -0.81685251 | 106.71034654 |
| JCAD | -0.82025106 | 106.71034654 |
| BDNF | -0.82301138 | 106.71034654 |
| GATA4 | -0.82360446 | 106.71034654 |
| CD1E | -0.82477929 | 106.71034654 |
| SPRY2 | -0.83394475 | 106.71034654 |
| CHI3L1 | -0.83396376 | 106.71034654 |
| FGF1 | -0.83856484 | 106.71034654 |
| MAPT | -0.84724082 | 106.71034654 |
| ZFYVE9 | -0.85051102 | 106.71034654 |
| ADM | -0.85628648 | 106.71034654 |
| RAD51 | -0.85843250 | 106.71034654 |
| PHGDH | -0.85916961 | 106.71034654 |
| RASGRP1 | -0.86135538 | 106.71034654 |
| BCL11A | -0.88478946 | 106.71034654 |
| FGF9 | -0.88648033 | 106.71034654 |
| SPRY1 | -0.88719967 | 106.71034654 |
| FGF12 | -0.88839355 | 106.71034654 |
| LAMC2 | -0.90513898 | 106.71034654 |
| CDC25A | -0.90794365 | 106.71034654 |
| KNTC2 | -0.90925667 | 106.71034654 |
| BAIAP2L1 | -0.90973971 | 106.71034654 |
| IGF1R | -0.91523969 | 106.71034654 |
| CDCA8 | -0.91681528 | 106.71034654 |
| EFNA5 | -0.91925354 | 106.71034654 |
| ITGB1 | -0.92520606 | 106.71034654 |
| BNC2 | -0.92569341 | 106.71034654 |
| WNT4 | -0.93904671 | 106.71034654 |
| EFNA3 | -0.94226429 | 106.71034654 |
| SERBP1 | -0.94379637 | 106.71034654 |
| SOX2 | -0.97394985 | 106.71034654 |
| PPARGC1A | -0.98125827 | 106.71034654 |
| DTX4 | -0.98677642 | 106.71034654 |
| HDAC11 | -0.99430848 | 106.71034654 |
| CRYAB | -1.00547593 | 106.71034654 |
| GSK3B | -1.00641024 | 106.71034654 |
| LPL | -1.02203394 | 106.71034654 |
| FZD9 | -1.02608523 | 106.71034654 |
| LIF | -1.02825075 | 106.71034654 |
| VIT | -1.03086300 | 106.71034654 |
| BAD | -1.03136481 | 106.71034654 |
| DLL3 | -1.05366951 | 106.71034654 |
| THBS2 | -1.05745228 | 106.71034654 |
| KRT17 | -1.05972493 | 106.71034654 |
| TNFAIP6 | -1.05985289 | 106.71034654 |
| ELF3 | -1.07109168 | 106.71034654 |
| ITGB3 | -1.08259660 | 106.71034654 |
| FST | -1.08785157 | 106.71034654 |
| NASP | -1.09232925 | 106.71034654 |
| GNG4 | -1.09440644 | 106.71034654 |
| MET | -1.09473821 | 106.71034654 |
| HOXA9 | -1.09951829 | 106.71034654 |
| SUV39H2 | -1.11549416 | 106.71034654 |
| SYTL4 | -1.15094308 | 106.71034654 |
| HOXA5 | -1.15169699 | 106.71034654 |
| VEGFD | -1.15675472 | 106.71034654 |
| MMP9 | -1.16724261 | 106.71034654 |
| TYMS | -1.16905974 | 106.71034654 |
| GADD45B | -1.18729420 | 106.71034654 |
| KCNB1 | -1.18782312 | 106.71034654 |
| LAMA3 | -1.21721919 | 106.71034654 |
| SLPI | -1.24278875 | 106.71034654 |
| COL9A3 | -1.24349548 | 106.71034654 |
| SMC1B | -1.27917734 | 105.88487973 |
| MARCO | -1.28383230 | 105.88487973 |
| ST6GALNAC2 | -1.29900251 | 105.88487973 |
| NEIL2 | -1.30915437 | 105.88487973 |
| GDF5 | -1.34844805 | 95.21960175 |
| TCF7L1 | -1.38014537 | 64.06480118 |
| CALML5 | -1.38087689 | 64.06480118 |
| SOX10 | -1.38354092 | 64.06480118 |
| ID4 | -1.38790783 | 64.06480118 |
| COL11A1 | -1.41237536 | 64.06480118 |
| COLEC12 | -1.43778546 | 64.06480118 |
| TLX1 | -1.44929750 | 64.06480118 |
| HOXA7 | -1.45213226 | 64.06480118 |
| HAPLN1 | -1.46217202 | 64.06480118 |
| RORA | -1.46553810 | 64.06480118 |
| BMP8A | -1.50030606 | 64.06480118 |
| LTBP1 | -1.51130388 | 64.06480118 |
| CLDN4 | -1.51352336 | 64.06480118 |
| FREM2 | -1.51527710 | 64.06480118 |
| GPC4 | -1.54812701 | 64.06480118 |
| FOXC1 | -1.54884091 | 64.06480118 |
| CXADR | -1.56855447 | 64.06480118 |
| SMO | -1.61874104 | 64.06480118 |
| WNT2 | -1.62306978 | 64.06480118 |
| FGFR3 | -1.71532704 | 28.02835052 |
| CXCL8 | -1.72820030 | 28.02835052 |
| IBSP | -2.01889738 | 28.02835052 |
| PLCE1 | -2.12565068 | 28.02835052 |
| MYCN | -2.17708533 | 28.02835052 |
| EDN1 | -2.52924331 | 0.00000000 |
| PROM1 | -2.55809133 | 0.00000000 |

**Supplementary Table 7 - Genes up-regulated or down-regulated in estrogen-receptor (ER)-low vs ER-intermediate by quantitative Significance Analysis of Microarrays (SAM) analysis.**

| Gene ID | Score(d) | q-value(%) |
| --- | --- | --- |
| DLGAP5 | 3.08642978 | 3.98493259 |
| ABCF1 | 3.01518505 | 3.98493259 |
| PRKCA | 2.61004498 | 3.98493259 |
| TRIP13 | 2.58750706 | 3.98493259 |
| CDC7 | 2.57256666 | 3.98493259 |
| TUBA4A | 2.53035387 | 3.98493259 |
| TAP1 | 2.47714986 | 3.98493259 |
| TOP2A | 2.46858970 | 3.98493259 |
| DSC2 | 2.45491052 | 3.98493259 |
| MELK | 2.41434312 | 3.98493259 |
| E2F5 | 2.38154164 | 3.98493259 |
| ENO1 | 2.37127801 | 3.98493259 |
| MMP9 | 2.34385339 | 3.98493259 |
| CXCL10 | 2.30231922 | 3.98493259 |
| CDKN3 | 2.27183360 | 3.98493259 |
| HLA.DMA | 2.23771654 | 3.98493259 |
| MYBL2 | 2.23065041 | 3.98493259 |
| CDH3 | 2.22391667 | 3.98493259 |
| FAM83D | 2.20198786 | 3.98493259 |
| SKP2 | 2.15168559 | 4.07032401 |
| TAP2 | 2.13276819 | 4.07032401 |
| CDC25C | 2.11841739 | 4.07032401 |
| KIF11 | 2.07011908 | 4.07032401 |
| CXCL13 | 2.04876254 | 4.11337775 |
| E2F1 | 2.01960998 | 4.11337775 |
| LAMC2 | 2.01054942 | 4.11337775 |
| PSMC4 | 1.97244538 | 4.11337775 |
| PGK1 | 1.94877371 | 4.11337775 |
| STAT1 | 1.92686362 | 4.11337775 |
| CHRNA5 | 1.89908040 | 4.25787318 |
| ATAD2 | 1.89706341 | 4.25787318 |
| GZMA | 1.89161503 | 4.25787318 |
| PRKCB | 1.84616650 | 4.25787318 |
| CLDN1 | 1.83675494 | 4.25787318 |
| ORC6L | 1.82926947 | 4.25787318 |
| HLA.E | 1.81579349 | 4.25787318 |
| CCNE2 | 1.81014546 | 4.25787318 |
| HLA.DRA | 1.80941923 | 4.25787318 |
| SPP1 | 1.80061657 | 4.25787318 |
| PDCD1LG2 | 1.79263149 | 4.25787318 |
| KIFC1 | 1.71557287 | 4.62047362 |
| CDC25B | 1.70210881 | 4.62047362 |
| KRT7 | 1.66666009 | 4.62047362 |
| HDAC1 | 1.63703355 | 4.82260611 |
| CXCL8 | 1.63613914 | 4.82260611 |
| EIF4EBP1 | 1.61021693 | 4.82260611 |
| MSR1 | 1.56244651 | 4.82260611 |
| UBE2C | 1.54769409 | 4.99965559 |
| HLA.DOB | 1.54125354 | 4.99965559 |
| CYBB | 1.51461346 | 4.99965559 |
| CD84 | 1.50982850 | 4.99965559 |
| NOTCH2 | 1.49066006 | 4.99965559 |
| CDCA1 | 1.47664595 | 4.99965559 |
| ACTR3B | 1.43948618 | 5.41294008 |
| CENPF | 1.43916392 | 5.41294008 |
| NCAPH2 | 1.40972628 | 5.41294008 |
| BIRC5 | 1.39902295 | 5.41294008 |
| PKMYT1 | 1.39196263 | 5.78103989 |
| PSMB9 | 1.38229145 | 5.78103989 |
| BCL11A | 1.38179851 | 5.78103989 |
| MMP14 | 1.36155566 | 5.78103989 |
| GGH | 1.35588773 | 5.78103989 |
| DKK1 | 1.33316925 | 5.78103989 |
| PSAT1 | 1.30377201 | 6.21500622 |
| PPP2R1A | 1.29673462 | 6.21500622 |
| CHI3L1 | 1.29059362 | 6.21500622 |
| KIF2C | 1.21830246 | 6.83294535 |
| CD163 | 1.19676718 | 6.83294535 |
| RRM2 | 1.18958592 | 6.83294535 |
| CCL5 | 1.18785983 | 6.83294535 |
| S100A7 | 1.15276311 | 6.83294535 |
| GZMB | 1.14500402 | 6.83294535 |
| SMAD4 | 1.13917563 | 7.90713182 |
| TAPBP | 1.12861919 | 7.90713182 |
| PARP4 | 1.12536559 | 7.90713182 |
| TYMS | 1.12475423 | 7.90713182 |
| EIF3B | 1.12436288 | 7.90713182 |
| LAG3 | 1.11589967 | 7.90713182 |
| GABRP | 1.11253302 | 7.90713182 |
| CDC20 | 1.10467796 | 7.90713182 |
| FGL2 | 1.10465231 | 7.90713182 |
| PCNA | 1.09751928 | 7.90713182 |
| TFDP1 | 1.09352567 | 7.90713182 |
| CD24 | 1.09225086 | 7.90713182 |
| JAK3 | 1.08644982 | 7.90713182 |
| SERPINB5 | 1.08510057 | 7.90713182 |
| LAD1 | 1.08017049 | 7.90713182 |
| CD68 | 1.07050629 | 7.90713182 |
| TTK | 1.06372580 | 7.90713182 |
| MKI67 | 1.04199135 | 7.90713182 |
| HLA.B | 1.02002099 | 8.36917336 |
| HDAC6 | 0.99908765 | 8.36917336 |
| LAMB3 | 0.97947078 | 8.89671901 |
| ICAM1 | 0.97689615 | 8.89671901 |
| HIST1H3H | 0.97521418 | 8.89671901 |
| PHGDH | 0.96906644 | 8.89671901 |
| MMP7 | 0.94807733 | 9.20962199 |
| POPDC3 | 0.93473951 | 9.20962199 |
| HLA.DPB1 | 0.91808957 | 9.62424029 |
| PTGDS | 0.89418244 | 9.95166232 |
| TYMP | 0.86917397 | 10.44354590 |
| IDO1 | 0.86202092 | 10.44354590 |
| OAZ1 | 0.85738336 | 10.44354590 |
| HIF1A | 0.83726758 | 10.77965198 |
| PSMB7 | 0.83545093 | 10.77965198 |
| IL6R | 0.82087905 | 11.15938633 |
| ITGB1 | 0.81699162 | 11.15938633 |
| MIA | 0.81183560 | 11.15938633 |
| PLCB4 | 0.79280159 | 11.51202749 |
| FZD8 | -1.15166144 | 4.12176864 |
| SPC25 | -1.15286073 | 4.12176864 |
| RPS6KA5 | -1.16783740 | 4.12176864 |
| ETV4 | -1.16828683 | 4.12176864 |
| ZBTB16 | -1.17370482 | 4.12176864 |
| CAMK2B | -1.17843811 | 4.12176864 |
| ADAM12 | -1.18551835 | 4.12176864 |
| HK2 | -1.18657071 | 4.12176864 |
| TCF4 | -1.18739439 | 4.12176864 |
| PECAM1 | -1.18909773 | 4.12176864 |
| CCND2 | -1.20244102 | 3.31728161 |
| COL27A1 | -1.20453210 | 3.31728161 |
| IL4R | -1.20977760 | 3.31728161 |
| KLRK1 | -1.21916020 | 3.31728161 |
| NUDT1 | -1.21919551 | 3.31728161 |
| PARP1 | -1.22541330 | 3.31728161 |
| CNTFR | -1.22858760 | 3.31728161 |
| FGF2 | -1.22991419 | 3.31728161 |
| GTF2H2 | -1.23794401 | 3.31728161 |
| HOXA5 | -1.25916651 | 3.31728161 |
| BLM | -1.26488183 | 3.31728161 |
| SMO | -1.26532580 | 3.31728161 |
| SNAI2 | -1.26688537 | 3.31728161 |
| MMP3 | -1.27204464 | 3.31728161 |
| PLA2G2A | -1.28112716 | 3.31728161 |
| TNKS | -1.28361451 | 3.31728161 |
| TYK2 | -1.28482378 | 3.31728161 |
| PIK3R5 | -1.28542597 | 3.31728161 |
| INHBA | -1.29622158 | 3.31728161 |
| ERBB2 | -1.29959845 | 3.31728161 |
| AREG | -1.30963944 | 3.31728161 |
| MYCT1 | -1.31207971 | 3.31728161 |
| CXCL12 | -1.31367527 | 3.31728161 |
| NUPR1 | -1.31397137 | 3.31728161 |
| FNBP1 | -1.32918413 | 2.16465474 |
| CDKN2B | -1.33085213 | 2.16465474 |
| HSPA2 | -1.33315076 | 2.16465474 |
| ID1 | -1.33732565 | 2.16465474 |
| OLFML2B | -1.33865214 | 2.16465474 |
| FOXP3 | -1.34810628 | 2.16465474 |
| THBS2 | -1.35218851 | 2.16465474 |
| LFNG | -1.35515580 | 2.16465474 |
| CAV1 | -1.35558799 | 2.16465474 |
| CXCL5 | -1.36232620 | 2.16465474 |
| SPRY2 | -1.36477496 | 2.16465474 |
| FBN1 | -1.36953926 | 2.16465474 |
| TMPRSS2 | -1.37555767 | 2.16465474 |
| TNFSF10 | -1.39907818 | 2.16465474 |
| CDC14A | -1.41545615 | 2.16465474 |
| SP1 | -1.41629969 | 2.16465474 |
| STK11IP | -1.41661151 | 2.16465474 |
| ELK3 | -1.42112986 | 2.16465474 |
| TIMP4 | -1.42772067 | 2.16465474 |
| PAX5 | -1.43229390 | 2.16465474 |
| DTX4 | -1.43264166 | 2.16465474 |
| SDHA | -1.43544223 | 2.16465474 |
| TTYH1 | -1.43574929 | 2.16465474 |
| CFD | -1.44879027 | 2.16465474 |
| PALMD | -1.44922705 | 2.16465474 |
| KAT2B | -1.45189582 | 2.16465474 |
| EIF4E2 | -1.45778224 | 1.51290996 |
| IRF6 | -1.47297211 | 1.51290996 |
| CLEC14A | -1.47372513 | 1.51290996 |
| NEO1 | -1.48445615 | 1.51290996 |
| BCL2L1 | -1.49184100 | 1.51290996 |
| PIK3CA | -1.49536613 | 1.51290996 |
| IL20RB | -1.50109849 | 1.51290996 |
| PDCD1 | -1.50778782 | 1.51290996 |
| CXCR6 | -1.51208796 | 1.51290996 |
| WNT5A | -1.51761480 | 1.51290996 |
| FGFR4 | -1.52194366 | 1.51290996 |
| POLR2A | -1.52350163 | 1.51290996 |
| EP300 | -1.52568216 | 1.51290996 |
| PBX3 | -1.54204942 | 1.51290996 |
| WNT11 | -1.54748826 | 1.51290996 |
| GRB7 | -1.55356453 | 1.51290996 |
| TWIST1 | -1.55369055 | 1.51290996 |
| SMAD5 | -1.56013855 | 1.51290996 |
| WIF1 | -1.56164231 | 1.51290996 |
| PRKX | -1.56338451 | 1.51290996 |
| PARP2 | -1.56803745 | 1.51290996 |
| PRKACB | -1.59089354 | 1.00266046 |
| INHBB | -1.60109763 | 1.00266046 |
| ISM1 | -1.61154912 | 1.00266046 |
| CCNA1 | -1.61855567 | 1.00266046 |
| CDC14B | -1.62320447 | 1.00266046 |
| IL2RA | -1.63221066 | 1.00266046 |
| CCR2 | -1.63420329 | 1.00266046 |
| IGF1 | -1.64136444 | 1.00266046 |
| AGT | -1.64721152 | 1.00266046 |
| MUS81 | -1.65623144 | 1.00266046 |
| PPARG | -1.66638754 | 1.00266046 |
| LEPR | -1.67883142 | 1.00266046 |
| HEG1 | -1.68242748 | 1.00266046 |
| PLCB1 | -1.68882728 | 1.00266046 |
| NPR1 | -1.69197243 | 1.00266046 |
| ARID1A | -1.69386720 | 1.00266046 |
| IL6 | -1.70052692 | 1.00266046 |
| MDM2 | -1.71362808 | 0.72200869 |
| FGF1 | -1.71381391 | 0.72200869 |
| IL1B | -1.71886264 | 0.72200869 |
| CTSW | -1.72089713 | 0.72200869 |
| MAPK1 | -1.72407468 | 0.72200869 |
| CBLC | -1.72628677 | 0.72200869 |
| WNT6 | -1.72677547 | 0.72200869 |
| SNAI1 | -1.73437996 | 0.72200869 |
| NUMBL | -1.73651731 | 0.72200869 |
| LIFR | -1.75944848 | 0.72200869 |
| CDKN1C | -1.76060210 | 0.72200869 |
| RORA | -1.76132087 | 0.72200869 |
| SRPX | -1.77469561 | 0.72200869 |
| THBS4 | -1.78393061 | 0.72200869 |
| SIX1 | -1.78620892 | 0.72200869 |
| ETV7 | -1.79006282 | 0.72200869 |
| RAD52 | -1.79398382 | 0.72200869 |
| FAM214A | -1.80027570 | 0.72200869 |
| EGF | -1.80960333 | 0.72200869 |
| RORB | -1.81464130 | 0.72200869 |
| F3 | -1.81678334 | 0.72200869 |
| ZFYVE9 | -1.81698684 | 0.72200869 |
| CD276 | -1.82691398 | 0.72200869 |
| ROBO4 | -1.83268913 | 0.72200869 |
| HBB | -1.83949661 | 0.72200869 |
| IL1R2 | -1.84123822 | 0.72200869 |
| BCL6B | -1.84657318 | 0.72200869 |
| TBC1D10B | -1.85499655 | 0.38804587 |
| CD34 | -1.86258303 | 0.38804587 |
| WNT2 | -1.86574144 | 0.38804587 |
| ABCA8 | -1.87751965 | 0.38804587 |
| ACVRL1 | -1.89071033 | 0.38804587 |
| FLNC | -1.89303018 | 0.38804587 |
| MAPK3 | -1.91795635 | 0.38804587 |
| EIF2AK3 | -1.91831880 | 0.38804587 |
| COMP | -1.92769259 | 0.38804587 |
| JAM2 | -1.92839344 | 0.38804587 |
| HIST3H2BB | -1.93395324 | 0.38804587 |
| IGF1R | -1.93598976 | 0.38804587 |
| DHRS2 | -1.94726246 | 0.38804587 |
| BNIP3 | -1.95133907 | 0.38804587 |
| SLC2A1 | -1.95452035 | 0.38804587 |
| MAPK8IP2 | -1.97405475 | 0.38804587 |
| SIGIRR | -1.97966777 | 0.38804587 |
| NGFR | -1.98042415 | 0.38804587 |
| FST | -1.98692440 | 0.38804587 |
| CDC25A | -2.00304354 | 0.38804587 |
| CCND1 | -2.00454751 | 0.38804587 |
| PLA1A | -2.01360051 | 0.38804587 |
| FOSL1 | -2.01662028 | 0.38804587 |
| FGF7 | -2.01718379 | 0.38804587 |
| PPP2CB | -2.01789332 | 0.38804587 |
| BAMBI | -2.01933949 | 0.38804587 |
| AXIN1 | -2.02047042 | 0.18668153 |
| NFKBIZ | -2.02677030 | 0.18668153 |
| COLEC12 | -2.03357431 | 0.18668153 |
| MLLT3 | -2.03643301 | 0.18668153 |
| TIE1 | -2.05029088 | 0.18668153 |
| DLL3 | -2.05220679 | 0.18668153 |
| FOXC2 | -2.05471570 | 0.18668153 |
| FGFR3 | -2.05774784 | 0.18668153 |
| OGN | -2.05973509 | 0.18668153 |
| CKMT1A | -2.06619567 | 0.18668153 |
| PLCE1 | -2.06649499 | 0.18668153 |
| LIF | -2.07773762 | 0.18668153 |
| NFATC1 | -2.07870969 | 0.18668153 |
| GZMH | -2.08677120 | 0.18668153 |
| IBSP | -2.08721346 | 0.18668153 |
| DPT | -2.08828686 | 0.18668153 |
| CCL4 | -2.09577507 | 0.18668153 |
| HOXA9 | -2.09862227 | 0.18668153 |
| TSPAN1 | -2.09876956 | 0.18668153 |
| DLL1 | -2.09979361 | 0.18668153 |
| FGF13 | -2.10194470 | 0.18668153 |
| CD8B | -2.11003075 | 0.18668153 |
| SKA3 | -2.11151028 | 0.18668153 |
| RASAL1 | -2.11899794 | 0.18668153 |
| IL13RA1 | -2.12235073 | 0.18668153 |
| FZD9 | -2.12280107 | 0.18668153 |
| GRIA3 | -2.13343602 | 0.18668153 |
| CETN2 | -2.14071374 | 0.18668153 |
| CEACAM6 | -2.14420367 | 0.18668153 |
| PPARGC1A | -2.14616880 | 0.18668153 |
| MUC1 | -2.16353972 | 0.18668153 |
| JAG2 | -2.16562847 | 0.18668153 |
| S1PR1 | -2.16570605 | 0.18668153 |
| WNT7B | -2.16782880 | 0.18668153 |
| COL4A6 | -2.16833664 | 0.18668153 |
| ITGB3 | -2.17209911 | 0.18668153 |
| RARRES3 | -2.17429175 | 0.18668153 |
| JUN | -2.17729595 | 0.18668153 |
| MYCN | -2.17931504 | 0.18668153 |
| PRKAA2 | -2.17977619 | 0.18668153 |
| HOXB3 | -2.18260240 | 0.18668153 |
| SCARA5 | -2.18614507 | 0.18668153 |
| CDH5 | -2.18805699 | 0.18668153 |
| GSK3B | -2.20041197 | 0.18668153 |
| ERCC1 | -2.20228756 | 0.18668153 |
| DDB2 | -2.20438322 | 0.18668153 |
| IFT140 | -2.21887695 | 0.18668153 |
| RPS6KB2 | -2.22746448 | 0.18668153 |
| NEIL1 | -2.22944960 | 0.18668153 |
| NASP | -2.23216238 | 0.18668153 |
| CEACAM5 | -2.23643969 | 0.18668153 |
| KCNB1 | -2.24072585 | 0.18668153 |
| CDKN2D | -2.24966277 | 0.18668153 |
| HDAC10 | -2.25071701 | 0.18668153 |
| SPRY4 | -2.25827919 | 0.18668153 |
| FLRT3 | -2.28417959 | 0.18668153 |
| NPEPPS | -2.29687950 | 0.18668153 |
| VEGFD | -2.29907492 | 0.18668153 |
| IL1RN | -2.30039785 | 0.18668153 |
| EREG | -2.30143689 | 0.18668153 |
| MRPL19 | -2.31650976 | 0.18668153 |
| TWIST2 | -2.32019990 | 0.18668153 |
| GZMM | -2.32900370 | 0.18668153 |
| CHIT1 | -2.33147783 | 0.18668153 |
| IL11RA | -2.33459223 | 0.18668153 |
| BMP5 | -2.33666684 | 0.18668153 |
| BMP8A | -2.34551172 | 0.18668153 |
| ELF3 | -2.35101894 | 0.18668153 |
| SPRY1 | -2.35423832 | 0.18668153 |
| SYTL4 | -2.35739419 | 0.18668153 |
| BAG1 | -2.38149074 | 0.18668153 |
| BORCS7 | -2.38419834 | 0.18668153 |
| TLX1 | -2.39018097 | 0.18668153 |
| CPA3 | -2.39126473 | 0.18668153 |
| CDKN1A | -2.40913244 | 0.18668153 |
| TPSAB1 | -2.42607171 | 0.18668153 |
| GPR160 | -2.43063659 | 0.00000000 |
| APH1B | -2.43860941 | 0.00000000 |
| TNFAIP6 | -2.44246191 | 0.00000000 |
| NSD3 | -2.44477428 | 0.00000000 |
| CXorf36 | -2.45250343 | 0.00000000 |
| PIP | -2.45489111 | 0.00000000 |
| DLL4 | -2.45770870 | 0.00000000 |
| WNT5B | -2.46179314 | 0.00000000 |
| ZEB1 | -2.46971701 | 0.00000000 |
| AR | -2.47302933 | 0.00000000 |
| KIAA0040 | -2.48493874 | 0.00000000 |
| FZD10 | -2.49037666 | 0.00000000 |
| MMRN2 | -2.49262378 | 0.00000000 |
| BCL2 | -2.50019089 | 0.00000000 |
| PYCARD | -2.50454100 | 0.00000000 |
| LRRC32 | -2.50505423 | 0.00000000 |
| ANGPT1 | -2.52386966 | 0.00000000 |
| MAPK10 | -2.52556408 | 0.00000000 |
| MAP3K12 | -2.52730170 | 0.00000000 |
| FLT3 | -2.53004585 | 0.00000000 |
| EGLN2 | -2.53454120 | 0.00000000 |
| CXXC5 | -2.55280364 | 0.00000000 |
| CACNG4 | -2.55439698 | 0.00000000 |
| SOX2 | -2.55518865 | 0.00000000 |
| CACNG1 | -2.56465103 | 0.00000000 |
| TSPAN7 | -2.57299665 | 0.00000000 |
| GADD45A | -2.57410842 | 0.00000000 |
| EDNRB | -2.57560850 | 0.00000000 |
| ECM2 | -2.58185861 | 0.00000000 |
| BMP2 | -2.58212052 | 0.00000000 |
| SFRP4 | -2.59968502 | 0.00000000 |
| BAD | -2.61794332 | 0.00000000 |
| LEF1 | -2.62044290 | 0.00000000 |
| ACVR1B | -2.62560357 | 0.00000000 |
| EDN1 | -2.62733702 | 0.00000000 |
| ATP10B | -2.63198442 | 0.00000000 |
| JCAD | -2.63766252 | 0.00000000 |
| MS4A2 | -2.64215833 | 0.00000000 |
| MLH1 | -2.65531594 | 0.00000000 |
| EYA1 | -2.67163979 | 0.00000000 |
| AGR2 | -2.67291842 | 0.00000000 |
| TGFB3 | -2.68236053 | 0.00000000 |
| MEOX2 | -2.69501202 | 0.00000000 |
| SHC2 | -2.69700705 | 0.00000000 |
| ARNT2 | -2.69732558 | 0.00000000 |
| PAX8 | -2.69763752 | 0.00000000 |
| GRIN2A | -2.71023714 | 0.00000000 |
| TLE3 | -2.71178787 | 0.00000000 |
| LINC02381 | -2.71997468 | 0.00000000 |
| BCAS1 | -2.74216023 | 0.00000000 |
| WT1 | -2.74261018 | 0.00000000 |
| SMAD3 | -2.74310313 | 0.00000000 |
| DUSP4 | -2.76351341 | 0.00000000 |
| LEP | -2.76703819 | 0.00000000 |
| HOXA7 | -2.76909861 | 0.00000000 |
| BNC2 | -2.77132834 | 0.00000000 |
| CHAD | -2.77221430 | 0.00000000 |
| RASGRF2 | -2.79789306 | 0.00000000 |
| OCLN | -2.79906172 | 0.00000000 |
| NR4A1 | -2.80046124 | 0.00000000 |
| PTGER3 | -2.80095522 | 0.00000000 |
| BMPR1A | -2.80455370 | 0.00000000 |
| PLAT | -2.81138067 | 0.00000000 |
| HAPLN1 | -2.81991402 | 0.00000000 |
| GNG4 | -2.82814384 | 0.00000000 |
| IL20RA | -2.83776204 | 0.00000000 |
| MAPT | -2.84276879 | 0.00000000 |
| DTX3 | -2.84781996 | 0.00000000 |
| SHE | -2.86040131 | 0.00000000 |
| FOS | -2.86180755 | 0.00000000 |
| CSF3R | -2.86551887 | 0.00000000 |
| GDF5 | -2.86644747 | 0.00000000 |
| BAIAP3 | -2.86897037 | 0.00000000 |
| BBC3 | -2.87096566 | 0.00000000 |
| RNASE2 | -2.88238909 | 0.00000000 |
| IL22RA2 | -2.89188374 | 0.00000000 |
| NCAM1 | -2.89386305 | 0.00000000 |
| NRXN3 | -2.89392375 | 0.00000000 |
| SOX17 | -2.89438668 | 0.00000000 |
| HDC | -2.90206102 | 0.00000000 |
| LAMA3 | -2.90487752 | 0.00000000 |
| NRXN1 | -2.92135406 | 0.00000000 |
| FAM124B | -2.94471877 | 0.00000000 |
| PALB2 | -2.94744474 | 0.00000000 |
| CACNA1D | -2.95166750 | 0.00000000 |
| FREM2 | -2.96032229 | 0.00000000 |
| ROCK1 | -2.96786033 | 0.00000000 |
| VIT | -2.97137501 | 0.00000000 |
| ID2 | -2.97523755 | 0.00000000 |
| SMAD1 | -3.00636169 | 0.00000000 |
| ACVR1C | -3.01330981 | 0.00000000 |
| RPS6KB1 | -3.03559978 | 0.00000000 |
| GLI3 | -3.03945715 | 0.00000000 |
| DNAJC12 | -3.04167563 | 0.00000000 |
| ZFPM2 | -3.06314536 | 0.00000000 |
| SELE | -3.07257153 | 0.00000000 |
| HDAC11 | -3.07617071 | 0.00000000 |
| ZNF205 | -3.08496745 | 0.00000000 |
| FGF18 | -3.09312424 | 0.00000000 |
| FGF12 | -3.10633423 | 0.00000000 |
| C5orf38 | -3.11294112 | 0.00000000 |
| EYA4 | -3.11739624 | 0.00000000 |
| RAD51C | -3.13072035 | 0.00000000 |
| TFF3 | -3.13626871 | 0.00000000 |
| ST6GALNAC2 | -3.14820650 | 0.00000000 |
| FOXA1 | -3.15171062 | 0.00000000 |
| NR4A3 | -3.15378984 | 0.00000000 |
| HOXB13 | -3.16295216 | 0.00000000 |
| PCK1 | -3.18760858 | 0.00000000 |
| CACNG6 | -3.20592500 | 0.00000000 |
| MLPH | -3.21708669 | 0.00000000 |
| ZIC2 | -3.23291801 | 0.00000000 |
| BMP4 | -3.24035393 | 0.00000000 |
| SIDT1 | -3.26446882 | 0.00000000 |
| AGTR1 | -3.26529240 | 0.00000000 |
| PTGS2 | -3.28813600 | 0.00000000 |
| BMPR1B | -3.30382129 | 0.00000000 |
| CD1E | -3.31774345 | 0.00000000 |
| SOCS2 | -3.31804193 | 0.00000000 |
| DKK2 | -3.32435108 | 0.00000000 |
| SLC44A4 | -3.34081038 | 0.00000000 |
| STC1 | -3.34083463 | 0.00000000 |
| HAS1 | -3.35873694 | 0.00000000 |
| HNF1A | -3.36198774 | 0.00000000 |
| HEMK1 | -3.38448642 | 0.00000000 |
| TNN | -3.38619782 | 0.00000000 |
| NDP | -3.38856222 | 0.00000000 |
| ELOVL2 | -3.41441823 | 0.00000000 |
| NRDE2 | -3.41678086 | 0.00000000 |
| PLA2G4F | -3.42101100 | 0.00000000 |
| PMS2 | -3.43541939 | 0.00000000 |
| GADD45G | -3.45297152 | 0.00000000 |
| SPDEF | -3.47396730 | 0.00000000 |
| FGF10 | -3.52098133 | 0.00000000 |
| NEIL2 | -3.52371817 | 0.00000000 |
| CACNA1H | -3.53618636 | 0.00000000 |
| GADD45B | -3.53667580 | 0.00000000 |
| MAP2K4 | -3.54236323 | 0.00000000 |
| PRLR | -3.55096803 | 0.00000000 |
| FAM198B | -3.55208225 | 0.00000000 |
| IL24 | -3.57799505 | 0.00000000 |
| GATA3 | -3.63469462 | 0.00000000 |
| ADCY9 | -3.63474821 | 0.00000000 |
| WNT4 | -3.65502413 | 0.00000000 |
| BTG2 | -3.66881430 | 0.00000000 |
| BDNF | -3.67540165 | 0.00000000 |
| PLA2G3 | -3.69428571 | 0.00000000 |
| GRIN1 | -3.70286781 | 0.00000000 |
| CREBBP | -3.71045664 | 0.00000000 |
| GDF15 | -3.71462436 | 0.00000000 |
| GHR | -3.75228611 | 0.00000000 |
| RNF103 | -3.82326154 | 0.00000000 |
| PGR | -3.83820943 | 0.00000000 |
| SLC39A6 | -3.84873002 | 0.00000000 |
| ERBB4 | -3.88962662 | 0.00000000 |
| HES1 | -3.90872127 | 0.00000000 |
| FGF9 | -4.05683056 | 0.00000000 |
| LRP2 | -4.11100713 | 0.00000000 |
| ANXA9 | -4.12814897 | 0.00000000 |
| CA12 | -4.15636906 | 0.00000000 |
| GATA4 | -4.18675952 | 0.00000000 |
| LEFTY2 | -4.19080184 | 0.00000000 |
| SCUBE2 | -4.30270993 | 0.00000000 |
| CDCA8 | -4.32095015 | 0.00000000 |
| CYP4F3 | -4.32579360 | 0.00000000 |
| TBC1D9 | -4.39526967 | 0.00000000 |
| TFF1 | -4.49543538 | 0.00000000 |
| NAT1 | -4.60539349 | 0.00000000 |
| GPC4 | -4.80588245 | 0.00000000 |
| ESR1 | -4.84324229 | 0.00000000 |
| TCEAL1 | -5.07344344 | 0.00000000 |

**Supplementary Table 8 – Immune genes evaluated and functional annotation.**

| Gene | Cell Type | Antigen Presentation | Cytokine and Chemokine Signaling | Immune Infiltration | TGF-beta |
| --- | --- | --- | --- | --- | --- |
| ACVR1B |  | - | - | - | + |
| ACVR1C |  | - | - | - | + |
| ACVRL1 |  | - | - | - | + |
| APOE |  | - | - | + | - |
| BAMBI |  | - | - | - | + |
| BMP2 |  | - | - | - | + |
| BMP4 |  | - | - | - | + |
| BMP5 |  | - | - | - | + |
| BMP6 |  | - | - | - | + |
| BMP7 |  | - | - | - | + |
| BMP8A |  | - | - | - | + |
| BMPR1A |  | - | - | - | + |
| BMPR1B |  | - | - | - | + |
| BMPR2 |  | - | - | - | + |
| CCL2 |  | - | + | - | - |
| CCL21 |  | - | + | - | - |
| CCL3L1 |  | - | + | - | - |
| CCL4 |  | - | + | - | - |
| CCL5 |  | - | + | + | - |
| CCL7 |  | - | + | - | - |
| CCL8 |  | - | + | - | - |
| CCR1 |  | - | + | - | - |
| CCR2 |  | - | + | - | - |
| CCR5 |  | - | + | - | - |
| CD163 | Macrophages | - | - | - | - |
| CD19 | B-cells | - | - | - | - |
| CD1E |  | + | - | - | - |
| CD27 |  | - | - | + | - |
| CD274 |  | - | - | + | - |
| CD276 |  | - | - | + | - |
| CD68 | Macrophages | - | - | - | - |
| CD84 | Macrophages | - | - | - | - |
| CD8A | CD8 T cells | + | - | + | - |
| CD8B | CD8 T cells | - | - | - | - |
| CDKN2B |  | - | - | - | + |
| CHIT1 |  | - | - | + | - |
| CLEC5A |  | - | - | + | - |
| CMKLR1 |  | - | - | + | - |
| COLEC12 |  | - | - | + | - |
| COMP |  | - | - | - | + |
| CPA3 | Mast cells | - | - | - | - |
| CREBBP |  | - | - | - | + |
| CSF3R | Neutrophils | - | + | - | - |
| CTSW | Cytotoxic cells | - | - | - | - |
| CXCL10 |  | - | + | - | - |
| CXCL12 |  | - | + | - | - |
| CXCL13 |  | - | + | - | - |
| CXCL5 |  | - | + | + | - |
| CXCL8 |  | - | + | - | - |
| CXCL9 |  | - | + | + | - |
| CXCR6 |  | - | + | + | - |
| CYBB |  | - | - | + | - |
| DCN |  | - | - | - | + |
| E2F5 |  | - | - | - | + |
| EP300 |  | - | - | - | + |
| FOXP3 | Treg | - | + | - | - |
| FST |  | - | - | - | + |
| GDF5 |  | - | - | - | + |
| GNLY | Cytotoxic cells | - | - | - | - |
| GZMA | Cytotoxic cells | - | - | + | - |
| GZMB | Cytotoxic cells | - | - | + | - |
| GZMH | Cytotoxic cells | - | - | + | - |
| GZMM |  | - | - | + | - |
| HDC | Mast cells | - | - | - | - |
| HLA-A |  | + | - | - | - |
| HLA-B |  | + | - | - | - |
| HLA-C |  | + | - | - | - |
| HLA-DMA |  | + | - | - | - |
| HLA-DMB |  | + | - | - | - |
| HLA-DOB |  | + | + | - | - |
| HLA-DPA1 |  | + | - | - | - |
| HLA-DPB1 |  | + | - | - | - |
| HLA-DQA1 |  | + | - | + | - |
| HLA-DQB1 |  | + | - | - | - |
| HLA-DRA |  | + | - | - | - |
| HLA-DRB1 |  | + | - | + | - |
| HLA-E |  | - | - | + | - |
| ID1 |  | - | - | - | + |
| ID2 |  | - | - | - | + |
| ID4 |  | - | - | - | + |
| IDO1 |  | - | + | + | - |
| IL10RA |  | - | + | - | - |
| IL11RA |  | - | + | - | - |
| IL12RB2 |  | - | + | - | - |
| IL13RA1 |  | - | + | - | - |
| IL1B |  | - | + | - | - |
| IL1R2 |  | - | + | - | - |
| IL1RN |  | - | + | - | - |
| IL20RA |  | - | + | - | - |
| IL20RB |  | - | + | - | - |
| IL22RA2 |  | - | + | - | - |
| IL24 |  | - | + | - | - |
| IL2RA |  | - | + | - | - |
| IL2RB |  | - | + | - | - |
| IL3RA |  | - | + | - | - |
| IL4R |  | - | + | - | - |
| IL6 |  | - | + | - | - |
| IL6R |  | - | + | - | - |
| IL7R |  | - | + | - | - |
| INHBA |  | - | - | - | + |
| INHBB |  | - | - | - | + |
| JAK1 |  | - | + | - | - |
| JAK2 |  | - | + | - | - |
| JAK3 |  | - | + | - | - |
| KLRK1 | Cytotoxic cells | - | - | - | - |
| LAG3 | Exhausted CD8 | - | - | + | - |
| LEFTY2 |  | - | - | - | + |
| LTB |  | - | + | - | - |
| LTBP1 |  | - | - | - | + |
| MAPK1 |  | - | - | - | + |
| MAPK3 |  | - | - | - | + |
| MARCO |  | - | - | + | - |
| MS4A2 | Mast cells | - | - | - | - |
| MSR1 |  | - | - | + | - |
| MYC |  | - | - | - | + |
| NKG7 | Cytotoxic cells | - | - | + | - |
| NOD2 |  | - | + | - | - |
| OAS3 |  | - | + | - | - |
| PDCD1 |  | - | - | + | - |
| PDCD1LG2 |  | - | - | + | - |
| PPP2CB |  | - | - | - | + |
| PPP2R1A |  | - | - | - | + |
| PRF1 | Cytotoxic cells | - | - | - | - |
| PSMB10 |  | + | - | + | - |
| PSMB7 |  | + | - | - | - |
| PSMB9 |  | + | - | - | - |
| PTGS2 |  | - | + | - | - |
| RBL1 |  | - | - | - | + |
| RBL2 |  | - | - | - | + |
| RBX1 |  | - | - | - | + |
| ROCK1 |  | - | - | - | + |
| ROCK2 |  | - | - | - | + |
| RPS6KB1 |  | - | - | - | + |
| RPS6KB2 |  | - | - | - | + |
| SIGIRR |  | - | - | + | - |
| SKP1 |  | - | - | - | + |
| SMAD1 |  | - | - | - | + |
| SMAD3 |  | - | - | - | + |
| SMAD4 |  | - | - | - | + |
| SMAD5 |  | - | - | - | + |
| SMURF2 |  | - | - | - | + |
| SP1 |  | - | - | - | + |
| SPN |  | - | - | + | - |
| SPP1 |  | - | + | - | - |
| STAT1 |  | - | - | + | - |
| TAP1 |  | + | - | - | - |
| TAP2 |  | + | - | - | - |
| TAPBP |  | + | - | - | - |
| TFDP1 |  | - | - | - | + |
| TGFB1 |  | - | - | - | + |
| TGFB2 |  | - | - | - | + |
| TGFB3 |  | - | - | - | + |
| TGFBR2 |  | - | - | - | + |
| THBS1 |  | + | - | - | + |
| THBS2 |  | - | - | - | + |
| THBS4 |  | - | - | - | + |
| TIGIT |  | - | - | + | - |
| TNF |  | - | - | - | + |
| TNFSF10 |  | - | + | - | - |
| TPSAB1 | Mast Cells | - | - | - | - |
| TYK2 |  | - | + | - | - |
| VEGFA |  | - | + | - | - |
| ZFYVE9 |  | - | - | - | + |
| ZNF205 |  | - | - | + | - |

**Supplementary Table 9 - Immune genes significantly differentially expressed between estrogen-receptor (ER)-low (ER 1-9%) and ER-intermediate (ER 10-50%) samples by quantitative Significance Analysis of Microarrays (SAM) analysis with False Discovery Rate <5%.**

| Gene Name | Score (d) | q-value (%) |
| --- | --- | --- |
| TAP1 | 2.47714986 | 3.98493259 |
| E2F5 | 2.38154164 | 3.98493259 |
| CXCL10 | 2.30231922 | 3.98493259 |
| TAP2 | 2.13276819 | 4.07032401 |
| CXCL13 | 2.04876254 | 4.11337775 |
| STAT1 | 1.92686362 | 4.11337775 |
| GZMA | 1.89161503 | 4.25787318 |
| SPP1 | 1.80061657 | 4.25787318 |
| PDCD1LG2 | 1.79263149 | 4.25787318 |
| CXCL8 | 1.63613914 | 4.82260611 |
| MSR1 | 1.56244651 | 4.82260611 |
| CYBB | 1.51461346 | 4.99965559 |
| CD84 | 1.50982850 | 4.99965559 |
| IL4R | -1.20977760 | 3.31728161 |
| KLRK1 | -1.21916020 | 3.31728161 |
| TYK2 | -1.28482378 | 3.31728161 |
| INHBA | -1.29622158 | 3.31728161 |
| CXCL12 | -1.31367527 | 3.31728161 |
| CDKN2B | -1.33085213 | 2.16465474 |
| ID1 | -1.33732565 | 2.16465474 |
| FOXP3 | -1.34810629 | 2.16465474 |
| THBS2 | -1.35218852 | 2.16465474 |
| CXCL5 | -1.36232620 | 2.16465474 |
| TNFSF10 | -1.39907818 | 2.16465474 |
| SP1 | -1.41629969 | 2.16465474 |
| IL20RB | -1.50109849 | 1.51290996 |
| PDCD1 | -1.50778782 | 1.51290996 |
| CXCR6 | -1.51208796 | 1.51290996 |
| EP300 | -1.52568216 | 1.51290996 |
| SMAD5 | -1.56013855 | 1.51290996 |
| INHBB | -1.60109763 | 1.00266046 |
| IL2RA | -1.63221066 | 1.00266046 |
| CCR2 | -1.63420329 | 1.00266046 |
| IL6 | -1.70052692 | 1.00266046 |
| IL1B | -1.71886264 | 0.72200869 |
| CTSW | -1.72089713 | 0.72200869 |
| MAPK1 | -1.72407468 | 0.72200869 |
| THBS4 | -1.78393061 | 0.72200869 |
| ZFYVE9 | -1.81698684 | 0.72200869 |
| CD276 | -1.82691398 | 0.72200869 |
| IL1R2 | -1.84123822 | 0.72200869 |
| ACVRL1 | -1.89071033 | 0.38804587 |
| MAPK3 | -1.91795635 | 0.38804587 |
| COMP | -1.92769259 | 0.38804587 |
| SIGIRR | -1.97966777 | 0.38804587 |
| FST | -1.98692440 | 0.38804587 |
| PPP2CB | -2.01789332 | 0.38804587 |
| BAMBI | -2.01933949 | 0.38804587 |
| COLEC12 | -2.03357431 | 0.18668153 |
| GZMH | -2.08677120 | 0.18668153 |
| CCL4 | -2.09577507 | 0.18668153 |
| CD8B | -2.11003075 | 0.18668153 |
| IL13RA1 | -2.12235074 | 0.18668153 |
| RPS6KB2 | -2.22746448 | 0.18668153 |
| IL1RN | -2.30039785 | 0.18668153 |
| GZMM | -2.32900370 | 0.18668153 |
| CHIT1 | -2.33147783 | 0.18668153 |
| IL11RA | -2.33459223 | 0.18668153 |
| BMP5 | -2.33666684 | 0.18668153 |
| BMP8A | -2.34551172 | 0.18668153 |
| CPA3 | -2.39126473 | 0.18668153 |
| TPSAB1 | -2.42607172 | 0.18668153 |
| BMP2 | -2.58212052 | 0.00000000 |
| ACVR1B | -2.62560357 | 0.00000000 |
| MS4A2 | -2.64215833 | 0.00000000 |
| TGFB3 | -2.68236053 | 0.00000000 |
| SMAD3 | -2.74310313 | 0.00000000 |
| BMPR1A | -2.80455370 | 0.00000000 |
| IL20RA | -2.83776204 | 0.00000000 |
| CSF3R | -2.86551887 | 0.00000000 |
| GDF5 | -2.86644747 | 0.00000000 |
| IL22RA2 | -2.89188374 | 0.00000000 |
| HDC | -2.90206102 | 0.00000000 |
| ROCK1 | -2.96786033 | 0.00000000 |
| ID2 | -2.97523755 | 0.00000000 |
| SMAD1 | -3.00636169 | 0.00000000 |
| ACVR1C | -3.01330981 | 0.00000000 |
| RPS6KB1 | -3.03559978 | 0.00000000 |
| ZNF205 | -3.08496745 | 0.00000000 |
| BMP4 | -3.24035393 | 0.00000000 |
| PTGS2 | -3.28813600 | 0.00000000 |
| BMPR1B | -3.30382129 | 0.00000000 |
| CD1E | -3.31774345 | 0.00000000 |
| IL24 | -3.57799505 | 0.00000000 |
| CREBBP | -3.71045664 | 0.00000000 |
| LEFTY2 | -4.19080184 | 0.00000000 |

**Supplementary Figures**

**Supplementary Figure 1: *Study cohorts***


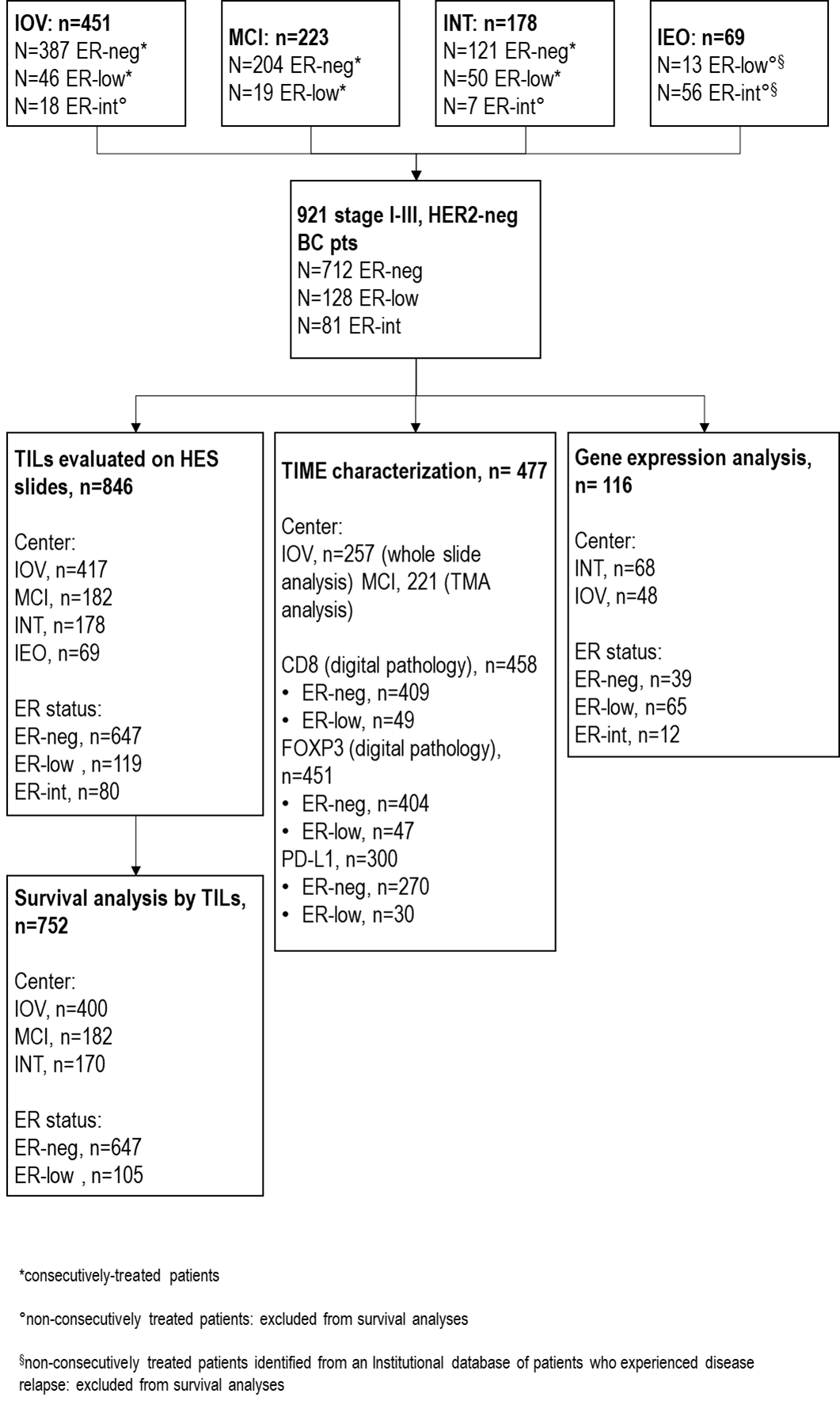


**Supplementary Figure 2 - Kaplan-Meier curves for relapse-free survival (Panel A) and overall survival (Panel B) in patients with estrogen-receptor (ER)-negative (ER<1%) and ER-low (ER 1-9%) tumors. Panel C and D include a landmark-analysis at 60-months for relapse-free survival (C) and overall survival (D).**

**Supplementary Figure 3 - Kaplan-Meier curves comparing relapse-free survival (Panel A) and overall survival (Panel B) in patients with estrogen-receptor (ER)-negative (ER<1%) and ER-low (ER 1-9%) tumors exposed to chemotherapy.**

**Supplementary Figure 4 –TILs distribution measured as a continuous variable (1% increase) in ER-negative (ER<1%) and ER-low (ER 1-9%) tumors according to each Institution involved: Istituto Oncologico Veneto Padova, Italy (p=0.201) (A); Montpellier Cancer Institute [MCI], Montpellier, France (p=0.234) (B); Istituto Nazionale Tumori [INT], Milano, Italy (p=0.347) (C).**

**Supplementary Figure 5- TILs distribution at ≥30% cut-off, in ER-negative (ER<1%) and ER-low (ER 1-9%) tumors according to each Institution involved, Istituto Oncologico Veneto [IOV], Padova, Italy (A); Montpellier Cancer Institute [MCI], Montpellier, France (B); Istituto Nazionale Tumori [INT], Milano, Italy (C).**

*Abbreviations: ER, estrogen receptor; ER-neg, ER-negative (ER <1%), ER-low (ER 1-9%); TILs, tumor infiltrating lymphocytes; n.s., non-significant p-value.*

**Supplementary Figure 6 – Distribution of *tumor-infiltrating lymphocytes* (TILs) as a continuous variable (1% increase), stratified by estrogen receptor (ER) status: ER <1%, ER 1-9%, ER 10-30% and ER 30-50%.**

*Abbreviations: ER, estrogen receptor; ER-neg, ER-negative (ER <1%), ER-low (ER 1-9%); TILs, tumor infiltrating lymphocytes; **,* p*<0.001; n.s., non-significant p value*
