# Supplementary figures and images for: Immune and gene-expression profiling in estrogen receptor low and negative early breast cancer
Source: J Natl Cancer Inst. 2024 Jul 31;116(12):1914–27. doi: 10.1093/jnci/djae178 (PMC11630536; doi:10.1093/jnci/djae178)

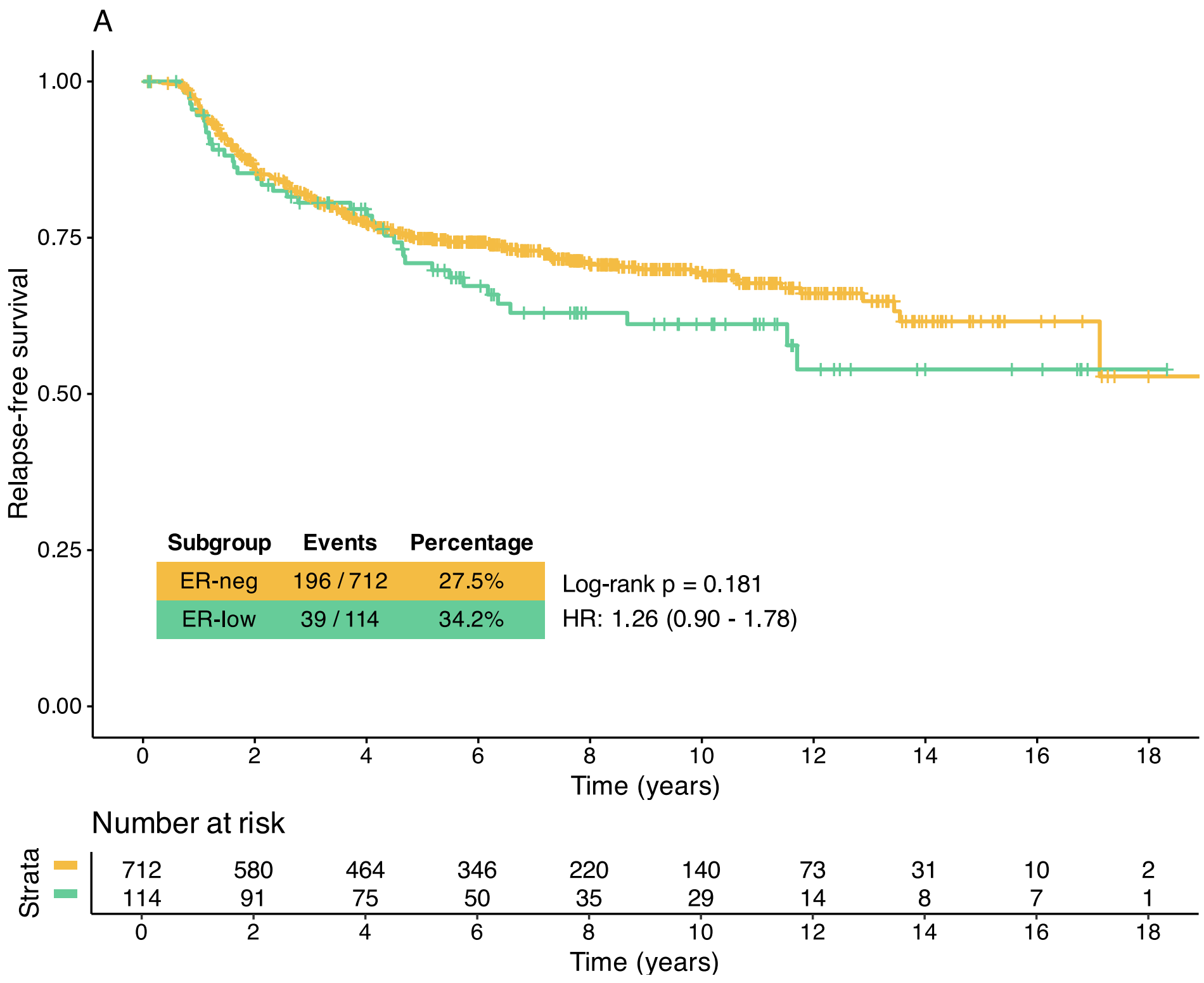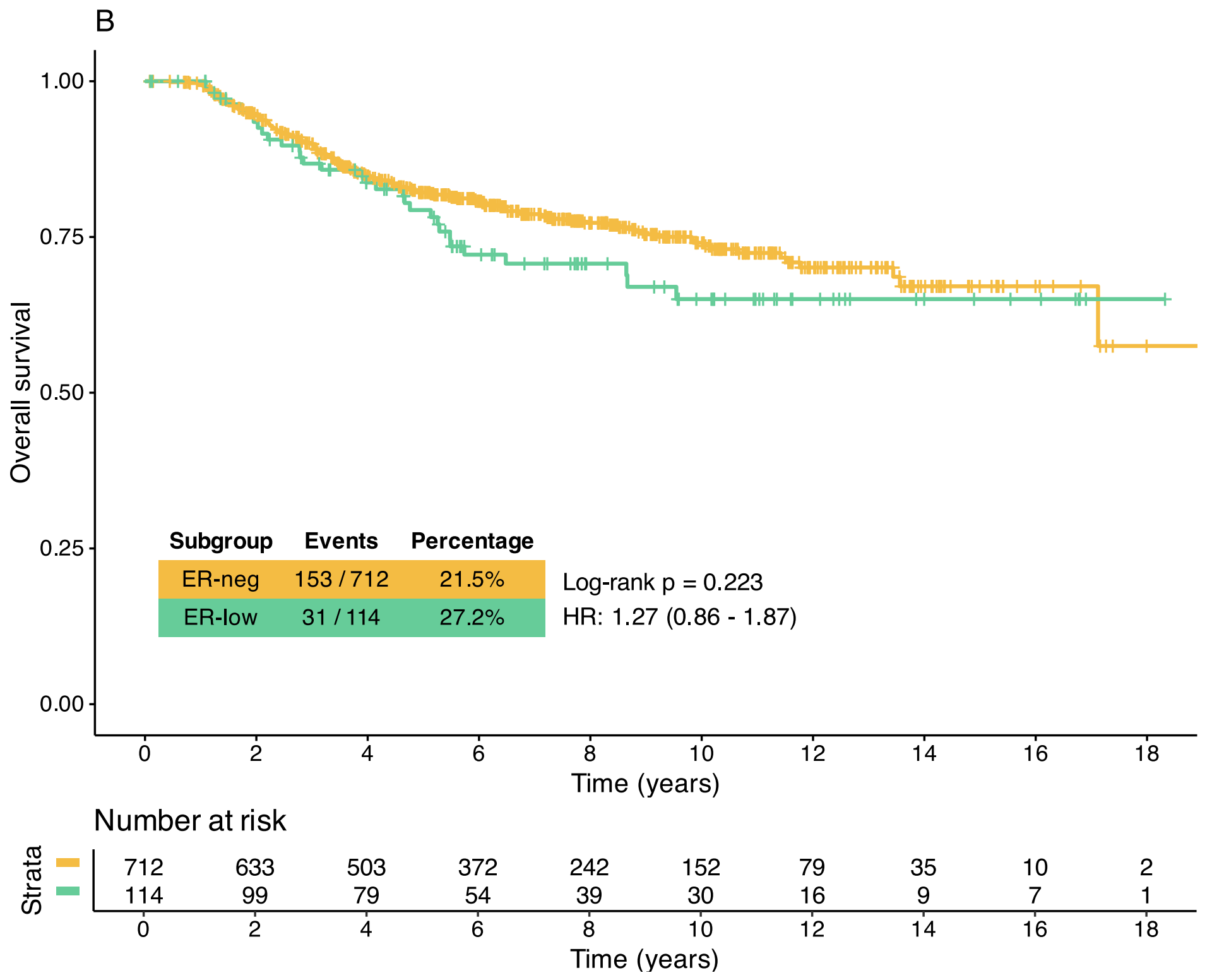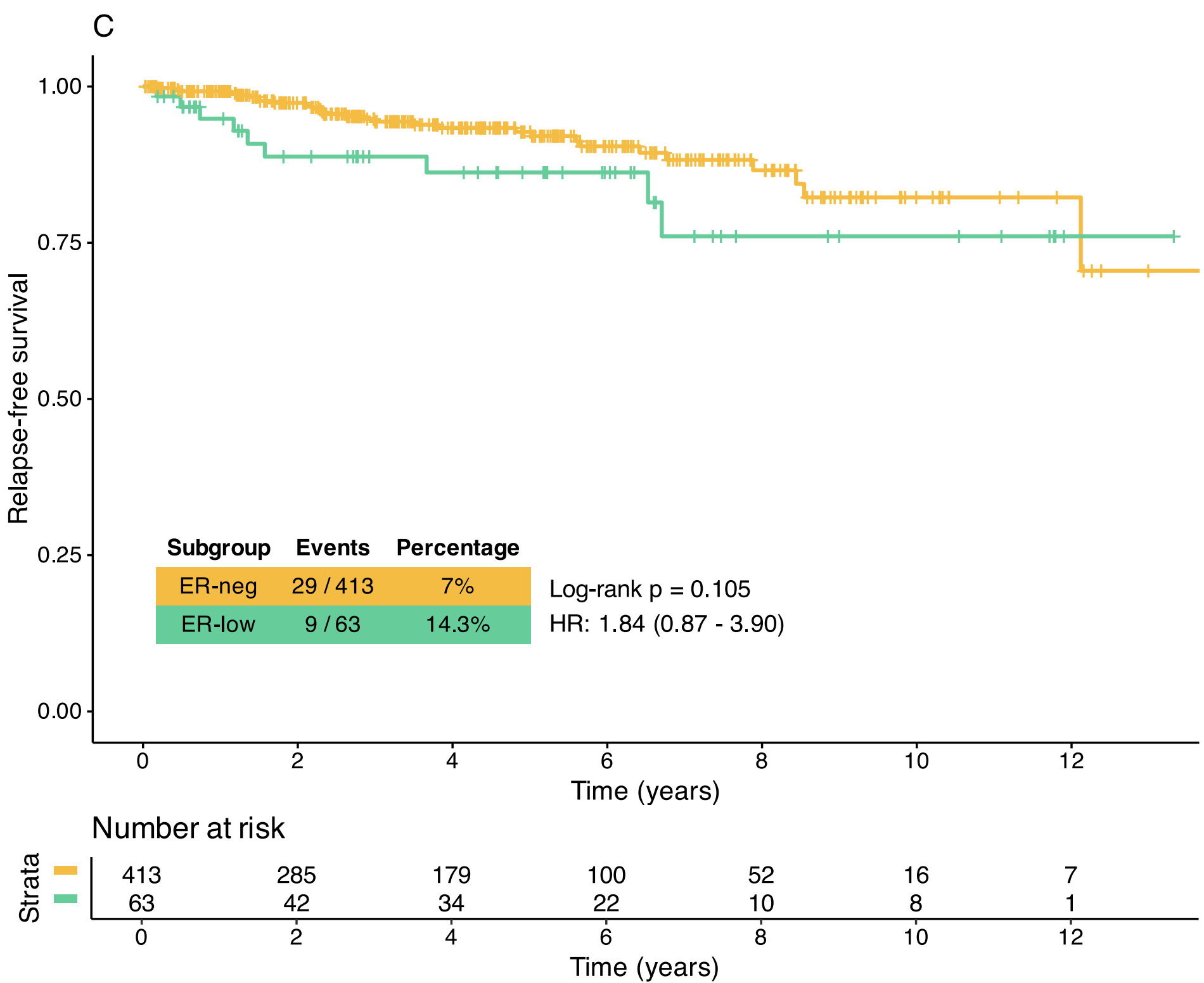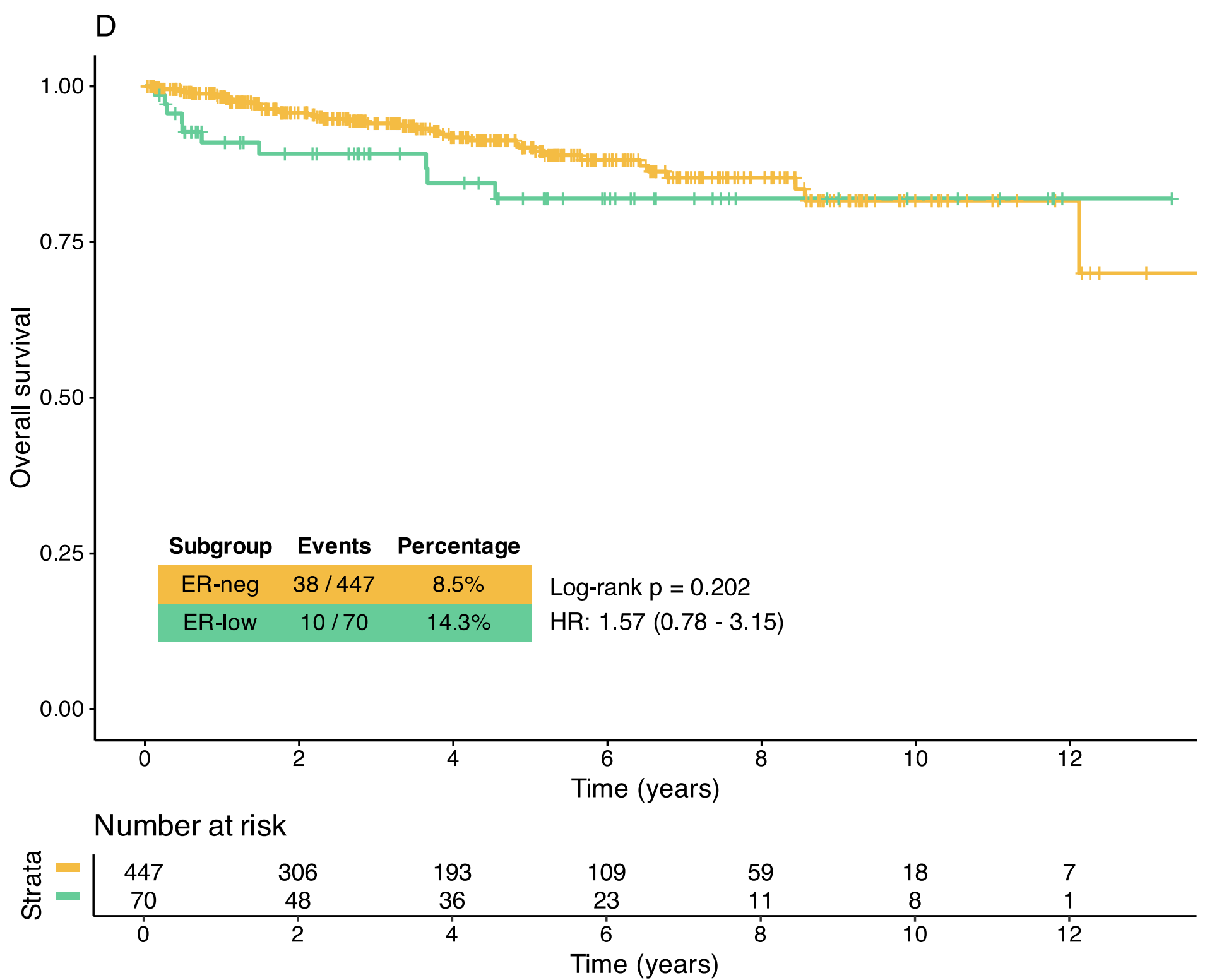

Supplement: djae178_Supplementary_Data [file djae178_supplementary_data.zip › djae178_Supplementary_Data/Supplementary Figure 2 - Revised.pdf]

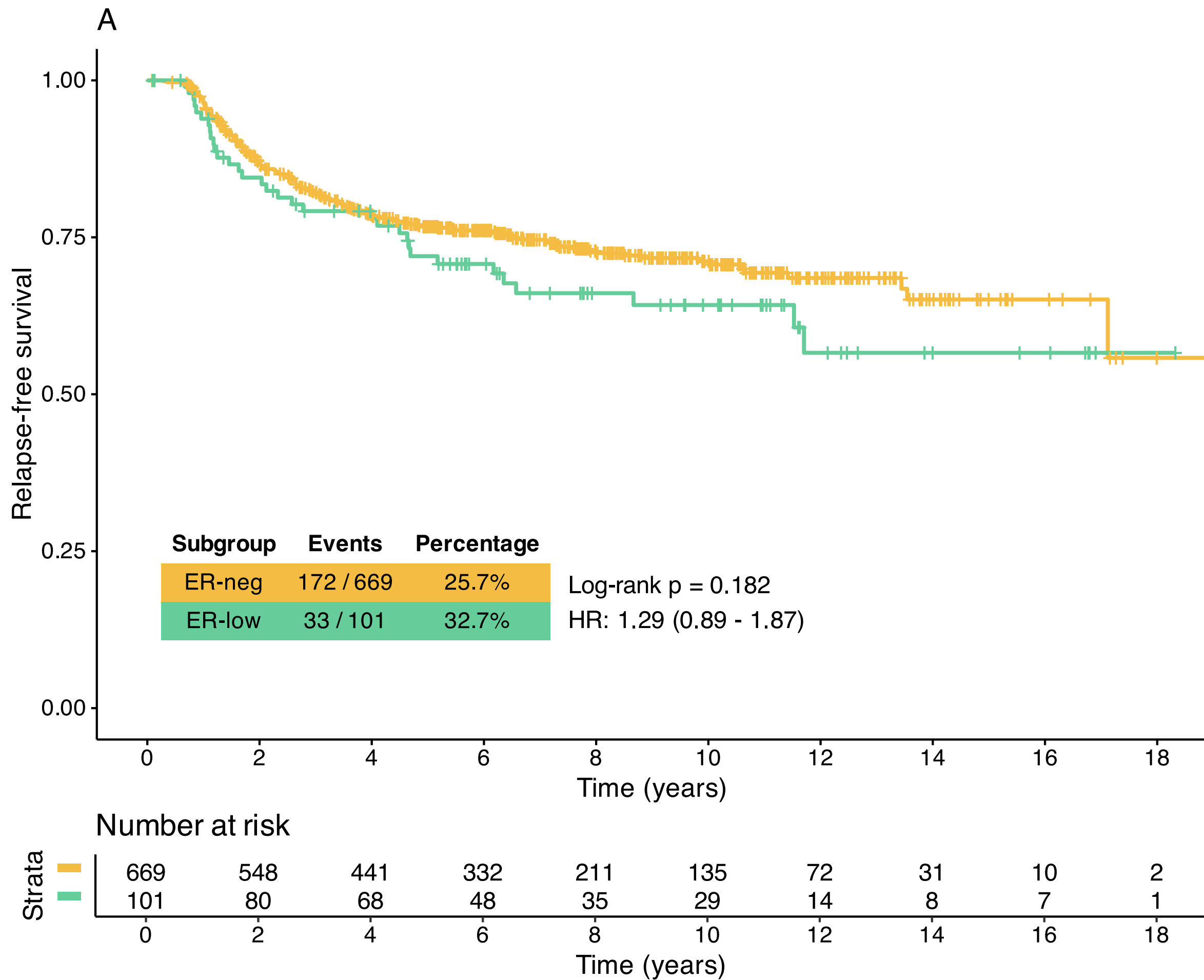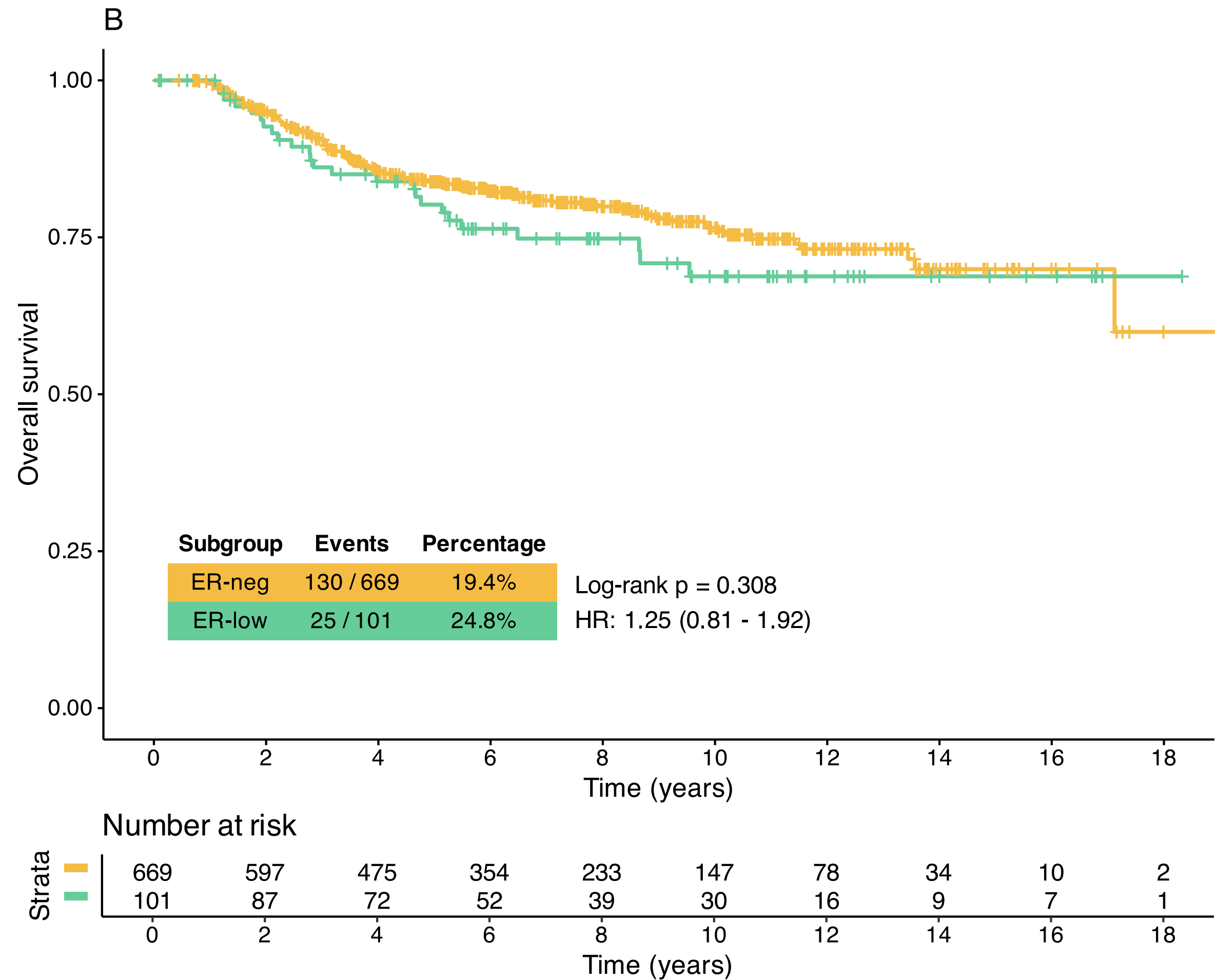

Supplement: djae178_Supplementary_Data [file djae178_supplementary_data.zip › djae178_Supplementary_Data/Supplementary Figure 3 - Revised.pdf]

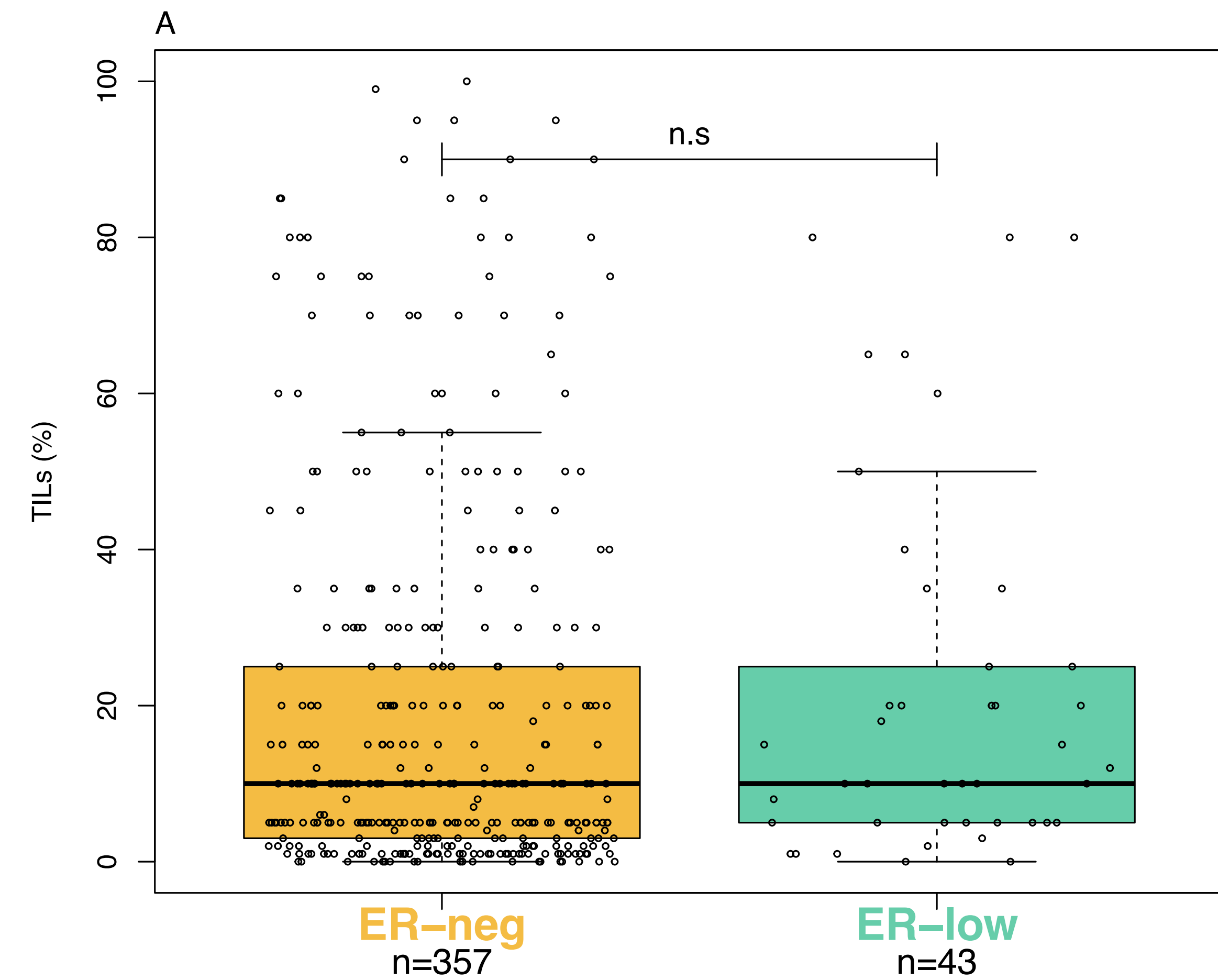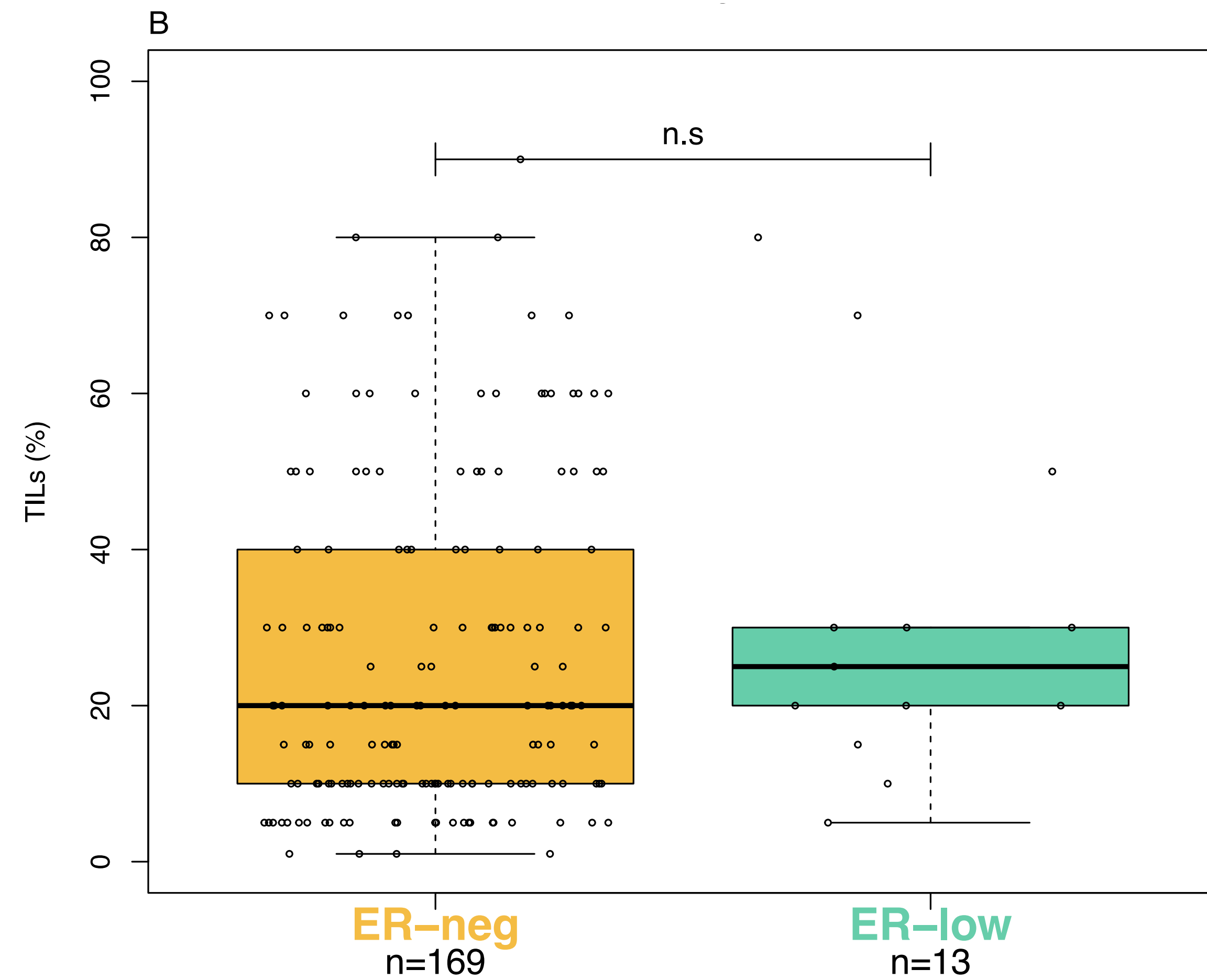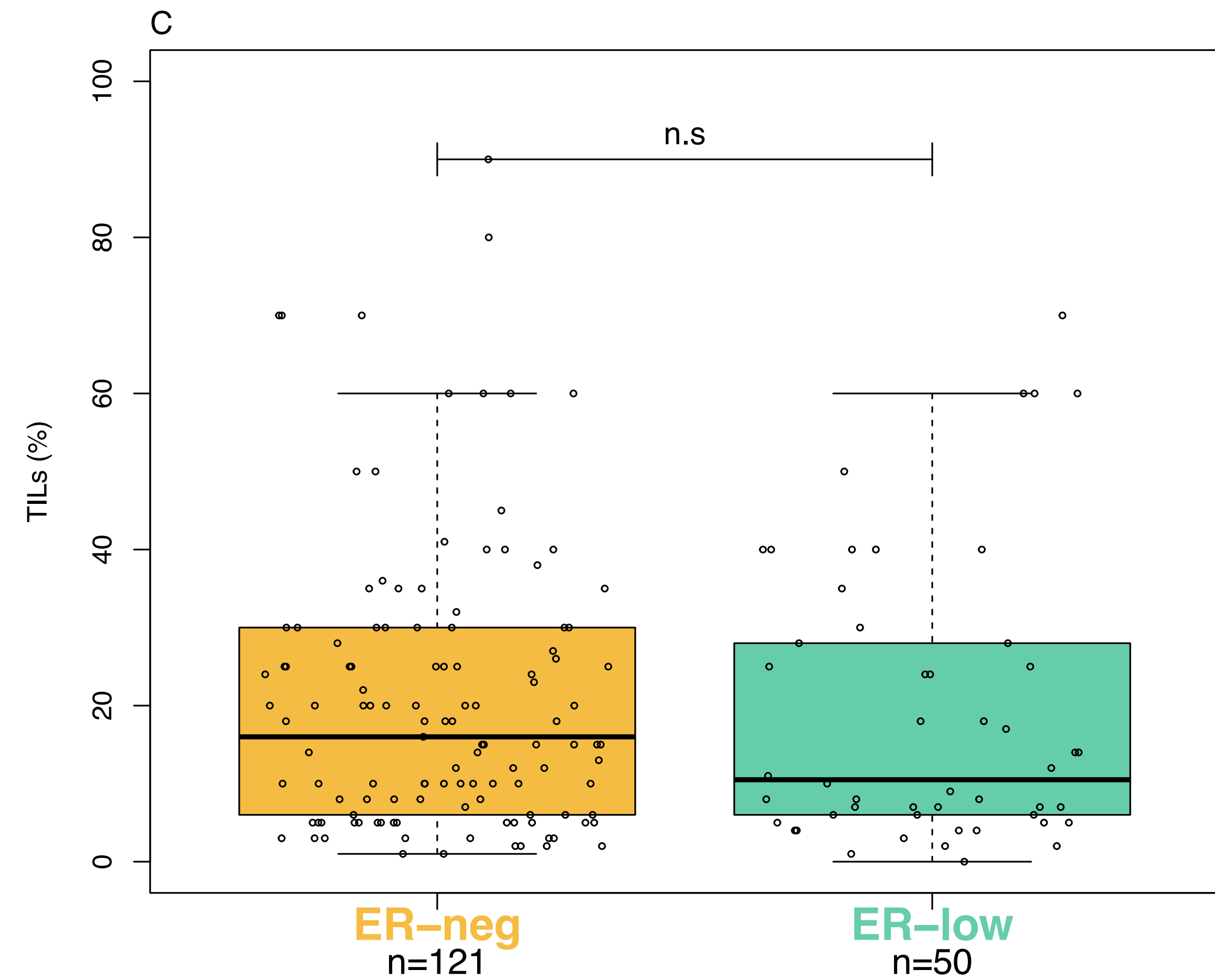

Supplement: djae178_Supplementary_Data [file djae178_supplementary_data.zip › djae178_Supplementary_Data/Supplementary Figure 4.pdf]

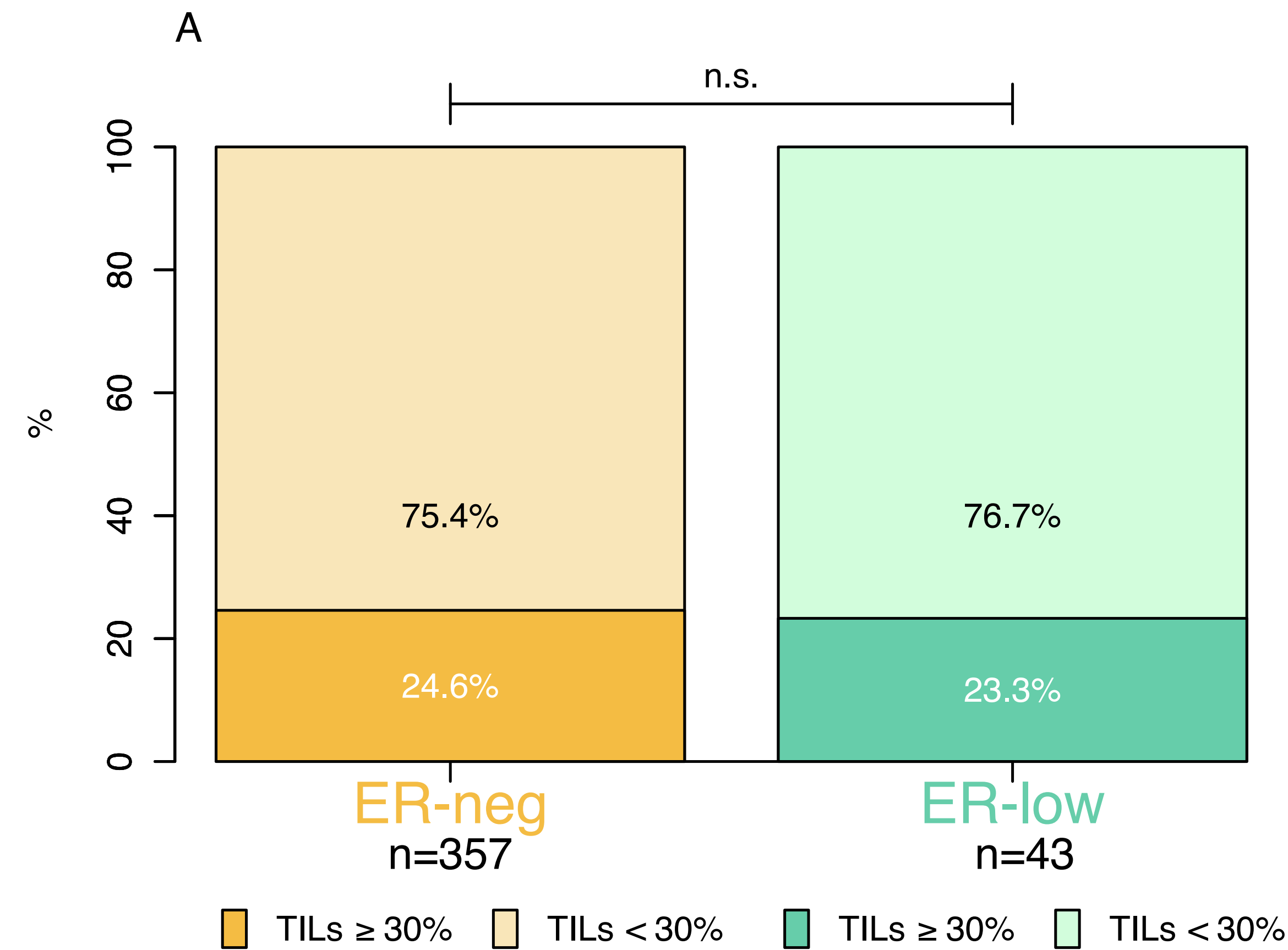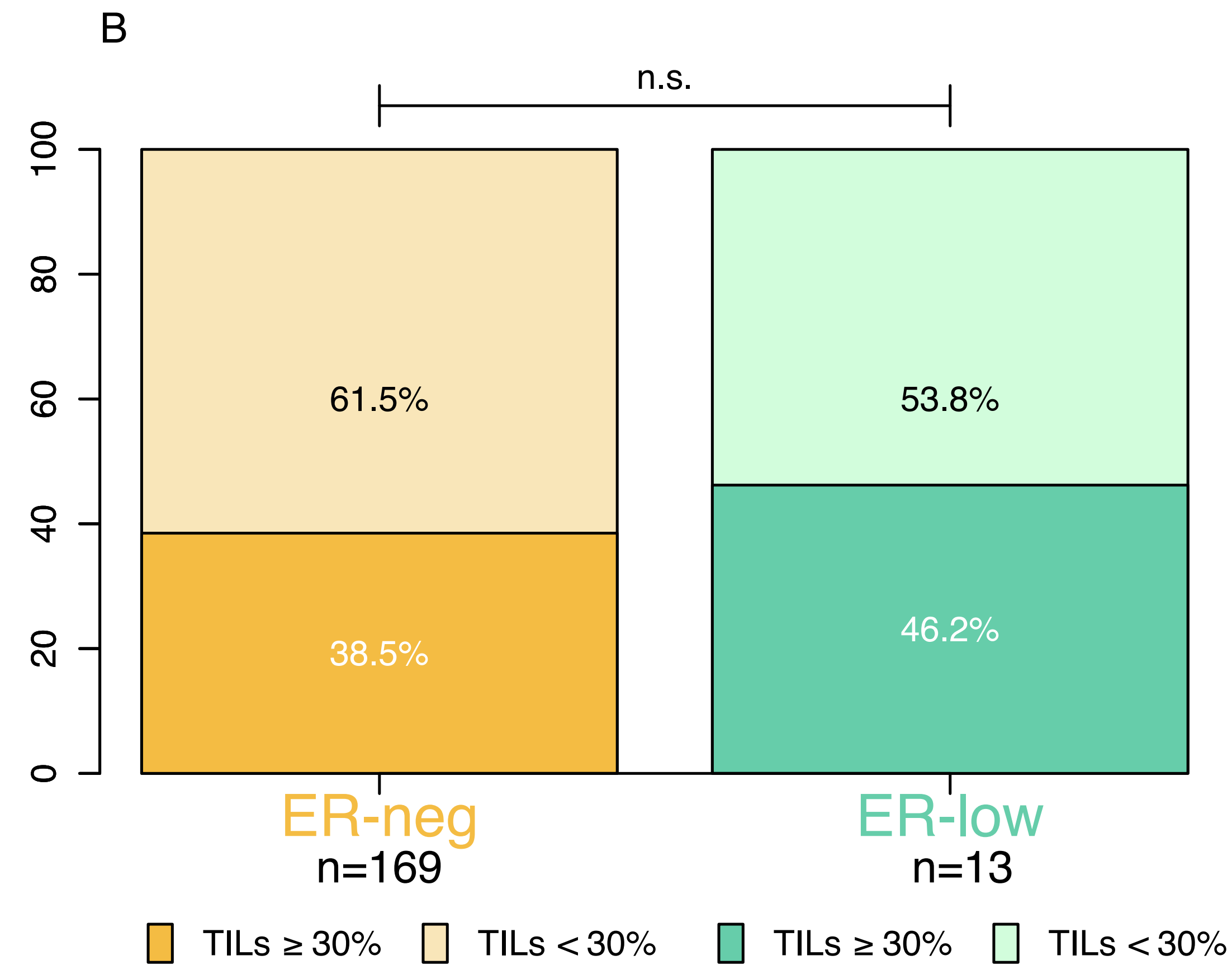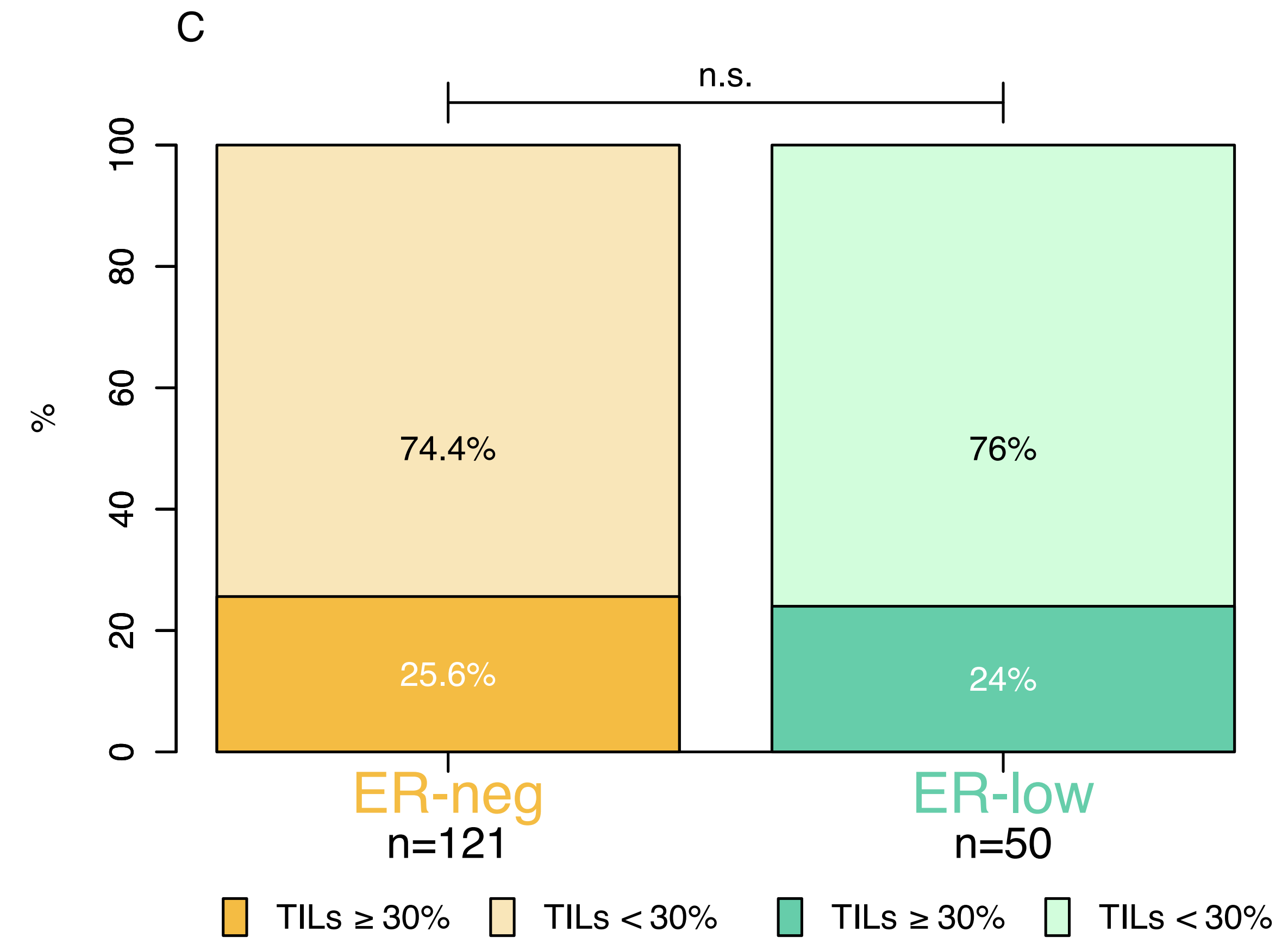

Supplement: djae178_Supplementary_Data [file djae178_supplementary_data.zip › djae178_Supplementary_Data/Supplementary Figure 5.pdf]

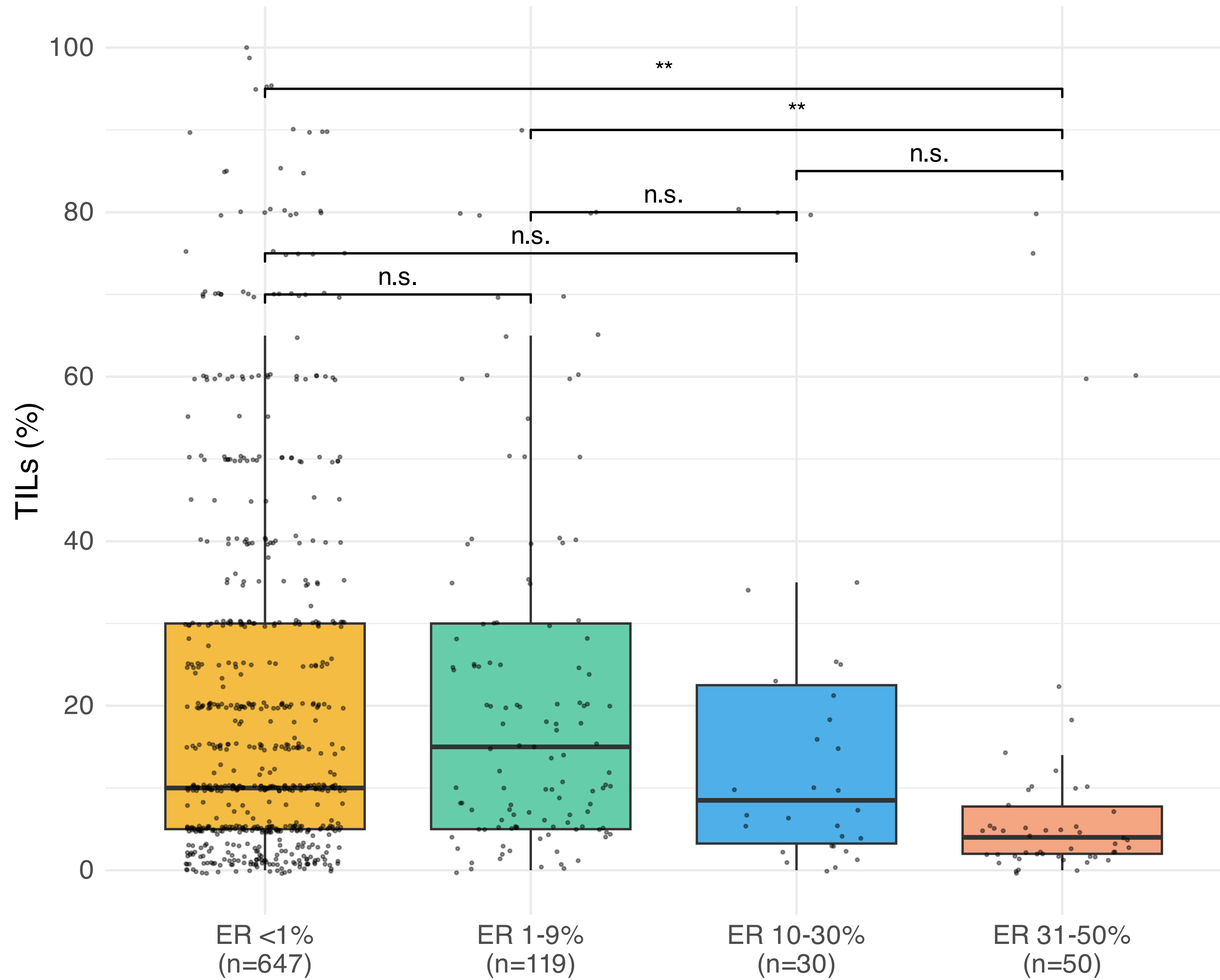

Supplement: djae178_Supplementary_Data [file djae178_supplementary_data.zip › djae178_Supplementary_Data/Supplementary Figure 6 - Revised.pdf]
